# Fluorescent Orthopalladated Complexes of 4-Aryliden-5(4*H*)-oxazolones from the Kaede Protein: Synthesis and Characterization

**DOI:** 10.3390/molecules26051238

**Published:** 2021-02-25

**Authors:** Eduardo Laga, David Dalmau, Sofía Arregui, Olga Crespo, Ana I. Jimenez, Alexandra Pop, Cristian Silvestru, Esteban P. Urriolabeitia

**Affiliations:** 1 Instituto de Síntesis Química y Catálisis Homogénea (ISQCH), CSIC-Universidad de Zaragoza, Pedro Cerbuna 12, E-50009 Zaragoza, Spain; edulala@hotmail.com (E.L.); 683763@unizar.es (D.D.); sofiaarreguip@gmail.com (S.A.); ocrespo@unizar.es (O.C.); anisjim@unizar.es (A.I.J.); 2 Supramolecular Organic and Organometallic Chemistry Centre, Departament of Chemistry, Faculty of Chemistry and Chemical Engineering, Babeş-Bolyai University, Str. Arany Janos 11, RO-400028 Cluj-Napoca, Romania; alepop@chem.ubbcluj.ro (A.P); cristian.silvestru@ubbcluj.ro (C.S.)

**Keywords:** oxazolones, Kaede protein, hula-twist, palladium, C–H bond activation, fluorescence

## Abstract

The goal of the work reported here was to amplify the fluorescent properties of 4-aryliden-5(4*H*)-oxazolones by suppression of the hula-twist non-radiative deactivation pathway. This aim was achieved by simultaneous bonding of a Pd center to the N atom of the heterocycle and the *ortho* carbon of the arylidene ring. Two different 4-((*Z*)-arylidene)-2-((*E*)-styryl)-5(4*H*)-oxazolones, the structures of which are closely related to the chromophore of the Kaede protein and substituted at the 2- and 4-positions of the arylidene ring (**1a** OMe; **1b** F), were used as starting materials. Oxazolones **1a** and **1b** were reacted with Pd(OAc)_2_ to give the corresponding dinuclear orthometalated palladium derivates **2a** and **2b** by regioselective C–H activation of the *ortho*-position of the arylidene ring. Reaction of **2a** (**2b**) with LiCl promoted the metathesis of the bridging carboxylate by chloride ligands to afford dinuclear **3a** (**3b**). Mononuclear complexes containing the orthopalladated oxazolone and a variety of ancillary ligands (acetylacetonate (**4a**, **4b**), hydroxyquinolinate (**5a**), aminoquinoline (**6a**), bipyridine (**7a**), phenanthroline (**8a**)) were prepared from **3a** or **3b** through metathesis of anionic ligands or substitution of neutral weakly bonded ligands. All species were fully characterized and the X-ray determination of the molecular structure of **7a** was carried out. This structure has strongly distorted ligands due to intramolecular interactions. Fluorescence measurements showed an increase in the quantum yield (QY) by up to one order of magnitude on comparing the free oxazolone (QY < 1%) with the palladated oxazolone (QY = 12% for **6a**). This fact shows that the coordination of the oxazolone to the palladium efficiently suppresses the hula-twist deactivation pathway.

## 1. Introduction

**Figure 1:**
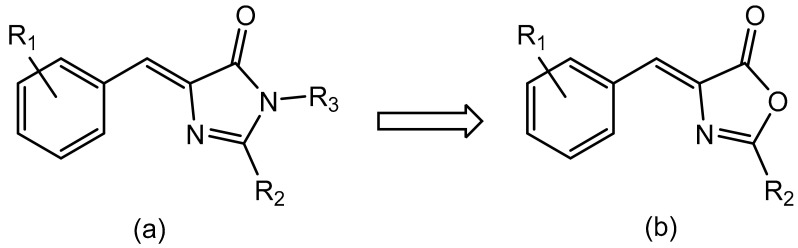
Schematic structures of 4-arylidene-5(4*H*)-imidazolones (**a**) and -oxazolones (**b**).

The synthesis of compounds with luminescent properties is currently one of the most active fields in chemical research, due to the applications of such materials in a variety of fields such as opto-electronic devices (cell phones, smart TVs, tablets and others), biological markers, sensors and many others [1,2,3,4,5,6,7]. Oxazolones and imidazolones are promising candidates for the preparation of compounds with remarkable luminescent properties and this is based on the fact that the chromophore of the green fluorescent protein (GFP) is a 4-arylidene-5(4*H*)-imidazolone (Figure 1a). The GFP is a protein of great importance because the expression of proteins bonded to the GFP allows fluorescent labeling inside cells. Due to these properties, the GFP is considered to be the microscope of the 21st century [8,9,10,11]. 4-Arylidene-5(4*H*)-oxazolones are closely related to imidazolones, which are precursors of the former (Figure 1b). Unsaturated oxazolones are also luminescent and they have additional remarkable photophysical properties that include two-photon absorption (TPA), up-conversion, nonlinear optical properties and unique behavior as molecular switches [12,13,14,15,16,17,18,19,20].

The luminescent properties of both unsaturated oxazolones and imidazolones are strongly dependent on the environment. When the imidazolone chromophore is removed from the confined space of the GFP, the quantum yield of the imidazolone drops by four orders of magnitude, thus evidencing the critical influence that the rigid environment has on the photophysical properties. Similarly, the quantum yields of oxazolones in solution are usually very low (10^−2^–10^−4^) and marked increases have been observed in solid state or at low temperature [21]. The reasons for this behavior have been studied in detail and it seems to be due to the presence of non-radiative decay channels, as an alternative to fluorescence, such as *Z-E* isomerization (amongst others). This isomerization can take place through different internal conversions: rotation around the C=C bond and/or a combination of the rotation around the C=C bond with rotation around the C–C bond, movement that is also called a hula-twist. There is an intense debate about the precise mechanism in action for each particular case, and research work is ongoing in this active area [22,23,24,25,26,27,28,29,30,31,32,33,34,35,36,37,38,39,40,41,42,43,44,45]. In other cases, the loss of fluorescence has been related to the energy gap between the stabilized excited state and the ground state [46].

**Figure 2:**
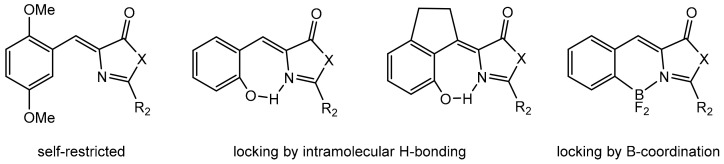
Different strategies in order to avoid non-radiative loss of fluorescence: self-restriction and molecular locks to constrain twisting internal motions.

Different approaches have been proposed to avoid the loss of fluorescence and these include the introduction of substituents at specific positions to promote self-restriction (Figure 2, left) or the establishment of intramolecular locks (Figure 2, middle and right) [47,48,49,50,51,52,53,54,55,56,57,58,59,60,61,62]. Among these approaches, the most successful one is probably the introduction of a bridge between the *ortho*-position of the 4-arylidene ring and the N atom of the heterocycle. The use of either a hydrogen bond or a BF_2_ group provides an efficient lock of the internal motions and eliminates the non-radiative channels, with impressive increases in the fluorescence reported [47,48,49,50,51,52,53,54,55,56,57,58,59,60,61,62].

**Figure 3:**
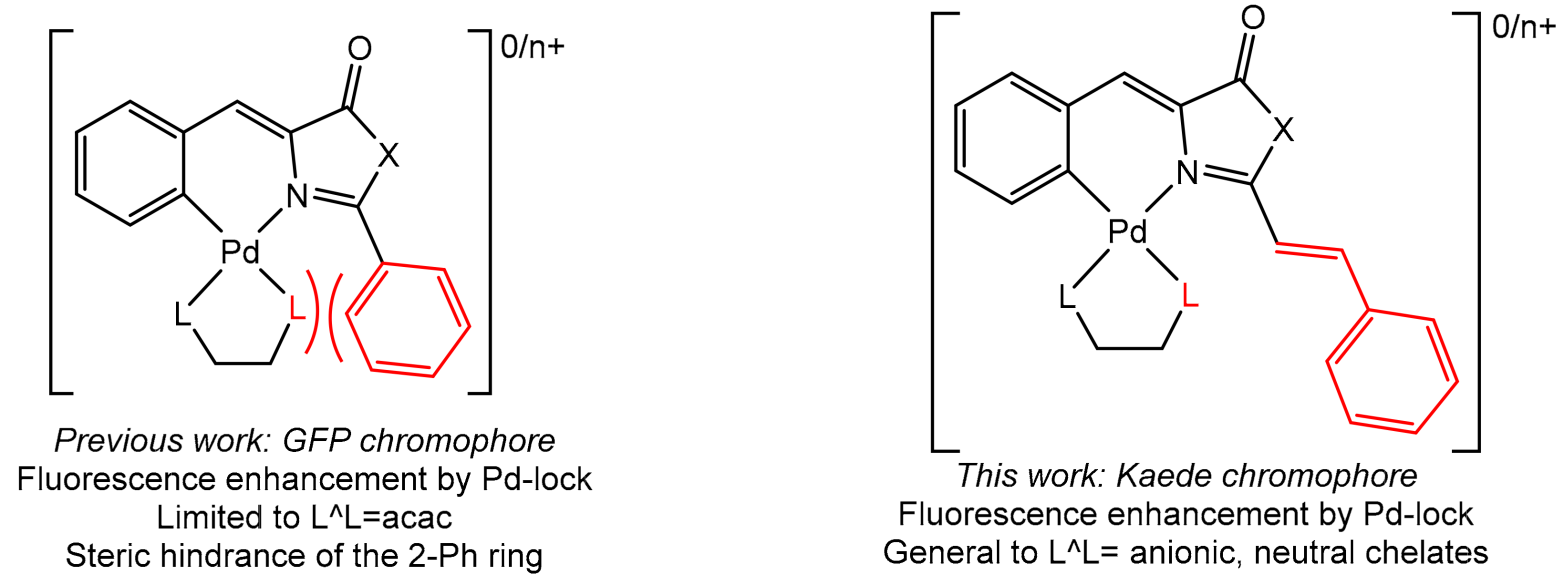
Previous work on the Pd-locked green fluorescent protein (GFP) chromophore, showing limited reactivity; comparison with the present work with the Kaede protein, showing a more general scope of reactivity.

Despite the outstanding results, other reports on the amplification of the luminescence by locking of oxazolones through *ortho*-functionalization have not been published. We recently described a contribution in this area in which palladium complexes were employed as an intramolecular lock, as shown in Figure 3 (left) [63]. The incorporation of the Pd(II) lock into the skeleton of (*Z*)-4-arylidene-2-phenyl-5(4*H*)-oxazolones and -imidazolones was achieved using C–H activation processes by methods developed by our group [64,65,66,67,68]. The acetylacetonate ligand was used as an ancillary group to complete the coordination sphere of the Pd center. The results showed that this type of Pd lock was effective and led to increases in the quantum yield (QY) of push–pull imidazolones by up to one order of magnitude [63]. With the aim of achieving additional tuning of the fluorescence by changing the ancillary ligands bonded to the palladium, several attempts were made to coordinate chelating ligands L^L other than acetylacetonate. However, all attempts to obtain more general structures were unsuccessful. It is likely that the steric hindrance resulting from the presence of the Ph ring in the 2-position of the heterocycle, which is in close proximity to the Pd-bonded L^L ligand, is the cause of this lack of reactivity. Therefore, only L^L ligands with low steric requirements (acac) were bonded to the Pd center to give stable complexes. In this respect, we previously observed markedly different behavior of C^N-chelating oxazolones with respect to other classical C^N chelates in the reactivity of such complexes towards bulky monodentate ligands such as phosphines [69].

**Scheme 1:**
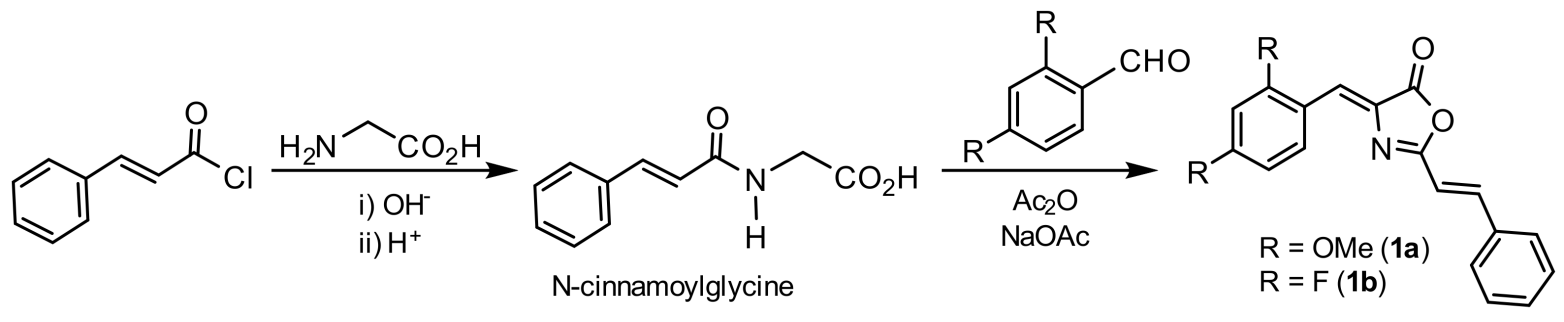
Synthesis of starting oxazolones **1a** and **1b.**

In this publication, we present recent results obtained on using oxazolones **1a** and **1b**, which are related to the chromophore of the Kaede protein (Figure 3, right, and Scheme 1) [70,71,72]. Due to the presence of the (*E*)-styryl group in the 2-position of the heterocycle, this molecule is sterically less hindered than the 2-phenyl analog and the larger volume around the Pd center is expected to accommodate incoming ligands. In addition, the extended conjugation due to the 2-styryl fragment should red-shift the fluorescence, thus providing a wider range of emission wavelengths. The Kaede protein chromophore shows a much more intense fluorescence when compared with the GFP chromophore [70,71,72]. We selected OMe (**1a**) and F (**1b**) as 4-arylidene substituents for different reasons. On the one hand, the OMe group is strongly electron-donating and is expected to boost the charge transfer throughout the oxazolone (push–pull effect). On the other hand, it has been shown that F substituents promote additional red-shifts [59,60]. Furthermore, the substituents located at the 2- and 4-positions seem to maximize the charge transfer [46]. Overall, one would expect a less constrained orthopalladated moiety that is able to accommodate a wide range of auxiliary ligands while having a more conjugated system that is red-shifted and much more intense. The combination of a strongly emitting oxazolone ligand with the suppression of the hula-twist due to locking by orthopalladation should produce highly fluorescent systems. The results obtained on studying these systems are described below.

## 2. Results and Discussion

### 2.1. Synthesis, Characterization and Orthopalladation of Oxazolones **1a** and **1b**

The oxazolones **1a** and **1b** were prepared by the Erlenmeyer–Plöch method by reaction of *N*-cinnamoylglycine with the respective aldehyde (Scheme 1) [73,74,75,76,77,78,79]. The *N*-cinnamoylglycine was prepared by the Schotten–Baumann method [80].

**Figure 4:**
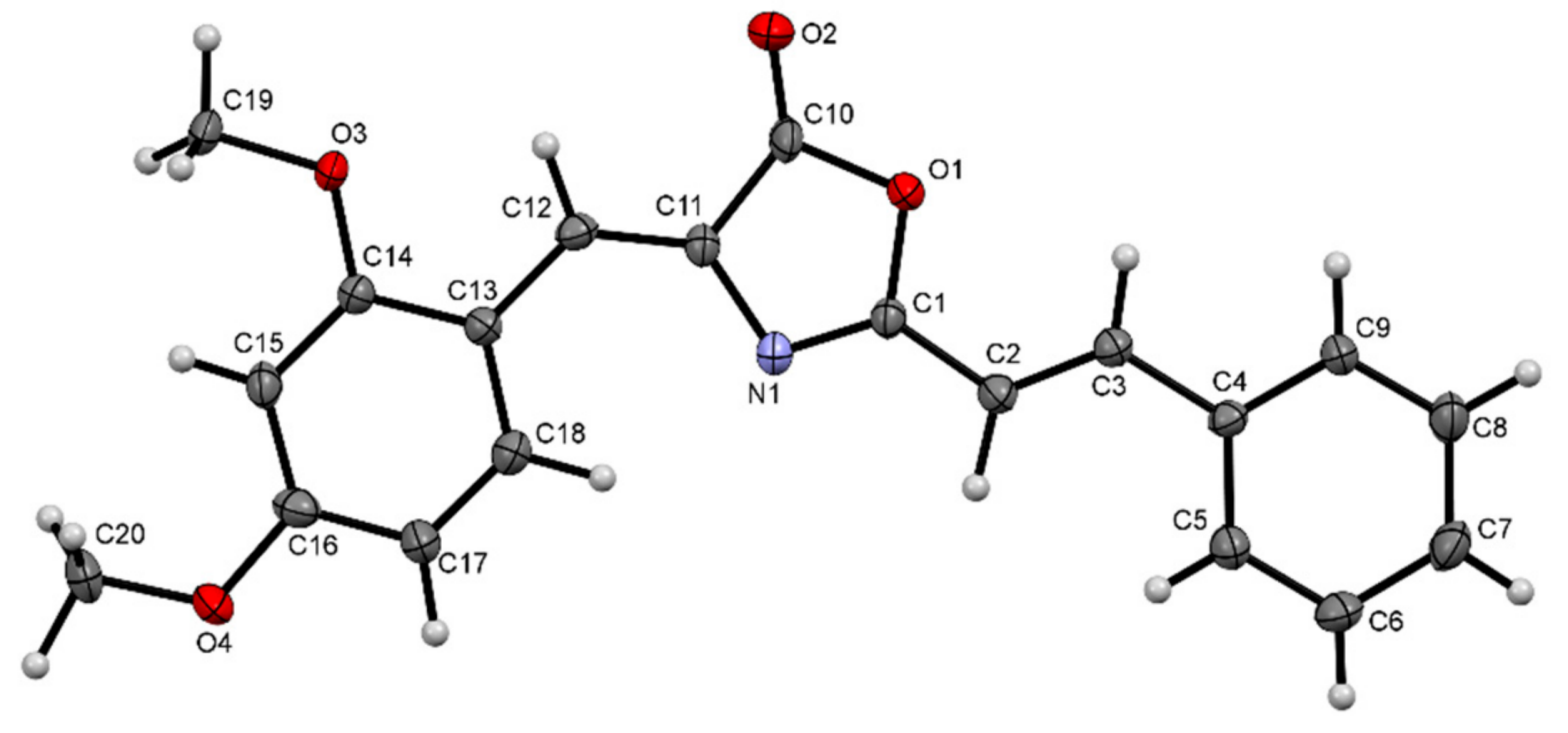
Molecular drawing of oxazolone **1a.**

Oxazolones **1a** and **1b** were characterized by mass spectrometry and NMR methods. The X-ray structure of **1a** was also determined. The mass spectra of **1a** and **1b** confirmed the stoichiometry shown in Scheme 1. The ^1^H NMR spectra of **1a** and **1b** show the expected AMX spin systems for the C_6_H_3_ ring, as well as signals due to the styryl C(H)=C(H)C_6_H_5_ unit. The values of the ^3^*J*_HH_ coupling constants (around 16.5 Hz) between the two olefinic protons are consistent with a *trans* arrangement of these protons and the *E* geometry for this C=C bond. The observation of the carbonyl carbon in the proton-coupled ^13^C NMR spectra as a doublet with a ^3^*J*_CH_ coupling constant of 5.2 Hz shows that the exocyclic C(H)=C bond has the (*Z*) configuration [81]. The structure of **1a** could be solved (Figure 4) but the data gathered were of poor quality. Therefore, a complete analysis of bond distances and angles could not be performed, and the structure was used only as a connectivity scheme. In any case, the determined structure and the solution NMR data are in full agreement.

**Scheme 2:**
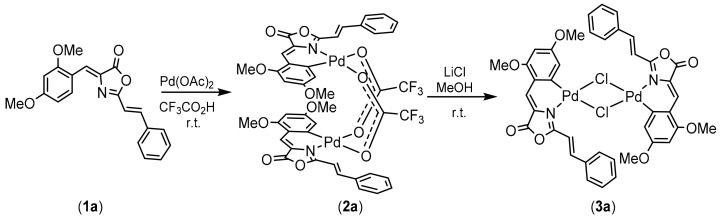
Orthopalladation of oxazolone **1a** and reactivity of orthometalated derivative **2a**: synthesis of oxazolone derivative **3a.**

In order to incorporate the Pd center into the oxazolone skeleton, the reactivity of **1a** and **1b** with Pd(OAc)_2_ (1:1 molar ratio) in CF_3_CO_2_H was evaluated by previously published methods [63,64,65,66,67,68]. In the case of the electron-rich oxazolone **1a**, the reaction took place at room temperature to afford dimer **2a** in good yield (94%) (see Scheme 2). The dinuclear nature of the product was confirmed by the mass spectrum of **2a**. The orthopalladation was regioselective at the 6-position of the 4-arylidene ring, as inferred from the observation of an AB spin system (6.23 and 6.17 ppm, ^4^*J*_HH_ = 2 Hz) in the ^1^H NMR spectrum of **2a**, which corresponds to the C_6_H_2_ palladated ring. This selectivity is the same as that observed previously in related systems [63,64,65,66,67,68]. Additional signals due to the presence of the styryl moiety and the vinyl proton were also evident. The *anti* geometry of dimer **2a** is suggested by the observation of a single peak for the C**F_3_**CO_2_ bridges in the ^19^F NMR spectrum. The data discussed above are consistent with the structure shown in Scheme 2 for **2a**.

**Scheme 3:**
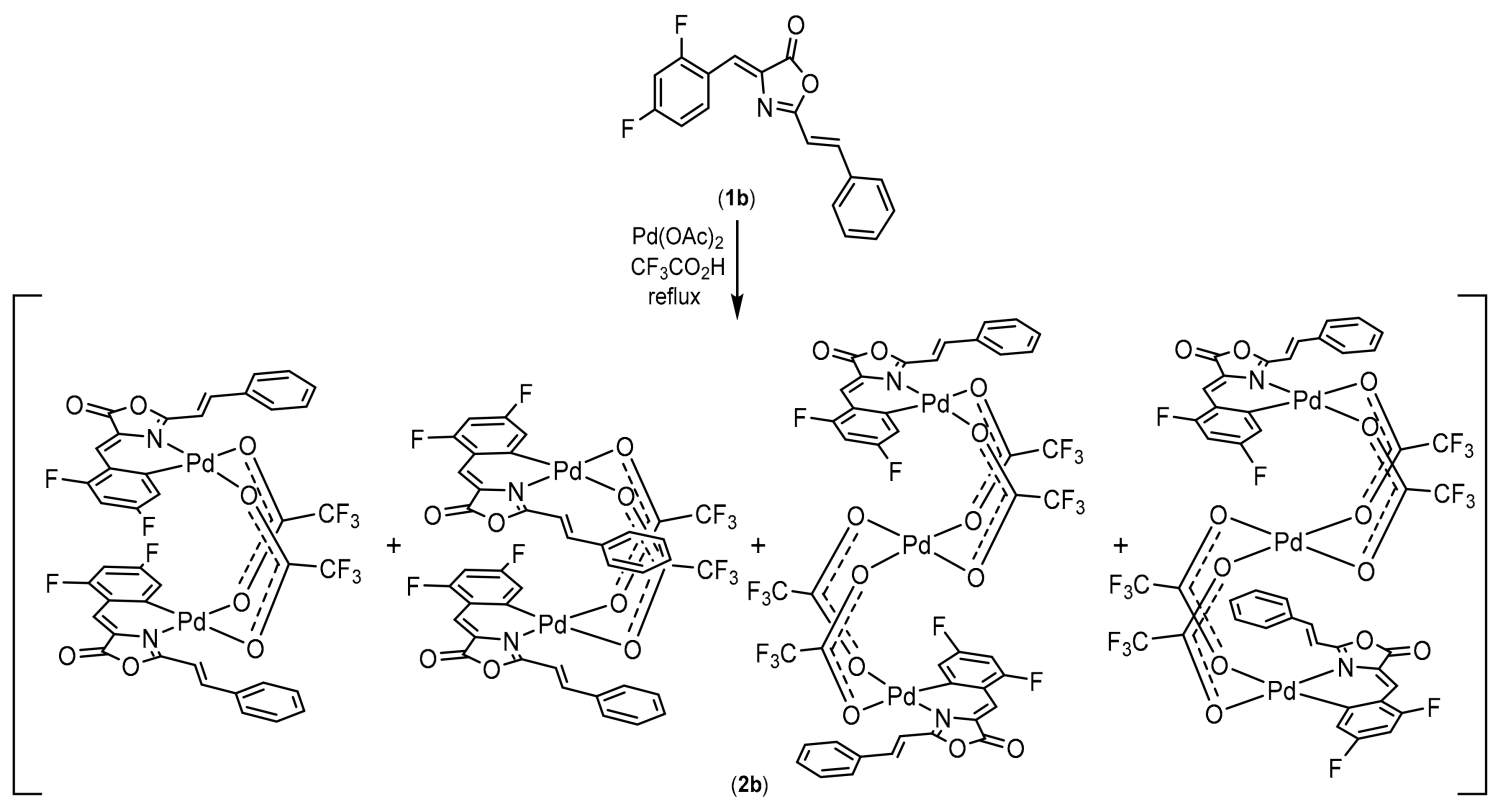
Orthopalladation of oxazolone **1b.**

The reactivity of **1b** towards Pd(OAc)_2_ (1:1 molar ratio) follows a slightly different pathway. The reaction requires reflux temperature for four hours in order to achieve full conversion. However, despite the complete consumption of **1b**, the ^1^H and, more clearly, the ^1^H{^19^F} NMR spectra of **2b** show that it is a mixture of four different orthopalladated complexes. In this respect, four different AB spin systems, quite similar to that observed for **2a**, appear for **2b** in the region 6.4–6.8 ppm, thus showing that the four complexes contain the C_6_H_2_ ring. Analysis of the ^19^F NMR spectrum of **2b** also clearly shows the presence of four types of C_6_H_2_F_2_ rings in the molar ratio 1/0.6/0.4/0.3, together with signals assigned to the corresponding CF_3_CO_2_ ligands. The dinuclear (*anti*- and *syn*-) and trinuclear (also *anti*- and *syn*-) structures shown in Scheme 3 are in good agreement with the experimental findings. The formation of mixtures of dinuclear and trinuclear derivatives in F-substituted oxazolones has recently been described by us [67]. Trinuclear structures are not often found when dealing with orthopalladation processes, despite their relevance both in synthesis and catalysis [82,83]. In order to confirm the presence of species of different nuclearity, ^1^H DOSY experiments were carried out and the attenuation of the signals corresponding to the C_6_H_2_ fragment and those assigned to the olefinic proton was evaluated. The values obtained for the diffusion coefficient (D, m^2^s^−1^) are D = 1.00 × 10^−9^ and D = 9.12 × 10^−10^ m^2^s^−1^. The different D values show that the mixture is composed of complexes of different molecular sizes and, therefore, of different nuclearity, with the smaller D values associated with the larger trinuclear derivatives. 

Further treatment of dinuclear **2a** or the mixture **2b** with excess LiCl in methanol at room temperature promoted the metathesis of the carboxylate bridges by chloride bridges to afford dinuclear derivative **3a** (Scheme 2) or **3b** (Scheme 5, see below). These types of chloride-bridged derivatives are generally good starting materials for the synthesis of other cyclometalated complexes containing a variety of ancillary ligands. The characterization of **3a** and **3b** could not be performed by NMR methods due to their lack of solubility, even in DMSO-*d*_6_. However, **3a** and **3b** gave elemental analysis results that were consistent with the proposed stoichiometries and bands were observed in the IR spectra assigned to the Pd–Cl stretch (see Materials and Methods and Supplementary Materials) [84].

### 2.2. Synthesis of Complexes with Orthopalladated Oxazolones and Different Ancillary Ligands

**Scheme 4:**
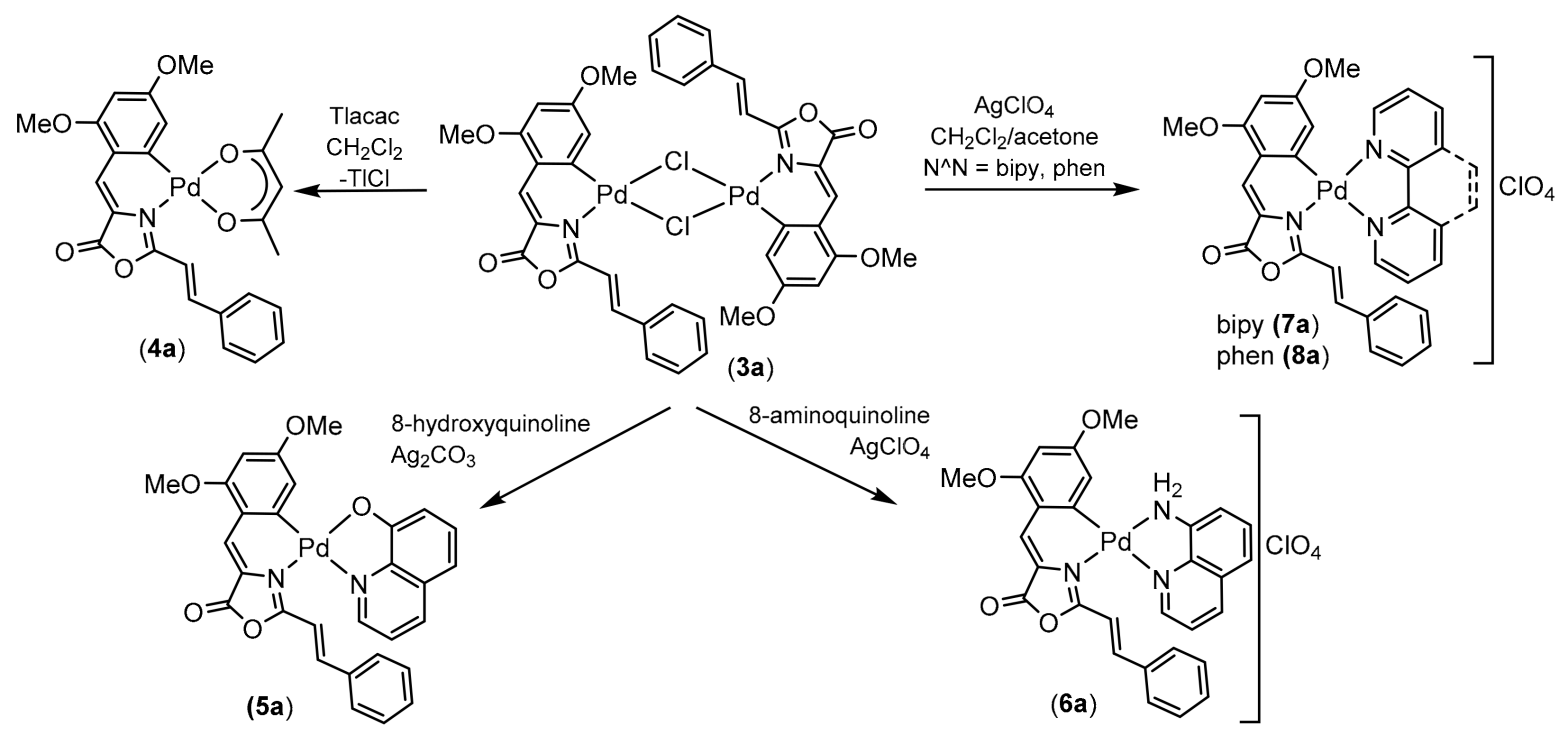
Reactivity of **3a** to give orthopalladated derivatives **4a**–**8a.**

**Scheme 5:**
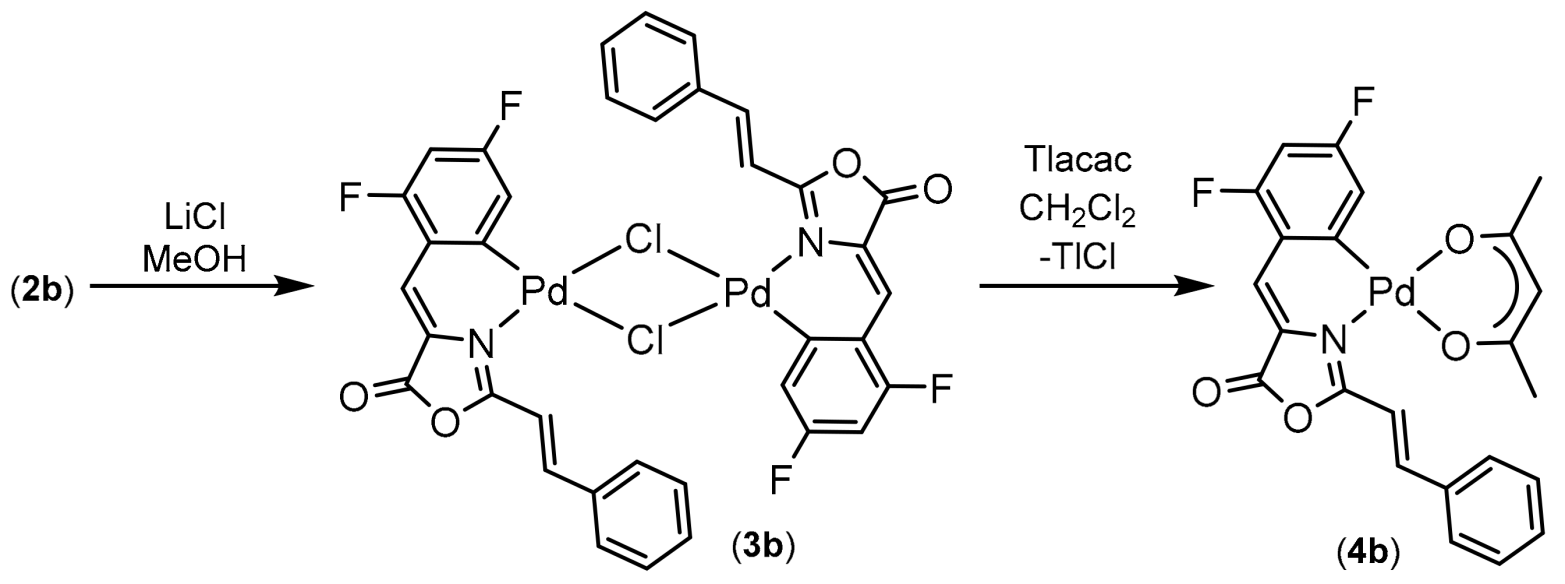
Reactivity of **2b** to give orthopalladated derivatives **3b** and **4b.**

Attempts to study the photophysical properties of complexes **2a**, **3a** and **3b** were unsuccessful. Complex **2a** did not show emission, probably due to the flexibility of the carboxylate bridges, while **3a** and **3b** were completely insoluble in all organic solvents tested. As a consequence, it was decided to prepare mononuclear derivatives with different types of chelating ligands with the aim of increasing the solubility and the stability of the resulting bis-chelate derivatives, thus minimizing the deactivation pathways related to weakly bonded ligands. In addition, anionic and neutral chelating ligands with different donor atoms were selected in order to cover a wider range of structural situations and to study the influence that the global charge and the nature of the donor atoms have on the photophysical properties. Acetylacetonate and 8-hydroxyquinolinate were selected as O,O’- and N,O-anionic chelating groups, while 8-aminoquinoline, 2,2-bipyridine and 1,10-phenanthroline were representatives of N,N-neutral ligands. In all cases, the syntheses were performed following well-established procedures involving metathesis of anionic ligands or exchange of weakly coordinated neutral ligands, as shown in Scheme 4 and Scheme 5.

Treatment of **3a** or **3b** with Tl(acac) (1:2 molar ratio, acac = acetylacetonate) in CH_2_Cl_2_ at 25 °C gave the corresponding O,O’-acac derivatives **4a** or **4b** as deep orange solids, while reaction of **3a** with 8-hydroxyquinoline in the presence of Ag_2_CO_3_ (1:2:2 molar ratio) afforded the red N,O-hydroxyquinolinate **5a**. All complexes were obtained in good yields. Treatment of **3a** with AgClO_4_ (1:2 molar ratio) in a mixture of CH_2_Cl_2_/acetone gave, after removal of the insoluble AgCl, a solution of the solvate [Pd(C,N-oxazolone)(acetone)_x_]ClO_4_. This solution was reacted with 8-aminoquinoline, 2,2-bipyridine or 1,10-phenanthroline (1:1 molar ratio) to afford the corresponding cationic derivatives [Pd(C,N-oxazolone)(N^N)]ClO_4_ (8-NH_2_quin **6a**, bipy **7a**, phen **8a**) in good yields as bright red solids (Scheme 4). 

The characterization of **4a**–**8a** and **4b** was carried out by HR mass spectrometry and NMR spectroscopy. Signals due to the orthopalladated oxazolones were clearly identified in the NMR spectra of all of the complexes. In the case of acac derivatives, additional peaks in the ^1^H NMR spectra of **4a** and **4b** were observed at around 5.5 (CH) and 1–2 ppm (CH_3_), and in the ^13^C NMR spectra at around 96 (CH), 187 (CO) and 27 ppm (CH_3_). These signals are due to the presence of the O,O’-chelated acac group. In the case of complexes **5a** and **6a**, two different geometric isomers were expected due to the presence of different donor atoms in the ancillary ligands. However, the ^1^H and ^13^C NMR spectra of each complex contained a single set of peaks, thus showing that they were obtained as single isomers. The assignment of all resonances was performed by COSY, HSQC and HMBC experiments. In addition, selective 1D-NOESY experiments on **6a** (see Supplementary Materials) allowed the geometry of the obtained isomer to be determined. The inversion of the two protons of the orthopalladated C_6_H_2_ ring only showed clear NOE enhancements on the near OMe groups, so these experiments were inconclusive. However, inversion of the *ortho* proton of the pyridine ring resulted in a clear NOE effect on one of the olefinic protons of the styryl fragment, thus showing the proximity of the two moieties, as shown in Scheme 4. In turn, inversion of one of the olefinic protons promoted a strong NOE effect on the *ortho* proton of the pyridine ring as well as on the *ortho* protons of the Ph ring of the styryl unit. Characteristic peaks due to the presence of bipyridine and phenanthroline were also observed in the NMR spectra of **7a** and **8a** (SM).

**Figure 5:**
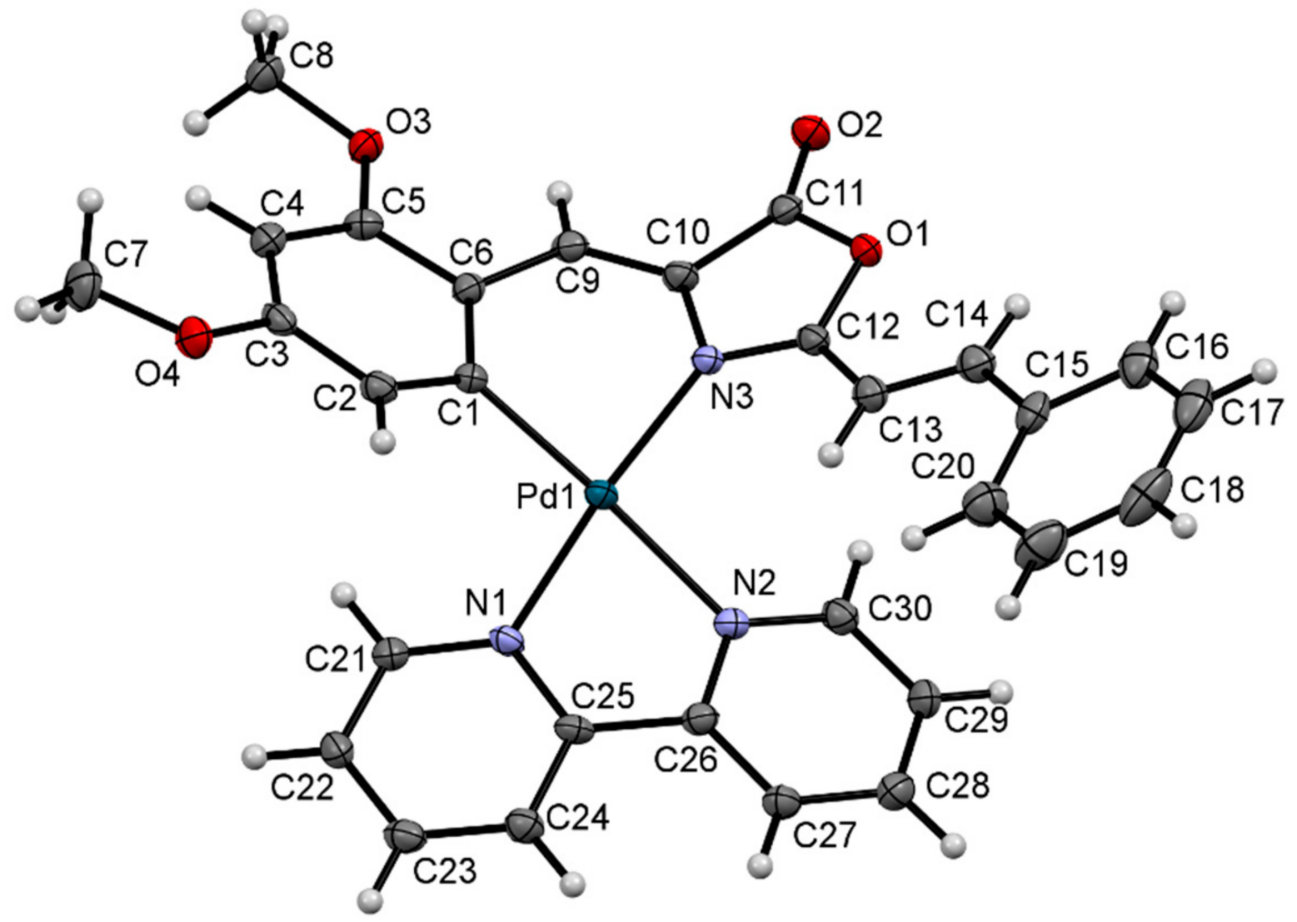
Molecular drawing of complex **7a.**

The molecular structure of complex **7a** was determined and this provided valuable information. A drawing of the cationic part of the complex is shown in Figure 5 and selected bond distances and angles are collected in Table 1. Crystallographic parameters concerning the data collection and structure solution are provided in the Experimental section and in the Supporting Information. In the complex, the Pd atom is in a slightly distorted square-planar environment (sum of angles around the Pd atom = 360.05°) and is bonded to the two N atoms of the bipy ligand, N1 and N2, to the N3 atom of the oxazolone heterocycle and to the *ortho* carbon C1 of the arylidene ring. Thus, orthopalladation of the chromophore of the Kaede protein leaves sufficient space to bond incoming ligands that are bigger than acetylacetonate, thus improving on previous results obtained with the chromophore of the GFP [63]. The Pd1–N2 bond distance [2.110(3) Å] is longer than the Pd1–N1 bond distance [2.060(3) Å] and this is due to the higher *trans* influence of the C1 atom *trans* to N2 with respect to the N3 atom *trans* to N1, although all Pd–N bond distances fall in the usual range of values found in the literature for this type of bond [85]. The Pd1–C1 bond distance [2.013(3) Å] is also typical for this type of bond [64,65,66,67,68].

**Figure 6:**
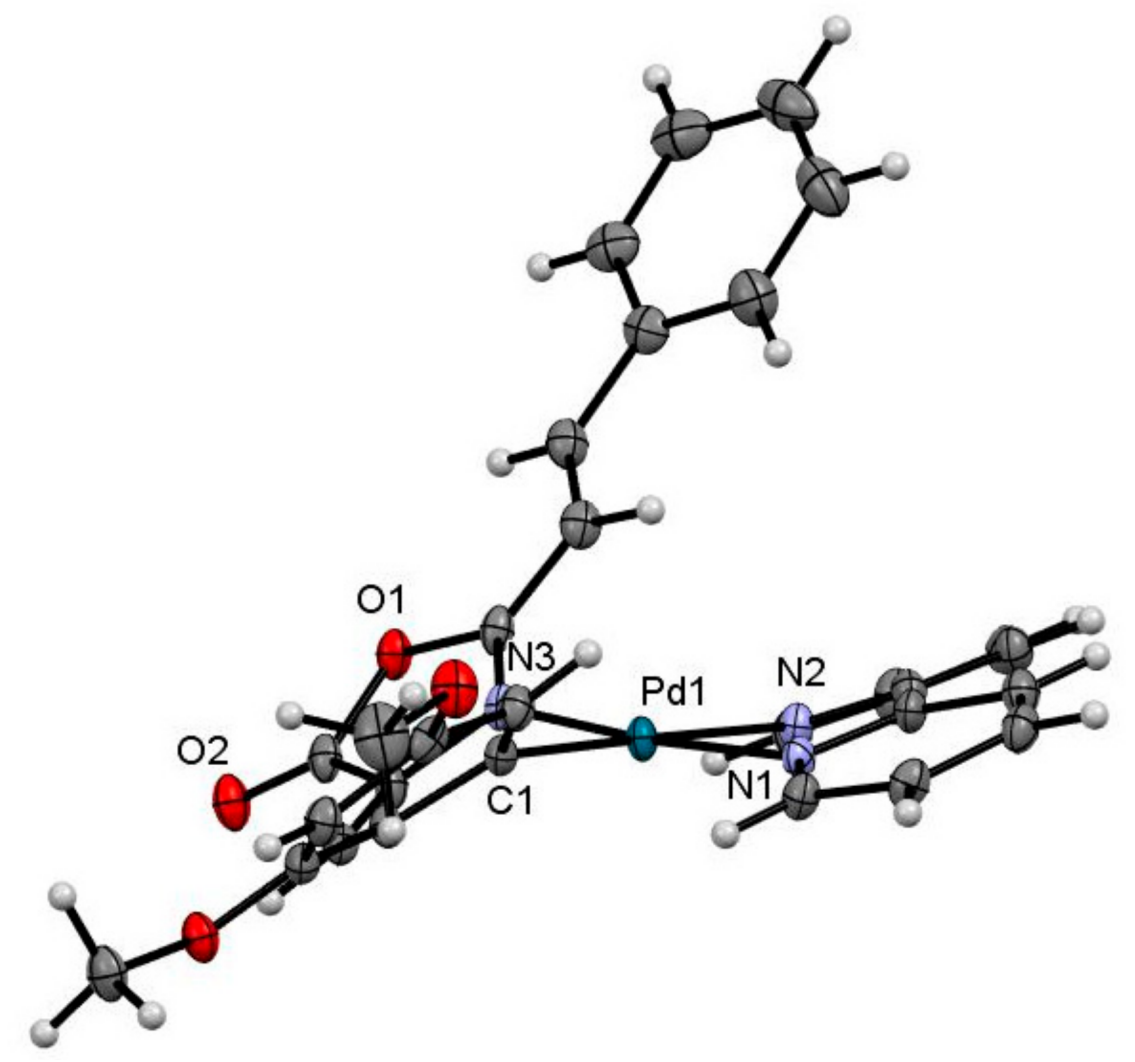
Molecular drawing of complex **7a**, showing the distortion of the oxazolone fragment.

However, beyond the immediate environment of the Pd atom (i.e., the four donor atoms), the complex has a structure that is very distorted (see Figure 6) with respect to the ideal flat geometry expected for highly conjugated ligands such as 2,2-bipyridine and the oxazolone. This distortion is a consequence of the strong intramolecular interactions between the bipy ligand and the oxazolone. This distortion can be envisaged by considering five different planes in the molecule and measuring the dihedral angles between them. Therefore, we define the following best least-squares planes: (1) the molecular plane containing the Pd1–C1–N3–N1–N2 atoms; (2) the 4-arylidene ring C1–C2–C3–C4–C5–C6; (3) the central part of the oxazolone C1–C6–C9–C10–N3; (4) the oxazolone ring and the styryl fragment C10–C20; (5) the bipy ligand N1–C25–C24–C26–N2–C27. Ideally, these five planes should be coplanar and the dihedral angles between them should be zero or close to zero. However, the values found experimentally are as follows: 1–2 = 40.76(3)°; 1–3 = 37.57(3)°; 1–4 = 50.30(3)°; 1–5 = 19.98(4)°; 2–3 = 19.89(3)°; 2–4 = 48.36(3)°; 2–5 = 27.16(4)°; 3–4 = 28.59(3)°; 3–5 = 17.79(4)°; 4–5 = 34.54(4)°. It is clear that the oxazolone shows a marked U-shaped distortion with the aim of minimizing intramolecular repulsions between the styryl fragment CH=CHPh and the pyridine ring N2–C26–C27–C28–C29–C30. In this way, the styryl fragment is on the upper side of the molecular plane (plane 1) while the oxazolone and the orthopalladated rings are in the lower part (Figure 6). The distortion is not particularly strong for the bipy rings.

### 2.3. Fluorescence Studies: Absorption and Excitation–Emission Spectra

The photophysical properties of the starting oxazolones (**1a**, **1b**) and the orthopalladated complexes (**4a**, **4b**, **6a**, **7a**) were measured in CH_2_Cl_2_ solution (concentration 5 × 10^−4^ M) at 25 °C. The two oxazolones **1a** and **1b** showed absorption maxima in the UV region of the spectrum (381–428 nm, Table 2 and SM), a transition corresponding to a π–π* charge transfer [14,15,16]. In the orthopalladated derivatives (**4a**, **4b**, **6a**, **7a**), the maxima were red-shifted to the blue-green region (446–495 nm) with respect to the free oxazolones. This bathochromic shift has already been observed in oxazolones and imidazolones [63].

**Figure 7:**
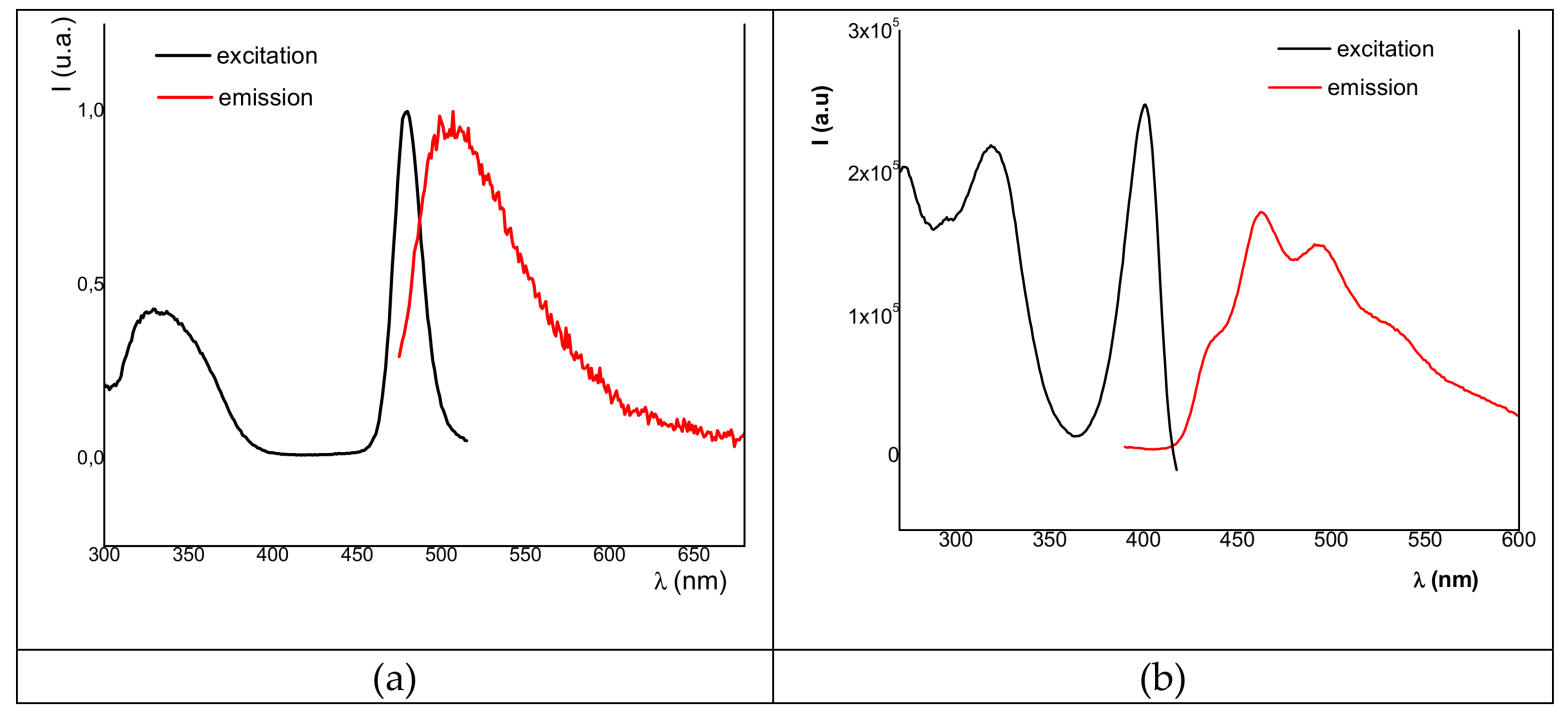
Normalized excitation–emission spectrum of (**a**) **1a**; (**b**) **1b**.

The measurement of the excitation–emission spectra of free oxazolones **1a** and **1b** showed that the excitation maxima were in the range 320–340 nm, as shown in Figure 7 and Table 3. The emission maxima corresponding to this excitation wavelength appeared at 505 and 462 nm, respectively. The two oxazolones showed similar weak emission intensities, as determined from the quantum yield measured for the fluorescence of **1a** (< 1%). This low value is similar to values found in closely related push–pull oxazolones [63]. Therefore, the introduction of the styryl fragment in the 2-position of the oxazolone ring (Kaede chromophore) does not promote significant changes in the position of the absorption maxima, nor in the fluorescence intensity, with respect to those observed in the GFP-related chromophores.

With respect to the fluorescence of complexes **4a**–**8a** and **4b**, two main features were observed (see Table 3): (a) the expected red-shift of the emission maxima (range 525–599 nm) due to the incorporation of the metal into the oxazolone skeleton, as observed in the absorption spectra, and (b) a marked increase in the intensity of the fluorescence emission.

We observed differences in the shape and maxima position between the excitation and absorption spectra. Due to this fact, at this concentration, more diluted solutions (10^−5^ M and 10^−6^ M) of **6a** were studied in detail (see SM). We observed that, upon dilution, the shape and maxima of absorption and excitation spectra fit better, suggesting aggregation effects at higher concentration.

The qualitative comparison of the fluorescence intensity (in counts) shows a general increase of two orders of magnitude from the free oxazolone **1a** (around 2 × 10^4^) to the orthometalated derivatives **4a**–**8a** (around (1–5) × 10^6^). A more rigorous comparison of QY (determined for **6a**, the complex that shows the most intense emission) shows an increase of one order of magnitude, i.e., up to 12%. Therefore, the incorporation of the Pd atom into the oxazolone scaffold produced the expected increase in the fluorescence due to the suppression of non-radiative pathways. However, this increase was not observed in all cases because the intensity of the emission in the neutral complex **4b** was very close to that observed for the free oxazolone **1b**. Although the number of available examples is not large, the obtained data suggest that the presence of the Pd atom as an intramolecular lock, being necessary, is not sufficient, and that additional factors have to be considered. The presence of the Pd atom in the orthometalated oxazolones clearly modifies their electronic properties, such as the push–pull character. It is probable that the enhancement of the emissive behavior demands coordination to the metal, but such coordination should not diminish the D-π-A capability of the oxazolone fragment; rather, if possible, it should increase it. Therefore, these changes in the charge distribution promoted by the incorporation of the Pd could be responsible for the lack of fluorescence amplification from **1b** to **4b**, and even for the small changes in emission intensities observed along the set of complexes **4a**–**8a**. Further studies aiming to establish the contribution of the steric and electronic factors to the increase in the fluorescence are currently being developed in our laboratories.

## 3. Materials and Methods

### 3.1. General Procedures

Solvents were obtained from commercial sources and were used without further purification. All reactions were performed without special precautions against air and moisture. C, H, N and S elemental microanalyses were carried out on a Perkin-Elmer 2400-B Series II Analyser. Electrospray ionization (ESI) mass spectra were recorded using a Bruker Esquire3000 plus™ ion-trap mass spectrometer equipped with a standard ESI source. High-resolution mass spectra-ESI (HRMS-ESI) were recorded using a Bruker MicroToF-Q™ system equipped with an API-ESI source and a Q-ToF mass analyzer, which allows a maximum error in the measurement of 5 ppm. Acetonitrile and methanol were used as solvents. For all types of MS measurements, samples were introduced in a continuous flow of 0.2 mL/min and nitrogen served both as the nebulizer gas and the dry gas. Infrared spectra were recorded on a Spectrum 100 Perkin-Elmer FTIR Spectrophotometer, with a universal attenuated total reflectance (UATR) accessory made from thallium bromide-iodide crystals (KRS-5), which allows the observation of the electromagnetic spectrum in the region between 4000 and 250 cm^−1^. The ^1^H, ^13^C{^1^H} and ^19^F NMR spectra were recorded on a Bruker Avance-300 spectrometer (δ in ppm; *J* in Hz). All experiments were recorded on solution at room temperature using CDCl_3_ as the deuterated solvent. Other conditions are specified for each particular case. The ^1^H and ^13^C{^1^H} spectra were referenced using the residual solvent signal as the internal standard, while ^19^F spectra were referenced to CFCl_3_. All of the experiments were carried out at 298 K (different conditions are indicated as appropriate). Assignment was performed, when necessary, with the help of the following 2D-NMR experiments: ^1^H-^1^H gradient-selected correlation spectroscopy (gCOSY), ^1^H-^13^C heteronuclear single quantum coherence (*HSQC*), ^1^H-^13^C heteronuclear multiple bond correlation (*HMBC*) and ^1^H-^1^H nuclear Overhauser enhancement spectroscopy (*NOESY*) experiments. Diffusion experiments (DOSY) of **2b** were carried out on CDCl3 solution of an approximate 4 mM concentration at controlled temperature (300 K). The P30 (little delta) and D20 (big delta) values were optimized to achieve an almost complete attenuation of the signal for a value of the gradient (gpz6) of 90% of full power. Absorption spectra were measured on a Thermo Scientific Evolution 600BB spectrophotometer. The steady-state excitation–emission spectra were measured on a Jobin-Yvon Horiba Fluorolog FL-3-11 spectrofluorimeter. All measurements were carried out at room temperature on solutions of 5 × 10^−4^ M concentration using quartz cuvettes of 1 cm path length. The measurement of the quantum yield values (Φ) of **1a** and **6a** was carried out using the absolute method on a Quantaurus-QY C11347 spectrometer. Two different CH_2_Cl_2_ solutions of each compound (5 × 10^−4^ M) were measured in order to check data reproducibility. In addition, one solution of each compound was deoxygenated by passing argon through it, and the value of the QY was redetermined to check the influence of the O_2_ in the intensity of the luminescence. The oxazolones **1a** and **1b** were prepared using the Erlenmeyer–Plöchl method, by reaction of *N*-cinnamoylglycine with the corresponding aldehyde [74,75,76,77,78,79,80]. The *N*-cinnamoylglycine was prepared by the Schotten–Baumann method [81].

### 3.2. X-ray Crystallography

Single crystals of **1a** and **7a** of suitable quality for X-ray diffraction measurements were grown by slow diffusion of *n*-pentane into CH_2_Cl_2_ solutions of the crude product at –18 °C for one week. One selected single crystal of each compound was mounted at the end of a quartz fiber in a random orientation, covered with perfluorinated oil (magic oil) and placed under a cold stream of N_2_ gas. Crystallographic measurements were carried out at 100 K on a Bruker Smart APEX CCD diffractometer, using graphite-monochromated Mo Kα radiation (λ = 0.71073 Å). A hemisphere of data was collected in each case based on ω-scan or ϕ-scan runs. The diffraction frames were integrated using the program SAINT [86] and the integrated intensities were corrected for absorption with SADABS [87]. The structures were solved by direct methods with SHELXT-2014 [88]. All non-hydrogen atoms were refined with anisotropic displacement parameters. The hydrogen atoms were placed at idealized positions and treated as riding atoms. Each hydrogen atom was assigned an isotropic displacement parameter equal to 1.2–1.5 times the equivalent isotropic displacement parameter of its parent atom. For structure solving and refinement, the SHELXL-2016 [89] program in the WINGX Package was used [90]. The structures were refined to F_o_^2^, and all reflections were used in the least-squares calculations. The structure of **1a** could be solved, but the quality of the data was poor and the analysis of bond distances and angles could not be performed. As a consequence, this structure was used only as a connectivity scheme. CCDC-2035173 (**7a**) contains the supplementary crystallographic data. These data can be obtained free of charge from the Cambridge Crystallographic Data Centre via www.ccdc.cam.ac.uk/data_request/cif. 

### 3.3. Synthesis and Characterization of Oxazolones **1a** and **1b**

#### 3.3.1. Synthesis of 4-((*Z*)-2,4-Dimethoxybenzylidene)-2-((*E*)-Styryl)-5(4*H*)-Oxazolone (**1a**)

Sodium acetate (399.7 mg, 4.9 mmol) and 2,4-dimethoxybenzaldehyde (850.3 mg, 5.1 mmol) were added to a solution of *N*-cinnamoylglycine (1000.0 mg, 4.9 mmol) in acetic anhydride (10 mL). The suspension was heated under reflux (110 °C) for 3 h and then allowed to cool to room temperature. The solid formed upon cooling was treated with distilled water (30 mL) to give **1a** as a deep orange solid, which was filtered off, washed with water (5 mL) and cold ethanol (10 mL) and dried under vacuum. Obtained: 509.1 mg (31% yield). HRMS (ESI^+^) [*m*/*z*]: calculated for C_20_H_18_NO_4_ 336.1236 [M + H]^+^; found 336.1237. ^1^H NMR (CDCl_3_, 300.13 MHz): δ = 8.75 (d, *J* = 8.7 Hz, 1H, H_6_, C_6_H_3_), 7.77 (s, 1H, =CH_vinyl_), 7.62 (d, *J* = 16.2 Hz, 1H, =CH_olef_), 7.57 (m, 2H, H_o_, C_6_H_5_), 7.44–7.40 (m, 3H, H_m_, H_p_, C_6_H_5_), 6.79 (d, *J* = 16.2 Hz, 1H, =CH_olef_), 6.63 (dd, *J* = 8.7, 1.8 Hz, 1H, H_5_, C_6_H_3_), 6.43 (d, *J* = 2.1 Hz, 1H, H_3_, C_6_H_3_), 3.88 (s, 6H, OCH_3_). ^13^C{^1^H} NMR (CDCl_3_, 75.47 MHz): δ = 167.82 (s, C=O), 164.10 (s, C–O, C_6_H_3_), 161.78 (s, C–O, C_6_H_3_), 161.06 (s, C=N), 142.31 (s, =CH, C_olef_), 134.85 (s, =C), 134.35 (s, CH, C_6_, C_6_H_3_), 130.49 (s, C_i_, C_6_H_5_), 130.36 (s, CH, C_p_, C_6_H_5_), 129.04 (s, CH, C_o_, C_6_H_5_), 127.97 (s, CH, C_m_, C_6_H_5_), 125.75 (s, =CH, C_vinyl_), 116.21 (s, C_1_, C_6_H_3_), 113.69 (s, =CH, C_olef_), 106.32 (s, CH, C_5_, C_6_H_3_), 97.68 (s, CH, C_3_, C_6_H_3_), 55.66 (s, OCH_3_), 55.55 (s, OCH_3_).

#### 3.3.2. Synthesis of 4-((*Z*)-2,4-Difluorobenzylidene)-2-((*E*)-Styryl)-5(4*H*)-Oxazolone (**1b**)

The oxazolone **1b** was prepared following the same experimental method as reported for **1a** but using the corresponding aldehyde. Therefore, *N*-cinnamoylglycine (1000.0 mg, 4.9 mmol) was reacted with 2,4-difluorobenzaldehyde (0.56 mL, 5.1 mmol) and sodium acetate (399.7 mg, 4.9 mmol) in 10 mL of acetic anhydride under reflux (110 °C) for 3 h to give **1b** as a yellow solid. Obtained: 426.6 mg (28% yield). HRMS (ESI^+^) [*m*/*z*]: calculated for C_18_H_12_F_2_NO_2_ 312.0836 [M + H]^+^; found 312.0832. ^1^H NMR (300.13 MHz, CD_2_Cl_2_): δ = 8.89 (td, *J* = 8.7, 6.6 Hz, 1H, H_6_, C_6_H_3_F_2_), 7.80 (d, *J* = 16.2 Hz, 1H, =CH_olef_), 7.66 (m, 2H, H_o_, C_6_H_5_), 7.49–7.47 (m, 3H, H_m_, H_p_, C_6_H_5_), 7.41 (s, 1H,=CH_vinyl_), 7.08 (td, *J* = 8.7, 2.4 Hz, 1H, H_5_, C_6_H_3_), 6.95 (ddd, *J* = 11.4, 9.0, 2.7 Hz, 1H, H_3_, C_6_H_3_), 6.87 (d, *J* = 16.2 Hz, 1H, =CH_olef_). ^19^F NMR (282.40 MHz, CD_2_Cl_2_): δ = –103.89 (quint, 1F, F-4, *J* = 8.5 Hz), –110.25 (quart, 1F, F-2, *J* = 8.5 Hz). ^13^C{^1^H} NMR (CD_2_Cl_2_, 75.47 MHz): δ = 166.62 (s, C=O), 164.49 (dd, ^1^*J*_CF_ = 255.8 Hz, ^3^*J*_CF_ = 12.1 Hz, C_2_–F, C_6_H_3_F_2_), 164.21 (s, C=N), 162.34 (dd, ^1^*J*_CF_ = 258.9 Hz, ^3^*J*_CF_ = 12.1 Hz, C_4_-F, C_6_H_3_F_2_), 144.40 (s, =CH, C_olef_), 134.49 (s, C, C_i,_ C_6_H_5_), 134.33 (d, ^4^*J*_CF_ = 4.5 Hz, =C), 133.96 (dd, ^3^*J*_CF_ = 9.8, ^3^*J*_CF_ = 3.0 Hz, C_6_-H, C_6_H_3_F_2_), 130.92 (s, CH, C_p_, C_6_H_5_), 129.09 (s, CH, C_m_, C_6_H_5_), 128.22 (s, CH, C_o_, C_6_H_5_), 120.07 (dd, ^3^*J*_CF_ = 6.8 Hz, ^5^*J*_CF_ = 2.3 Hz, =CH, C_vinyl_), 118.49 (dd, ^3^*J*_CF_ = 11.3 Hz, ^4^*J*_CF_ = 3.8 Hz, C_1_, C_6_H_3_F_2_), 113.10 (s, CH, =CH, C_olef_), 112.33 (dd, ^2^*J*_CF_ = 21.1 Hz, ^3^*J*_CF_ = 3.8 Hz, C_5_–H, C_6_H_3_F_2_), 103.99 (t, ^2^*J*_CF_ = 25.6 Hz, C_3_–H, C_6_H_3_F_2_).

### 3.4. Synthesis and Characterization of the Orthopalladated Dimers with Trifluoroacetate Bridges **2a,** **2b**

#### 3.4.1. Synthesis of Orthopalladated **2a**

Pd(OAc)_2_ (137.2 mg, 0.6 mmol) was added to a solution of the oxazolone **1a** (205.0 mg, 0.6 mmol) in trifluoroacetic acid (5 mL). The resulting suspension was stirred at room temperature for 40 min and distilled water (10 mL) was added. The resulting precipitate was filtered off, washed with distilled water (3 × 10 mL) until the characteristic smell of trifluoroacetic acid disappeared and dried under vacuum. Compound **2a** was obtained as a red solid. Obtained: 316.2 mg (94% yield). **HRMS** (ESI^+^) [*m*/*z*]: calculated for C_40_H_33_N_2_O_9_Pd_2_ 897.0256 [M–2CF_3_COO + OH]^+^; found: 896.9991. **^1^H NMR** (CDCl_3_, 300.13 MHz): δ = 7.95 (s, 1H, H_vinyl_), 7.56 (m, 2H, H_o,_ C_6_H_5_), 7.44–7.39 (m, 4H, H_olef_, H_m_+H_para_, C_6_H_5_), 7.08 (d, *J* = 15.9 Hz, 1H, H_olef_), 6.23 (d, *J* = 1.8 Hz, 1H, H_3_, C_6_H_2_), 6.17 (d, *J* = 2.1 Hz, 1H, H_5_, C_6_H_2_), 3.94 (s, 3H, OCH_3_), 3.53 (s, 3H, OCH_3_). **^13^C{^1^H} NMR** (CDCl_3_, 75.47 MHz): δ = 163.20 (s, C=N), 161.58 (s, C–O, C_6_H_2_), 160.46 (s, C=O), 158.82 (s, C–O, C_6_H_2_), 145.95 (s, =CH, C_olef_), 139.08 (s, =C), 134.01 (s, C_i_, C_6_H_5_), 131.57 (s, CH, C_p_, C_6_H_5_), 130.87 (s, =CH, C_vinyl_), 129.04 (s, CH, C_o,_ C_6_H_5_), 128.81 (s, CH, C_m,_ C_6_H_5_), 118.73 (s, C, C_6_H_2_), 115.36 (q, CF_3_, ^1^*J*_CF_ = 287.5 Hz), 113.68 (s, C–Pd, C_6_H_2_), 110.69 (s, =CH, C_olef_), 108.97 (s, CH, C_6_H_2_), 96.66 (s, CH, C_6_H_2_), 55.86 (s, OCH_3_), 55.21 (s, OCH_3_). The signal due to the presence of the carboxylate carbon from the bridging CF_3_COO ligand was not found, despite the use of long accumulation times, probably due to the low solubility of this compound. **^19^F NMR** (376 MHz, CDCl_3_): δ = −74.49 (s, CF_3_). **IR** (ν, cm^−1^): 1790 (C=O), 1662,1571 (O-C=N), 1642 (CF_3_COO bridging).

#### 3.4.2. Reaction of Oxazolone **1b** with Pd(OAc)_2_: Dinuclear and Trinuclear Derivatives (mixture **2b**)

The oxazolone **1b** (200.0 mg, 0.6 mmol) was reacted with Pd(OAc)_2_ (143.7 mg, 0.6 mmol) under reflux in trifluoroacetic acid (8 mL, 75 °C) for 4 h. After the reaction time, the mixture was allowed to cool to room temperature and water (20 mL) was added. Further stirring produced a yellowish-orange precipitate (**2b**). The solid was filtered off, washed with water (10 mL) and Et_2_O (10 mL) and dried under vacuum. Obtained: 312.8 mg. Product **2b** was characterized by ^1^H, ^1^H{^19^F} and ^19^F NMR spectra as a mixture of four different compounds: two dinuclear (*anti* and *syn*) and two trinuclear (*anti* and *syn*). Only the major isomer (*anti*) of the dinuclear derivatives could be fully characterized by ^13^C NMR. **^1^H{^19^F} NMR** (300.13 MHz, CD_2_Cl_2_): δ = 7.9–7.4 (m, =CH_vinyl_, =CH_olef_, C_6_H_5_, all isomers), 7.25 (d, *J* = 15.9 Hz, =CH_olef_), 7.23 (d, *J* = 15.9 Hz, =CH_olef_), 7.21 (d, *J* = 15.9 Hz, =CH_olef_), 7.09 (d, *J* = 15.9 Hz, =CH_olef_, dinuclear anti isomer, major isomer), 6.76, 6.68 (AB spin system, *J* = 2.4 Hz, C_6_H_2_F_2_ dinuclear *anti* isomer, major isomer), 6.71, 6.68 (AB spin system, *J* = 2.4 Hz, C_6_H_2_F_2_), 6.56, 6.47 (AB spin system, *J* = 2.4 Hz, C_6_H_2_F_2_). **^19^F NMR** (282.4 MHz, CD_2_Cl_2_): δ = −75.07 (s, 3F, CF_3_), −101.02 (q, *J* = 9 Hz, 1F, C_6_H_2_F_2_), −108.68 (d, *J* = 6 Hz, 1F, C_6_H_2_F_2_) (dinuclear *anti* isomer, major isomer); −74.68, −74.69 (2s, 2CF_3_), −101.24 (q, *J* = 9 Hz, C_6_H_2_F_2_), −109.43 (d, *J* = 6 Hz, C_6_H_2_F_2_); −74.20, −74.43 (2s, 2CF_3_), −102.28 (q, *J* = 9 Hz, C_6_H_2_F_2_), −110.28, −110.49 (m, C_6_H_2_F_2_); −74.44, −74.45 (2s, 2CF_3_), −102.44 (q, *J* = 9 Hz, C_6_H_2_F_2_), −110.28 to −110.49 (d, *J* = 6 Hz, C_6_H_2_F_2_) (dinuclear and trinuclear minor isomers). **^13^C{^1^H} NMR** (75.47 MHz, CD_2_Cl_2_): δ = 166.72 (s, C=N), 166.43 (s, C=O), 166.22 (q, ^2^*J* = 40.7 Hz, CO_2_CF_3_), 160.67 (dd, ^1^*J* = 235.02 Hz, ^3^*J* = 12.58 Hz, C_2_–F, C_6_H_2_F_2_), 159.36 (dd, ^1^*J* = 265.11 Hz, ^3^*J* = 13.32 Hz, C_4_-F, C_6_H_2_F_2_), 150.00 (s, =CH_olef_), 136.22 (br, =C), 136.09 (br, Pd–C_6_, C_6_H_2_F_2_), 133.49 (s, C_i_, C_6_H_5_), 132.68 (s, C_p_, CH, C_6_H_5_), 129.44 (s, C_m_, CH, C_6_H_5_), 129.18 (s, C_o_, CH, C_6_H_5_), 126.00 (d, *J* = 6.0 Hz, =CH_vinyl_), 116.25 (dd, ^2^*J*_CF_ = 21.9 Hz, ^4^*J*_CF_ = 3.0 Hz, C_5_H, C_6_H_2_F_2_), 115.33 (br, C_1_, C_6_H_2_F_2_), 109.66 (s, =CH, C_olef_), 101.63 (t, ^2^*J*_CF_ = 25.6 Hz, C_3_H, C_6_H_2_). The signal due to the CF_3_ carbon was not observed. **IR** (ν, cm^−1^): 1789 (C=O), 1651, 1572(O-C=N), 1605 (CF_3_COO bridging).

### 3.5. Synthesis and Characterization of the Orthopalladated Dimers with Chloride Bridges **3a,** **3b**

#### 3.5.1. Synthesis of Orthopalladated **3a**

An excess of anhydrous LiCl (30.6 mg, 0.7 mmol) was added to a solution of **2a** (200.0 mg, 0.2 mmol) in methanol (15 mL), and the resulting mixture was stirred at room temperature for 2 h. During this time, a deep red solid precipitated. The solid (**3a**) was filtered off, washed with methanol (10 mL) and Et_2_O (20 mL) and dried under vacuum. Obtained: 126.5 mg (74% yield). Compound **3a** was insoluble in the usual NMR solvents, thus precluding its routine characterization in solution even in the presence of pyridine-*d*_5_. Elemental analysis calculated for C_40_H_32_Cl_2_N_2_O_8_Pd_2_: C, 50.44; H, 3.39; N, 2.94; found: C, 50.13; H, 3.44; N, 3.08. **IR** (ν, cm^−1^): 1775 (C=O), 1637, 1560 (O–C=N), 314 (Pd–Cl).

#### 3.5.2. Synthesis of Orthopalladated **3b**

Complex **3b** was prepared following the same experimental procedure as for **3a**. Compound **2b** (200.0 mg) was reacted with LiCl (32.2 mg, 0.8 mmol) in methanol (8 mL) at room temperature to give **3b** as an orange solid. Obtained: 138.0 mg, (79% yield). Compound **3a** was insoluble in the usual NMR solvents, thus precluding its routine characterization in solution even in the presence of pyridine-d_5_. Elemental analysis calculated for C_36_H_20_Cl_2_F_4_N_2_O_4_Pd_2_: C, 47.82; H, 2.23; N, 3.10; found: C, 47.47; H, 2.44; N, 2.99. **IR** (ν, cm^−1^): 1820, 1798 (C=O), 1654, 1591 (O–C=N), 318 (Pd–Cl).

### 3.6. Synthesis and Characterization of Orthopalladated Complexes with Acetylacetonate **4a** and **4b**

#### 3.6.1. Synthesis of Orthopalladated **4a**

Tlacac (63.7 mg, 0.2 mmol) was added to a stirred suspension of **3a** (100.0 mg, 0.1 mmol) in CH_2_Cl_2_ (10 mL). The appearance of the suspension changed in a few minutes and a red solution with a gray solid in suspension was obtained. The mixture was stirred at room temperature for 20 min and then filtered over a Celite pad. The Celite was washed with CH_2_Cl_2_ (10 mL). The clear red solution was evaporated to dryness and the solid residue was treated with cold *n*-pentane (20 mL). The resulting orange solid was filtered off, washed with *n*-pentane (2 × 5 mL), dried under vacuum and characterized as **4a**. Obtained: 100.0 mg (88% yield). **HRMS** (ESI^+^) [*m*/*z*]: calculated for C_21_H_20_NO_5_Pd 472.0376 [M–acac + CH_3_OH]^+^; found 472.0396. **^1^H NMR** (300.13 MHz, CDCl_3_): δ = 8.13 (s, 1H, =CH_vinyl_), 7.67 (d, *J* = 16 Hz, 1H, =CH_olef_), 7.60–7.52 (m, 3H, H_o_, C_6_H_5_, =CH_olef_), 7.44–7.42 (m, 3H, H_m_, H_p_, C_6_H_5_), 7.04 (d, *J* = 2.4 Hz, 1H, C_6_H_2_, H_5_), 6.18 (d, *J* = 2.4 Hz, 1H, C_6_H_2_, H_3_), 5.45 (s, 1H, CH, acac), 3.90 (s, 3H, OCH_3_), 3.85 (s, 3H, OCH_3_), 2.08 (s, 3H, CH_3_, acac), 1.94 (s, 3H, CH_3_, acac). **^13^C{^1^H} NMR** (75.47 MHz, CDCl_3_): δ = 187.64 (s, C–O, acac), 186.80 (s, C–O, acac), 162.86 (s, C_2_–O, C_6_H_2_), 162.41 (s, C=N), 162.23 (s, C_4_–O, C_6_H_2_), 160.14 (s, C=O), 150.10 (s, =C), 143.75 (s, =CH, C_olef_), 135.02 (s, C_i_, C_6_H_5_), 133.17 (s, =CH, C_vinyl_), 130.99 (s, CH, C_p_, C_6_H_5_), 129.25 (s, CH, C_o_, C_6_H_5_), 128.47 (s, C_m_, C_6_H_5_), 119.58 (s, C_1_, C_6_H_2_), 116.35 (s, C_6_–Pd, C_6_H_2_), 113.13 (s, =CH, C_olef_), 111.07 (s, C_5_H, C_6_H_2_), 100.34 (s, CH, acac), 95.80 (s, C_3_H, C_6_H_2_), 55.83 (s, OCH_3_), 55.57 (s, OCH_3_), 27.87 (s, CH_3_, acac), 27.76 (s, CH_3_, acac).

#### 3.6.2. Synthesis of Orthopalladated **4b**

Complex **4b** was obtained following the same experimental procedure as described for **4a**. Compound **3b** (100.0 mg, 0.1 mmol) was reacted with Tlacac (70.5 mg, 0.2 mmol) in CH_2_Cl_2_ (10 mL) to give **4b** as an orange solid. Obtained: 90.2 mg (79% yield). **HRMS** (ESI^+^) [*m*/*z*]: calculated for C_19_H_14_F_2_NO_3_Pd 447.9977 [M–acac + CH_3_OH]^+^; found 447.9972. **^1^H NMR** (300.13 MHz, CDCl_3_): δ = 7.83 (s, 1H, =CH_vinyl_), 7.76 (d, *J* = 16.2 Hz, 1H, =CH_olef_), 7.61–7.55 (m, 3H, H_o_, C_6_H_5_, =CH_olef_), 7.46–7.40 (m, 3H, H_m_, H_p_, C_6_H_5_), 7.38 (dm, *J* = 9.6 Hz, 1H, C_6_H_2_F_2_, H_5_), 6.61 (ddd, *J* = 10.5, 8.4, 2.4 Hz, C_6_H_2_F_2_, H_3_), 5.47 (s, 1H, CH, acac), 2.12, 1.94 (2s, 2CH_3_, acac). **^19^F{^1^H} NMR** (282.4 MHz, CDCl_3_): δ = −101.99 (d, *J* = 8.5 Hz, 1F), −111.07 (d, *J* = 11.3 Hz, 1F). **^13^C{^1^H} NMR** (75.47 MHz, CDCl_3_): δ = 187.49 (s, C–O, acac), 187.21 (s, C–O, acac), 165.53 (s, C=N), 161.32 (s, C=O), 146.58 (s, =CH, C_olef_), 134.57 (s, C_i_, C_6_H_5_) 131.79 (s, C_p_, C_6_H_5_), 129.40 (s, C_o_, C_6_H_5_), 129.04 (d, ^3^*J*_CF_ = 6.0 Hz, =CH, C_vinyl_), 128.90 (s, C_m_, C_6_H_5_), 117.07 (d, ^2^*J*_CF_ = 16.6 Hz, C_5_H, C_6_H_2_F_2_), 112.68 (s, =CH, C_olef_), 100.62 (s, CH, acac), 100.61 (d, ^2^*J*_CF_ = 52.82 Hz, C_3_H, C_6_H_2_), 27.76 (s, CH_3_, acac), 27.71 (s, CH_3_, acac). Signals due to the =C carbon of the oxazolone ring and the quaternary C atoms of the C_6_H_2_F_2_ ring (C_1_, C_2_–F, C_4_–F and C_6_–Pd) were not observed due to the low solubility of this compound.

### 3.7. Synthesis and Characterization of Orthopalladated Complex with 8-Hydroxyquinolinate **5a**

8-Hydroxyquinoline (30.5 mg, 0.2 mmol) and Ag_2_CO_3_ (57.9 mg, 0.2 mmol) were added to a suspension of **3a** (100.0 mg, 0.1 mmol) in a mixture of CH_2_Cl_2_ and acetone (8:2, 20 mL), protected from light. The mixture was stirred at room temperature for 16 h and filtered through a pad of Celite. The Celite was washed with CH_2_Cl_2_ (2 × 10 mL) and the clear red solution was evaporated to dryness. The residue was treated with cold Et_2_O (25 mL) and stirred. The resulting suspended red solid was filtered off, washed with Et_2_O (10 mL), dried under vacuum and identified as **5a**. Obtained: 111.2 mg (91% yield). **HRMS** (ESI^+^) [*m*/*z*]: calculated for C_29_H_23_N_2_O_5_Pd 585.0642 [M + H]^+^; found 585.0656. **^1^H NMR** (300.13 MHz, CD_2_Cl_2_): δ = 8.62 (d, *J* = 5.1 Hz, 1H, H_1′_, C_9_H_6_NO), 8.54 (d, *J* = 8.1 Hz, 1H, H_3′_, C_9_H_6_NO), 8.02 (s, 1H, =CH_vinyl_), 7.78 (dd, *J* = 7.5, 5.1 Hz, 1H, H_2′_, C_9_H_6_NO), 7.47–7.36 (m, 6H, H_7′_, H_6′_, H_5’_, C_9_H_6_NO, =CH_olef_, H_o_ C_6_H_5_), 7.16–7.04 (m, 3H, H_m_, H_p_, C_6_H_5_), 7.06 (d, *J* = 16 Hz, 1H, =CH_olef_), 6.45, 6.42 (AB spin system, *J* = 1.8 Hz, 2H, C_6_H_2_, H_3_, H_5_), 4.11 (s, 3H, OCH_3_), 3.92 (s, 3H, OCH_3_). **^13^C{^1^H} NMR** (75.47 MHz, CD_2_Cl_2_): δ = 149.35 (s, CH, C_1′_, C_9_H_6_NO), 140.73 (s, CH, C_3′_, C_9_H_6_NO), 132.28 (s, =CH, C_vinyl_), 132.14 (s, =CH, C_olef_), 130.67 (s, C_p_, C_6_H_5_), 129.56 (2C overlapped, CH, C_6′_, C_7′_, C_9_H_6_NO), 129.05 (s, C_o_, C_6_H_5_), 128.61 (s, =CH, C_olef_), 128.33 (s, CH, C_5′_, C_9_H_6_NO), 128.14 (s, C_m_, C_6_H_5_), 123.00 (s, CH, C_2′_, C_9_H_6_NO), 118.83 (s, CH, C_6_H_2_), 95.32 (s, CH, C_6_H_2_), 56.48 (s, OCH_3_), 55.98 (s, OCH_3._). The low solubility of this compound, even in CD_2_Cl_2_, only allowed the CH nuclei to be detected properly, thus precluding the observation of signals due to quaternary C nuclei even in HMBC correlations.

### 3.8. Synthesis and Characterization of Orthopalladated Complex with 8-Aminoquinoline **6a**

AgClO_4_ (45.7 mg, 0.2 mmol) was added to a suspension of **3a** (100 mg, 0.1 mmol) in a mixture of CH_2_Cl_2_ and acetone (9:1, 10 mL), protected from light. The resulting suspension was stirred at room temperature for 1 h and filtered over a Celite pad in order to remove the AgCl. The Celite pad was washed with additional CH_2_Cl_2_ (10 mL). The clear filtered solution of the solvate was treated with 8-aminoquinoline (31.7 mg, 0.2 mmol) and stirring was maintained at room temperature for 1 h. The resulting solution was evaporated to dryness and the residue was treated with Et_2_O (20 mL). Upon further stirring, a red solid (**6a**) precipitated, and this was filtered off, washed with ether (20 mL) and dried under vacuum. Obtained: 136.9 mg (97% yield). **HRMS** (ESI^+^) [*m*/*z*]: calculated for C_29_H_24_N_3_O_4_Pd 584.0802 [M–ClO_4_]^+^; found 584.0793. **^1^H NMR** (300.13 MHz, CD_2_Cl_2_): δ = 8.54 (dd, *J* = 5.1, 1.2 Hz, 1H, H_1′_, C_9_H_8_N_2_), 8.46 (dd, *J* = 8.4, 1.5 Hz, 1H, H_3′_, C_9_H_8_N_2_), 8.20 (d, *J* = 7.2 Hz, 1H, H_7′_, C_9_H_8_N_2_), 8.11 (s, 1H, =CH_vinyl_), 8.01 (d, *J* = 16 Hz, 1H, =CH_olef_), 7.95 (d, *J* = 8 Hz, 1H, H_5′_, C_9_H_8_N_2_), 7.78 (t, 1H, H_6′_, C_9_H_8_N_2_), 7.57 (m, 2H, H_o_, C_6_H_5_), 7.48 (dd, *J* = 8.4, 5.1 Hz, 1H, H_2′_, C_9_H_8_N_2_), 7.46–7.32 (m, 3H, H_m_, H_p_, C_6_H_5_), 7.11 (d, *J* = 16.2 Hz, 1H, =CH_olef_), 6.83 (d, *J* = 1.8 Hz, 1H, H_5_, C_6_H_2_), 6.31 (d, *J* = 2.1 Hz, 1H, H_3_, C_6_H_2_), 4.08 (s, 3H, OCH_3_), 3.93 (s, 3H, OCH_3_). **^13^C{^1^H} NMR** (75.47 MHz, CD_2_Cl_2_): δ = 164.07 (s, C_2_–O, C_6_H_2_), 161.71 (s, C_4_–O, C_6_H_2_), 161.59 (s, C=N), 161.09 (s, C=O), 149.78 (s, CH, C_1′_, C_9_H_8_N_2_), 148.57 (s, =C), 146.83 (s, =CH, C_olef_), 146.23 (s, C, C_4′_, C_9_H_8_N_2_), 139.64 (s, CH, C_3′_, C_9_H_8_N_2_), 137.21 (s, C, C_8′_, C_9_H_8_N_2_), 134.40 (s, =CH, C_vinyl_), 133.76 (s, C_1_, C_6_H_2_), 131.95 (s, CH, C_p_, C_6_H_5_), 129.98 (s, C_i_, C_6_H_5_), 129.28 (s, C_m_, C_6_H_5_), 129.04 (s, CH, C_7′_, C_9_H_8_N_2_), 128.86 (s, C_o_, C_6_H_5_), 128.53 (s, CH, C_5′_, C_9_H_8_N_2_), 127.66 (s, CH, C_6′_, C_9_H_8_N_2_), 122.16 (s, CH, C_2′_, C_9_H_8_N_2_), 119.06 (s, C, C_9′_, C_9_H_8_N_2_), 117.06 (s, C_6_-Pd, C_6_H_2_), 113.78 (s, C_5_H, C_6_H_2_), 110.69 (s, =CH, C_olef_), 96.25 (s, C_3_H, C_6_H_2_), 56.57 (s, OCH_3_), 55.93 (s, OCH_3_).

### 3.9. Synthesis and Characterization of Orthopalladated Complex with 2,2’-Bipyridine **7a**

Compound **7a** was prepared following the same experimental method as for **6a**. Compound **3a** (100.0 mg, 0.1 mmol) was reacted with AgClO_4_ (45.7 mg, 0.2 mmol) and 2,2’-bipyridine (34.4 mg, 0.2 mmol) in 10 mL of a mixture of CH_2_Cl_2_/acetone (9/1) to give **7a** as a red solid. Obtained: 132.1 mg (93% yield). **HRMS** (ESI^+^) [*m*/*z*]: calculated for C_30_H_24_N_3_O_4_Pd 596.0802 [M–ClO_4_]^+^; found 596.0795. **^1^H NMR** (300.13 MHz, CDCl_3_): δ = 8.62 (br, 2H, H_2__′_, H_11’_, bipy), 8.30 (br, 2H, H_5′_, H_8’_, bipy), 8.09 (s, 1H, =CH_vinyl_), 8.06 (br, 2H, H_4′_, H_9’_, bipy), 7.87 (d, *J* = 15.9 Hz, 1H, =CH_olef_), 7.57 (br, 2H, H_3′_, H_10’_, bipy), 7.44-7.42 (m, 5H, H_o_, H_m_, H_p_, C_6_H_5_), 6.70 (d, *J* = 15.9 Hz, 1H, =CH_olef_), 6.60 (d, *J* = 1.8 Hz, 1H, C_6_H_2_, H_5_), 6.29 (d, *J* = 1.8 Hz, 1H, C_6_H_2_, H_3_), 3.93 (s, 3H, OCH_3_), 3.88 (s, 3H, OCH_3_). **^13^C{^1^H} NMR** (75.47 MHz, CDCl_3_): δ = 164.16 (s, C_2_–O, C_6_H_2_), 162.96 (s, C=N), 160.68 (s, C_4_–O, C_6_H_2_), 160.30 (s, C=O), 158.39 (2C overlapped, C, C_6′_, C_7__’_, bipy), 151.86 (s, =C), 151.01 (2C overlapped, CH, C_2’_, C_11__′_, bipy), 147.39 (s, =CH, C_olef_), 141.71 (s, CH, C_9′_, bipy), 139.48 (s, CH, C_4′_, bipy), 135.70 (s, =CH, C_vinyl_), 133.42 (s, C_i_, C_6_H_5_), 132.64 (s, C_p_, C_6_H_5_) 129.73 (s, C_o,_ C_6_H_5_), 129.14 (s, C_m_, C_6_H_5_), 125.99 (s, CH, C_8′_, bipy), 124.75 (2C overlapped, CH, C_3′_, C_10__’_, bipy), 122.92 (s, CH, C_5′_, bipy), 120.43 (s, C_6_-Pd, C_6_H_2_), 118.58 (s, C, C_1_, C_6_H_2_), 115.89 (s, CH, C_5_, C_6_H_2_), 110.93 (s, =CH, C_olef_), 95.90 (s, CH, C_3_, C_6_H_2_), 56.34 (s, OCH_3_), 56.14 (s, OCH_3_).

### 3.10. Synthesis and Characterization of Orthopalladated Complex with 1,10-Phenanthroline **8a**

Compound **8a** was prepared following the same experimental method as for **6a**. Compound **3a** (100.0 mg, 0.1 mmol) was reacted with AgClO_4_ (45.7 mg, 0.2 mmol) and 1,10-phenanthroline mono-hydrate (43.5 mg, 0.2 mmol) in 10 mL of a mixture of CH_2_Cl_2_/acetone (9/1) to give **8a** as a deep red solid. Obtained: 147.8 mg (95% yield). **HRMS** (ESI^+^) [*m*/*z*]: calculated for C_32_H_24_N_3_O_4_Pd 620.0802 [M–ClO_4_]^+^; found 620.0834. **^1^H NMR** (300.13 MHz, CD_2_Cl_2_): δ = 8.77–8.75 (m, 2H, H_2′_, H_13′_, phen), 8.67 (d, *J* = 7.2 Hz, 1H, H_4′_, phen), 8.42 (d, *J* = 7.8 Hz, 1H, H_11′_, phen), 8.16 (s, 2H, H_7′_, H_8′_, phen), 8.14 (s, 1H, =CH_vinyl_), 8.01–7.92 (m, 1H, =CH_olef_), 7.78–7.74 (m, 2H, H_3′_, H_12′_, phen), 7.55–7.36 (m, 5H, H_o_, H_m_, H_p_, C_6_H_5_), 6.90 (d, *J* = 15.9 Hz, 1H, =CH_olef_), 6.75 (d, *J* = 1.8 Hz, 1H, H_5_, C_6_H_2_), 6.39 (d, *J* = 2.1 Hz, 1H, H_3_, C_6_H_2_), 3.98 (s, 3H, OCH_3_), 3.95 (s, 3H, OCH_3_). **^13^C{^1^H} NMR** (75.47 MHz, CD_2_Cl_2_): δ = 164.92 (s, C_2_–O, C_6_H_2_), 163.69 (s, C=N), 161.08 (s, C=O), 161.03 (s, C_4_–O, C_6_H_2_), 157.75 (s, C, C_6′_, phen), 156.89 (s, C, C_10′_, phen) 153.06 (s, CH, C_2′_, phen), 150.24 (s, CH, C_13′_, phen), 148.09 (s, =CH, C_olef_), 141.09 (s, CH, C_11′_, phen), 140.79 (s, CH, C_4′_, phen), 135.86 (s, =CH, C_vinyl_), 134.19 (s, C_i_, C_6_H_5_), 132.89 (s, CH, C_p_, C_6_H_5_), 131.19 (s, C, C_5′_, phen), 131.12 (s, C, C_9′_, phen), 130.94 (s, =C), 130.02 (s, C_o_, C_6_H_5_), 129.49 (s, C_m_, C_6_H_5_), 128.45 (2C overlapped, CH, C_7′_, C_8′_, phen), 127.77 (s, CH, C_3′_, phen), 125.99 (s, CH, C_12′_, phen), 121.23 (s, C_6_-Pd, C_6_H_2_), 119.08 (s, C_1_, C_6_H_2_), 116.43 (s, CH, C_5_, C_6_H_2_), 111.39 (s, =CH, C_olef_), 95.83 (s, CH, C_3_, C_6_H_2_), 56.74 (s, OCH_3_), 56.71 (s, OCH_3_).

## 4. Conclusions

New oxazolones that are structurally related to the chromophore of the Kaede protein were prepared and orthopalladated selectively at the 4-arylidene ring. Different anionic and neutral ancillary chelating ligands were coordinated to the [Pd(C,N-oxazolone)]^+^ fragment, with this structural unit showing a higher synthetic versatility than previous examples derived from the chromophore of the GFP. However, the structural characterization by X-ray diffraction of one representative showed that the ligands, mostly the oxazolone, are strongly distorted due to intramolecular repulsions. The fluorescence of these complexes was studied. Overall, the introduction of the Pd atom promotes an increase in the quantum yield of the fluorescence of the oxazolone **1a** by up to one order of magnitude (from <1% to 12%). However, other factors must also be considered, such as the push–pull character of the oxazolone, which is modulated by the arylidene substituents (OMe vs. F in this case), and its steric requirements, strongly dependent on the functionality in position 2 of the oxazolone (*E*-styryl in this case). Therefore, although oxazolones with structures related to the chromophore of the Kaede protein have advantages from the synthetic point of view, they do not provide noticeable improvements in the photophysical properties.

## Figures and Tables

**Table 1:** Selected bond distances (Å) and angles (°) for **7a**.

Pd(1)-N(1)	2.060(3)	N(1)-Pd(1)-N(3)	173.70(12)
Pd(1)-N(2)	2.110(3)	N(1)-Pd(1)-C(1)	98.62(13)
Pd(1)-N(3)	2.006(3)	N(2)-Pd(1)-N(3)	96.35(11)
Pd(1)-C(1)	2.013(3)	N(2)-Pd(1)-C(1)	177.06(12)
N(1)-Pd(1)-N(2)	78.55(11)	N(3)-Pd(1)-C(1)	86.53(12)

**Table 2:** Absorption maxima for compounds **1a**, **4a**, **6a**, **7a**, **8a, 1b** and **4b**. ^1^

Compound	λ_abs,max_ (nm)	Compound	λ_abs,max_ (nm)
**1a**	428	**1b**	381
**4a**	468	**4b**	446
**6a**	490		
**7a**	495	**8a**	494

^1^ Values in CH_2_Cl_2_ solution at 25 °C, concentration 5 × 10^−4^ M.

**Table 3:** Excitation–emission maxima, counts for compounds **1a**, **4a**, **6a**, **7a, 8a**, **1b** and **4b** and quantum yields for **1a** and **6a** ^1^

Compound	λ_exc,max_ (nm)	λ_emis,max_ (nm)	Counts	QY
**1a**	480, 331	505	2.7 × 10^4^	**<1%**
**1b**	420, 326	534, 493, 462, 437	2.4 × 10^4^	
**4a**	516, 480, 446	476, 453	1 × 10^6^	-
**4b**	480, 321	569 (h), 525	3.6 × 10^4^	-
**6a**	539, 469, 371	589	**5 × 10^6^**	**12% ^2^**
**7a**	555, 433, 390	599	3 × 10^6^	-
**8a**	529, 412	564	1.6 × 10^5^	-

^1^ Values in CH_2_Cl_2_ solution at 25 °C, concentration 5 × 10^−4^ M. ^2^ Concentration 10^−5^ M (λ**_exc_** = 490 nm).

## Data Availability

The data presented are available in the published version of the manuscript and Supplementary Materials.

## Supplementary Materials

The following are available online. NMR spectra, IR spectra, absorption spectra and excitation–emission spectra. Supplementary Material corresponding to the X-ray structure has been deposited in the CCDC as CCDC-2035173 (see Materials and Methods).

## Sample Availability

Samples of the compounds are not available from the authors.
