# Supplementary material for: Fluorescent Orthopalladated Complexes of 4-Aryliden-5(4H)-oxazolones from the Kaede Protein: Synthesis and Characterization
Source: Molecules. 2021 Feb 25;26(5):1238. doi: 10.3390/molecules26051238 (PMC7956804; doi:10.3390/molecules26051238)
Supplement: Supplementary file 1 [file molecules-26-01238-s001.pdf]

SUPPLEMENTARY MATERIAL

NMR spectra

IR spectra

Absorption spectra

Excitation-Emission spectra

Crystallographic Tables

1. NMR SPECTRA

1.1. Oxazolone **1a**

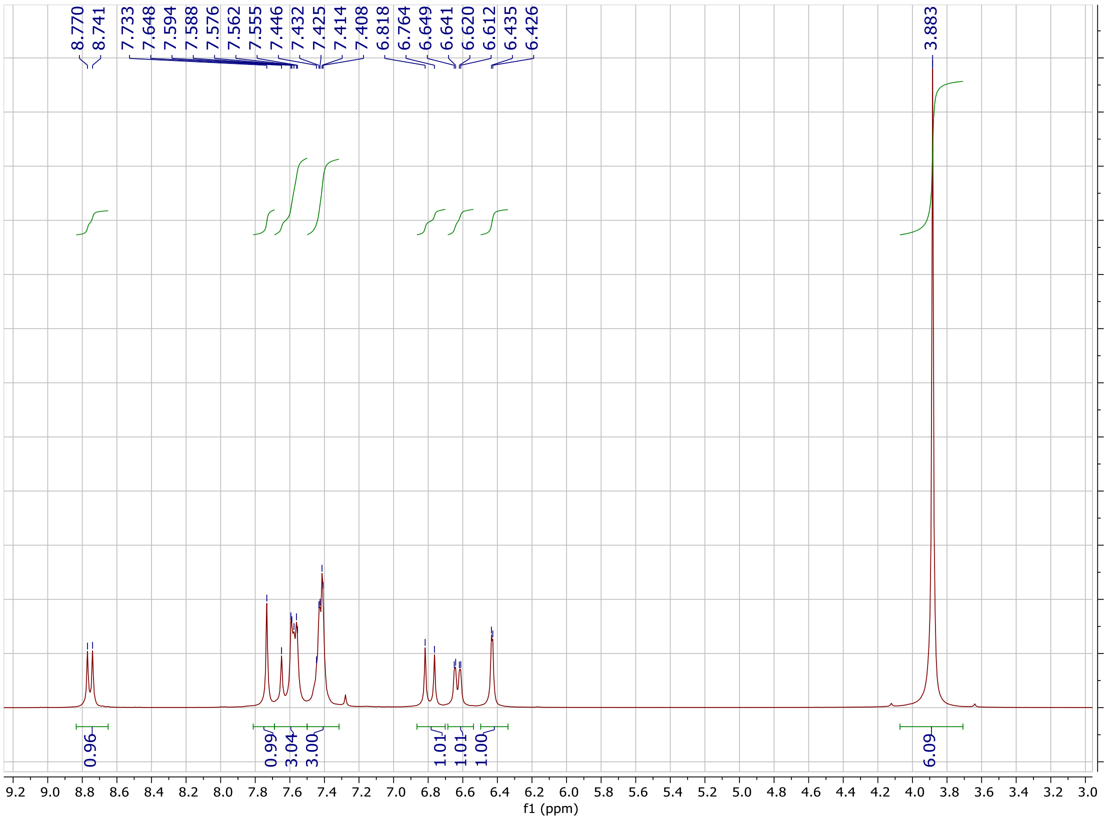

^{1}H-NMR spectrum ($CDCl_3$, 300.13 MHz) of **1a**

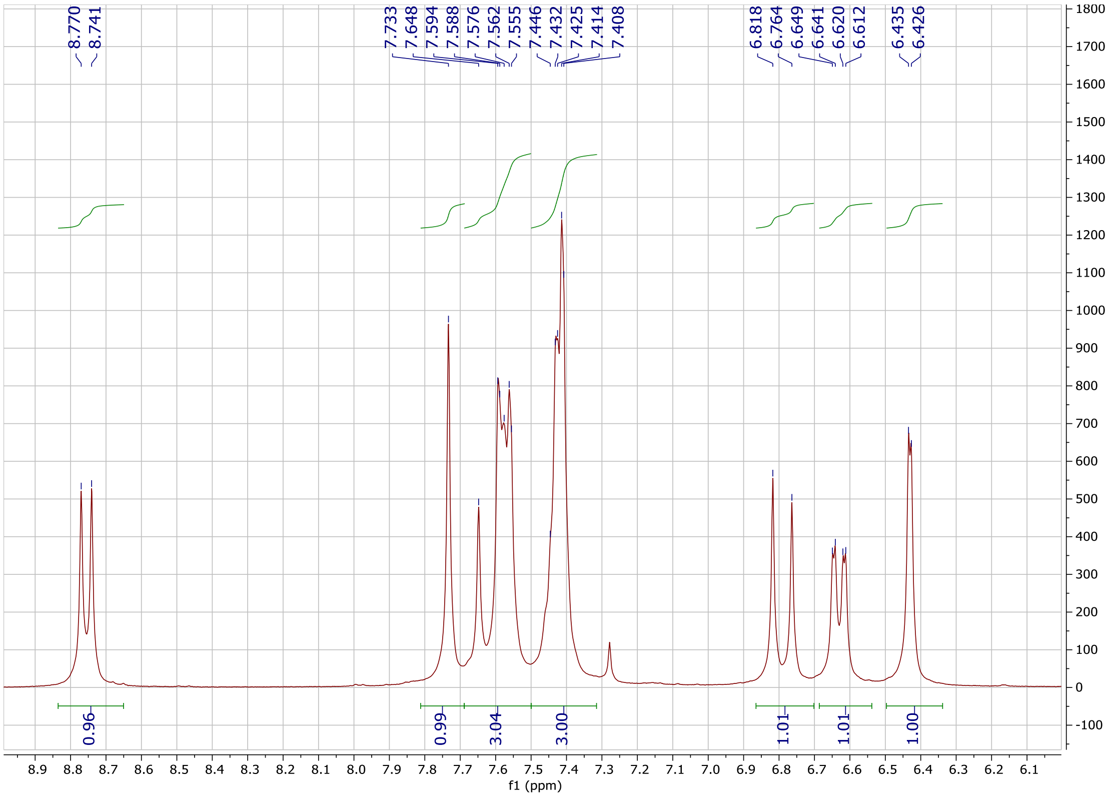

^{1}H-NMR spectrum ($CDCl_3$, 300.13 MHz) of **1a**, zoom aromatic region

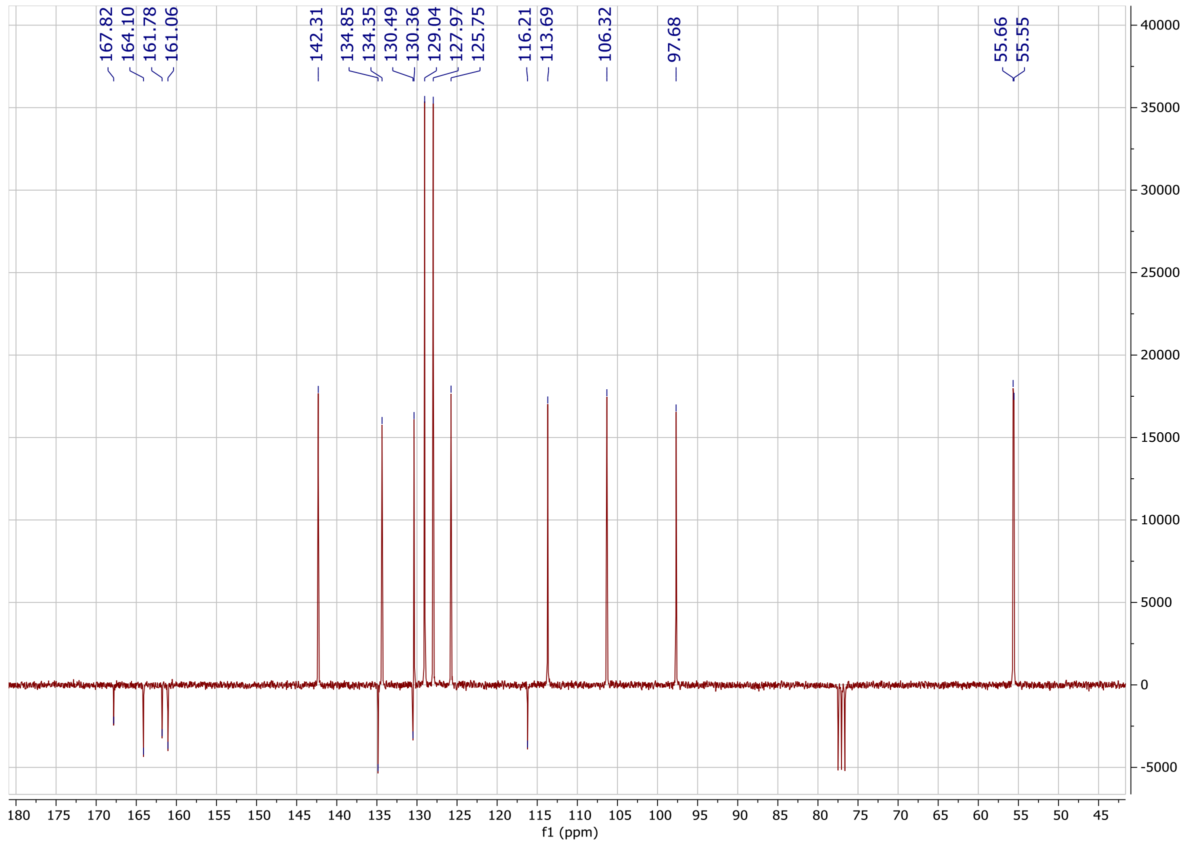

$^{13}C\{^{1}H\}$ (APT) NMR spectrum ($CDCl_3$, 75.47 MHz) of **1a**

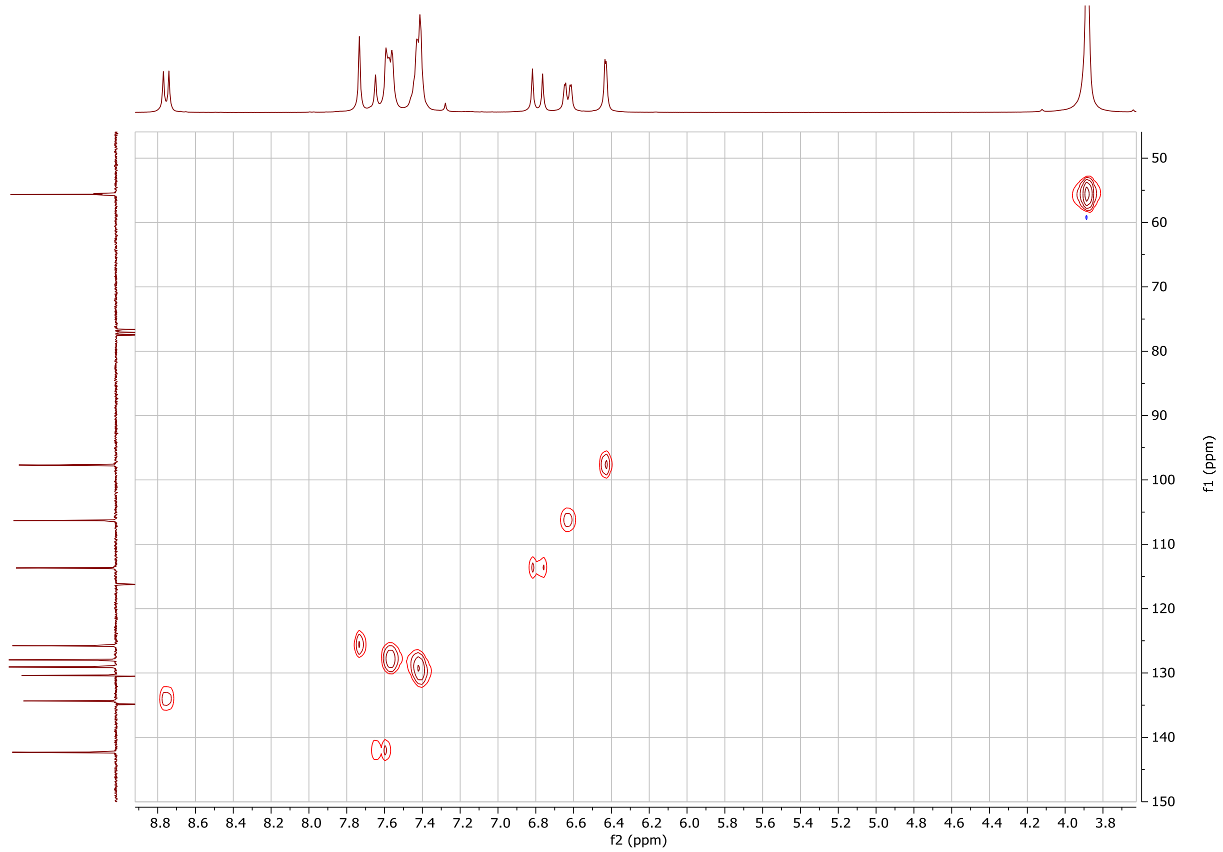

^{1}H-^{13}C HSQC correlation spectrum of **1a**

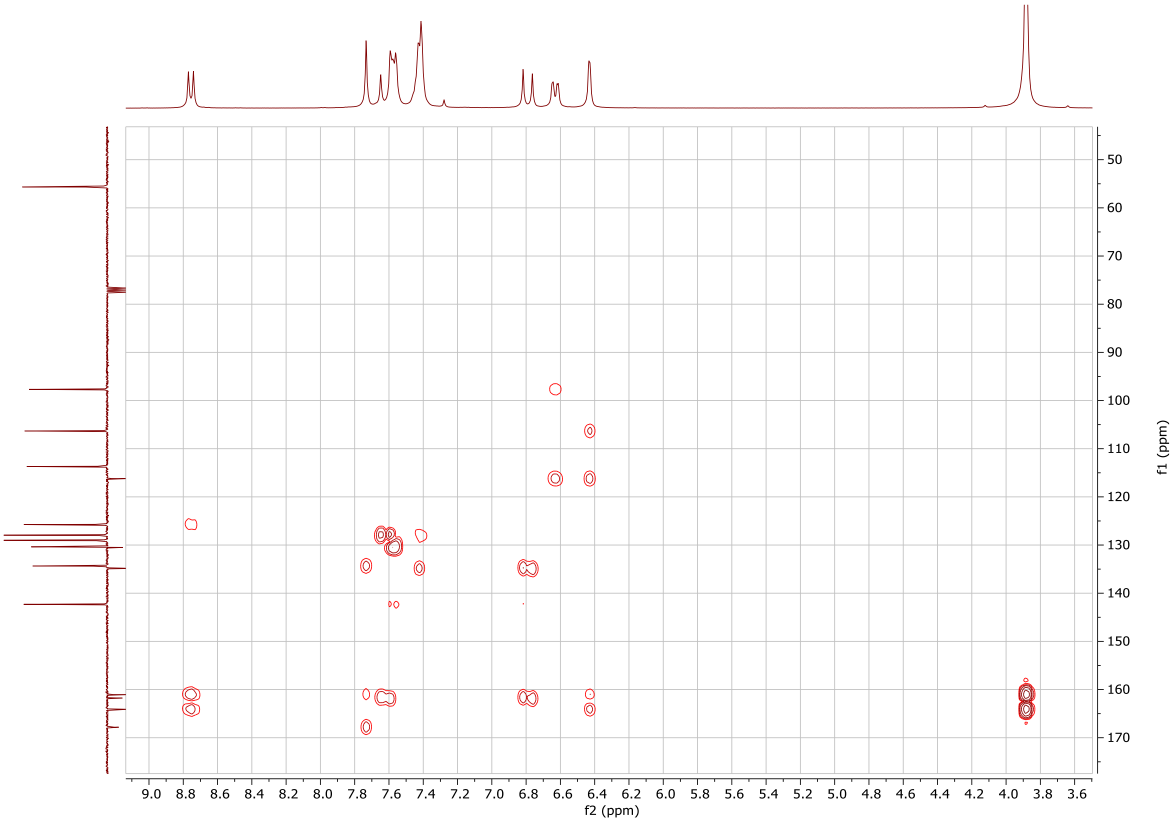

^1H-^{13}C HMBC correlation spectrum of **1a**

1.2. Oxazolone **1b**

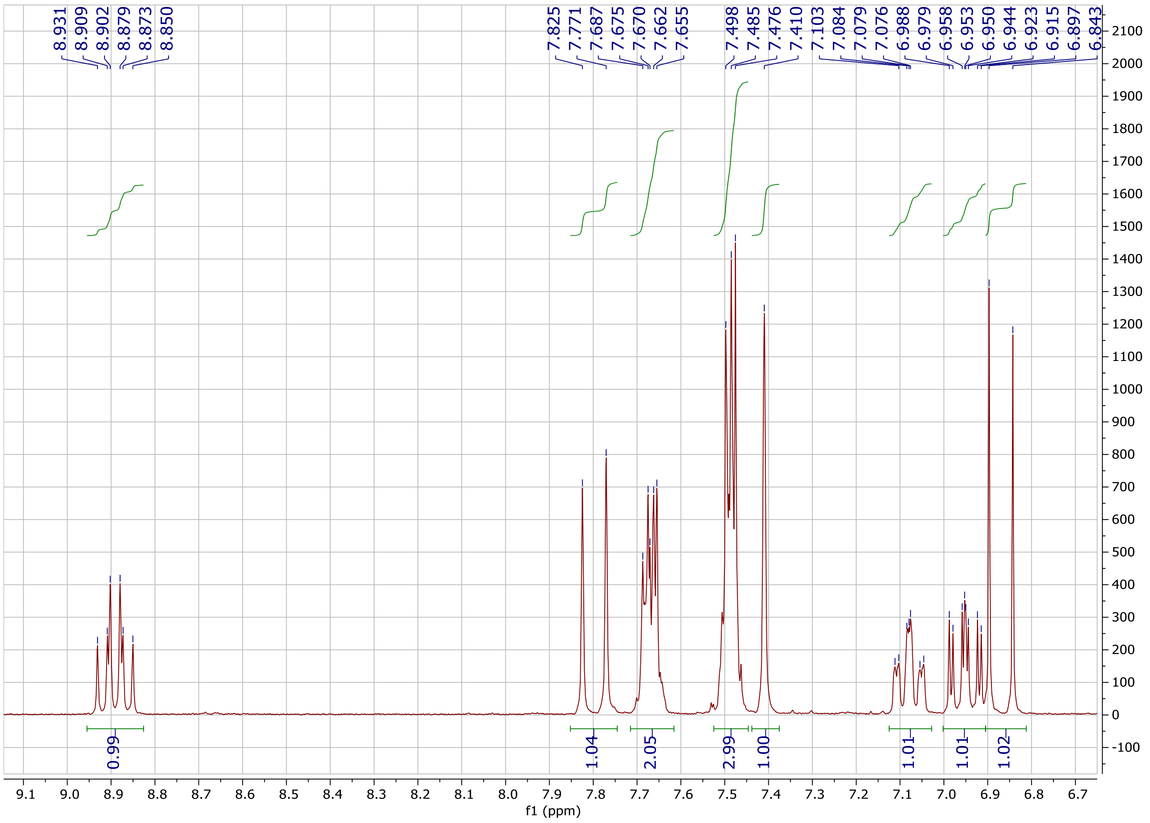

^1H-NMR spectrum (CD_2Cl_2, 300.13 MHz) of **1b**

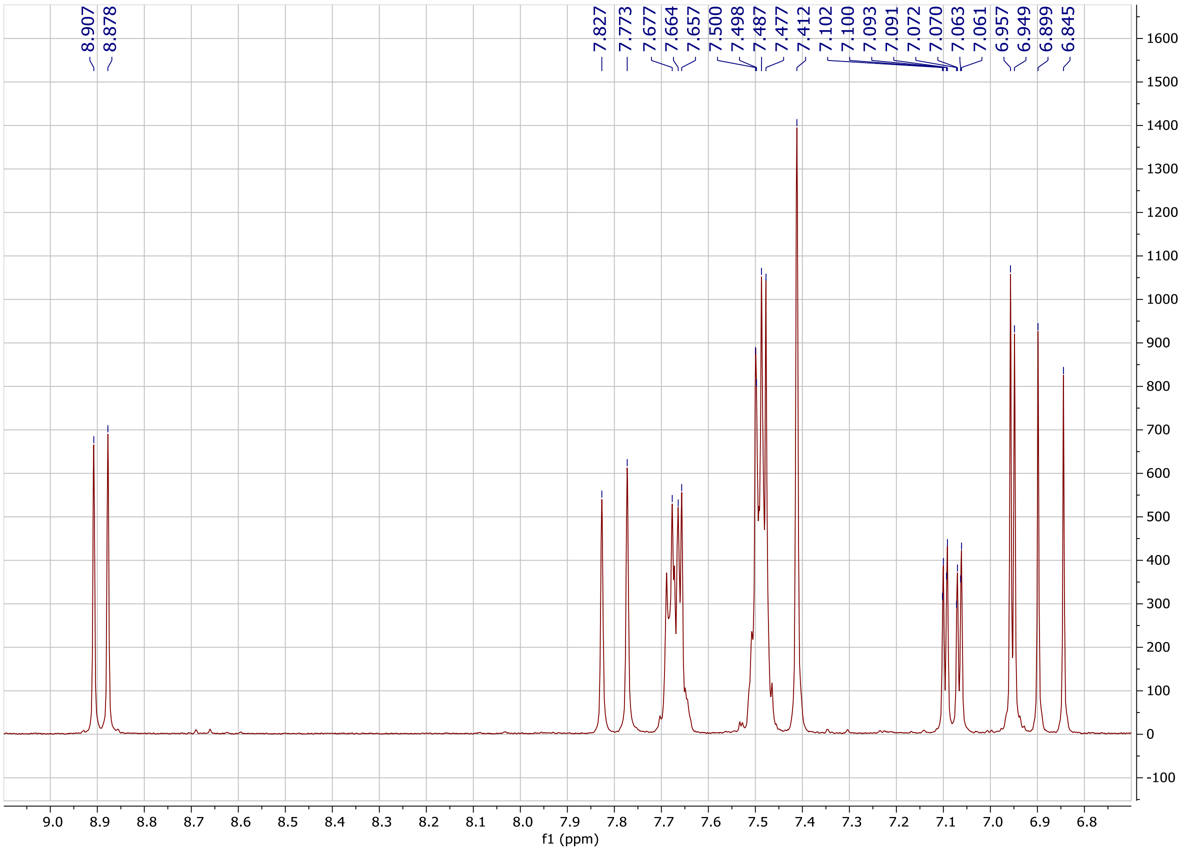

$^{1}H\{^{19}F\}$ NMR spectrum (CD_2Cl_2, 300.13 MHz) of **1b**

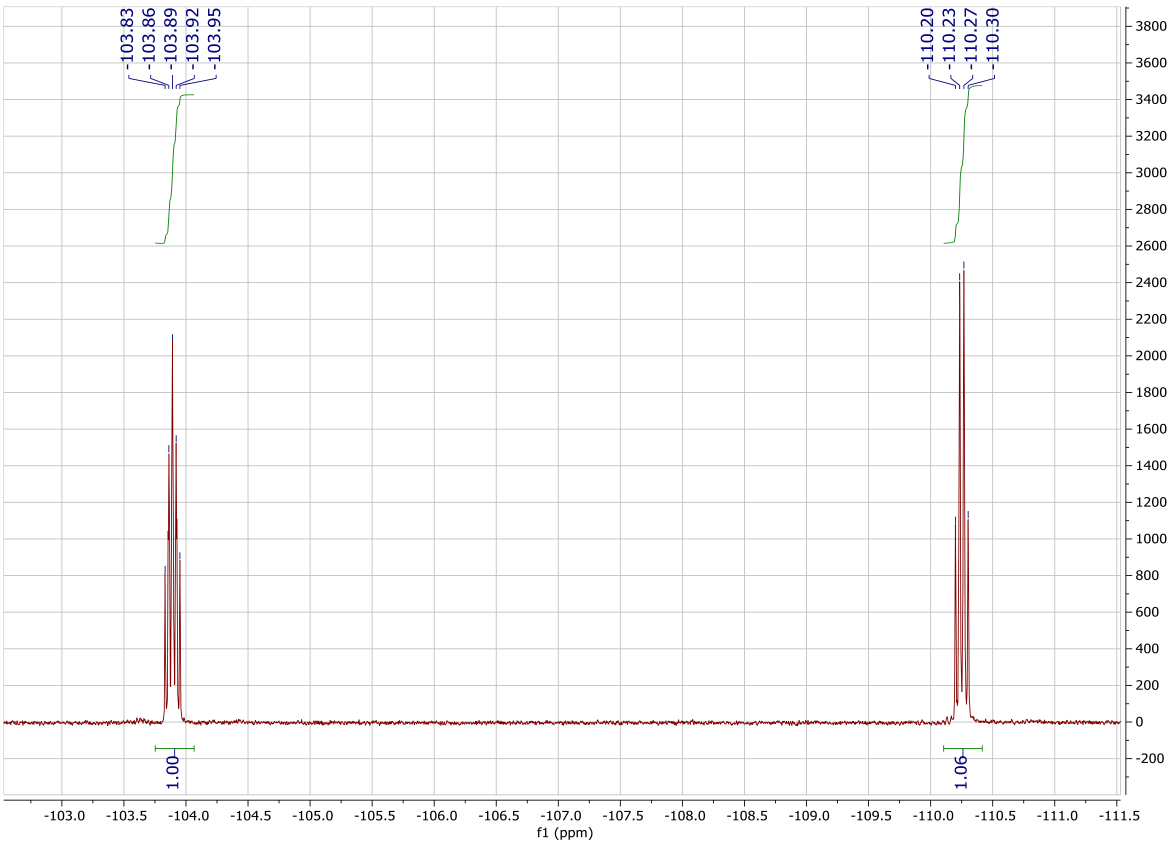

^{19}F-NMR spectrum (CD_2Cl_2, 282.40 MHz) of **1b**

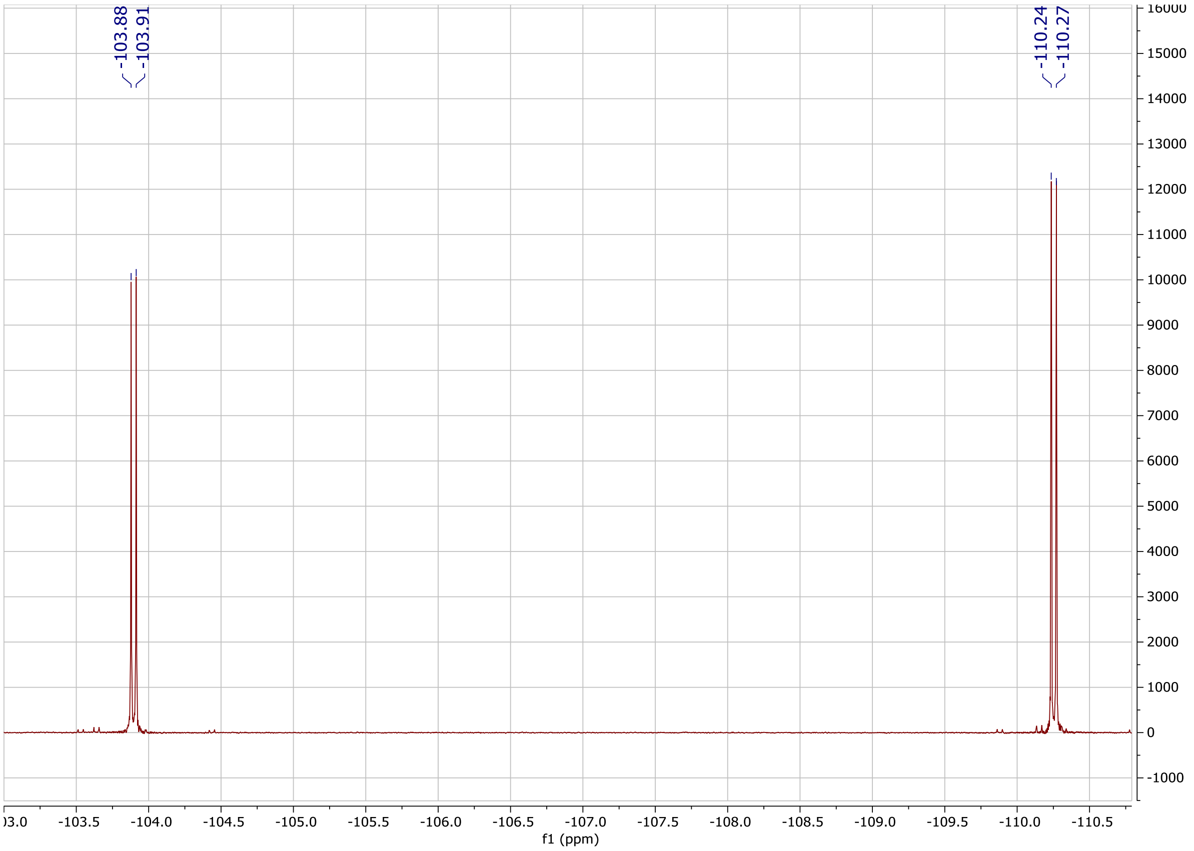

$^{19}F\{^{1}H\}$ NMR spectrum (CD_2Cl_2, 282.40 MHz) of **1b**

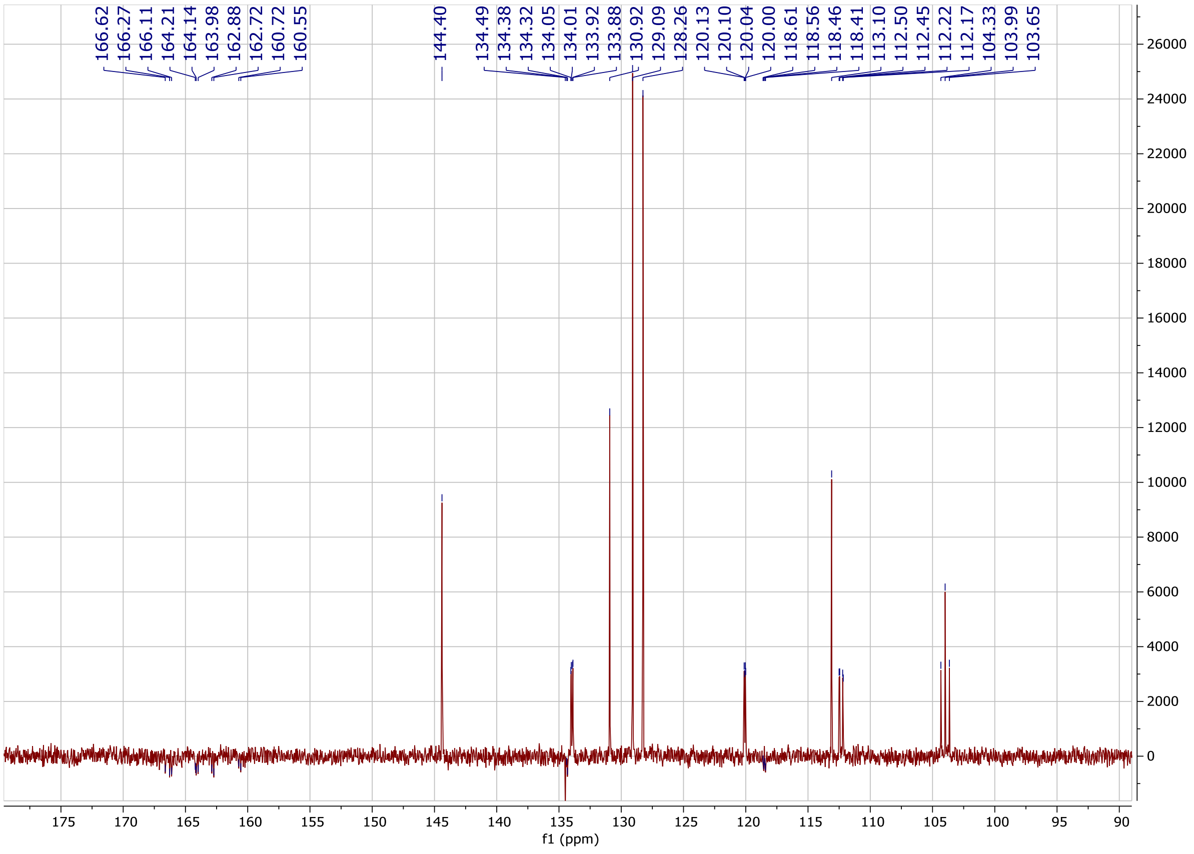

$^{13}C\{^{1}H\}$-(APT) NMR spectrum (CD_2Cl_2, 75.47 MHz) of **1b**

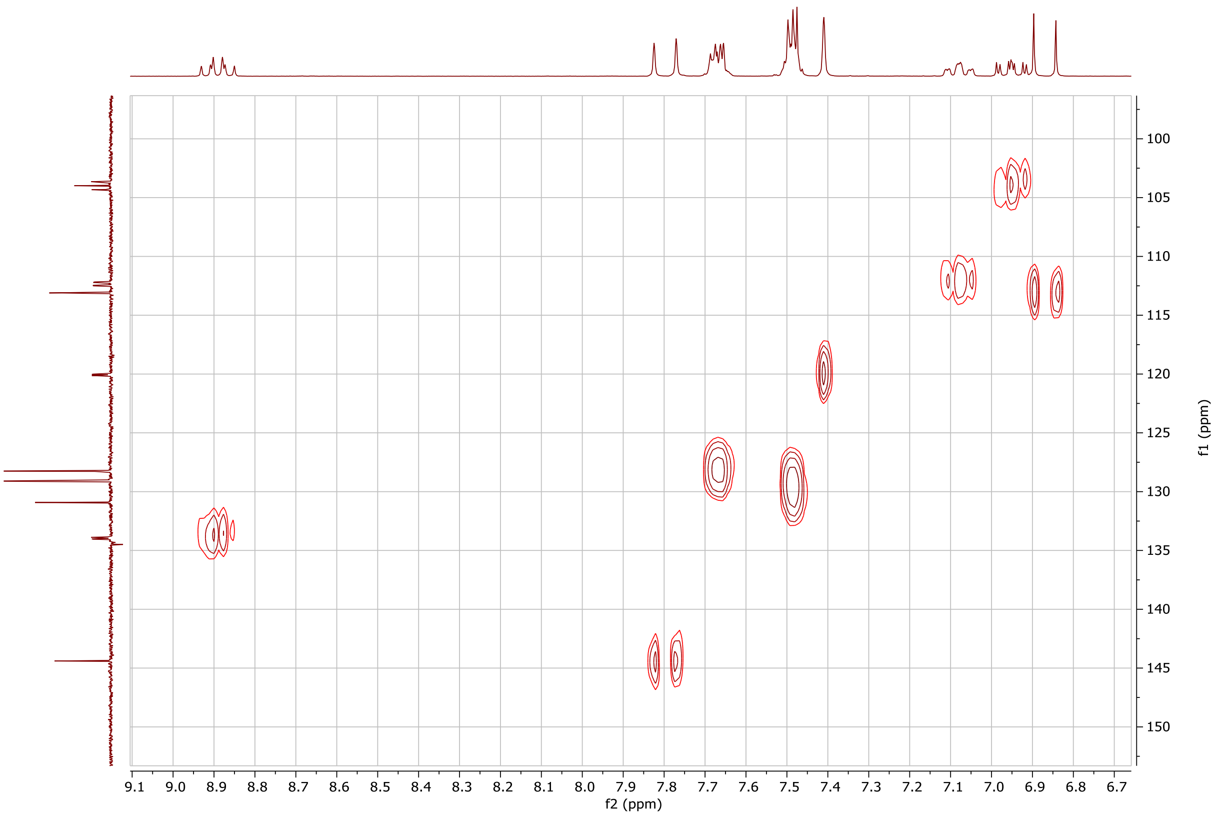

^{1}H-^{13}C HSQC correlation spectrum of **1b**

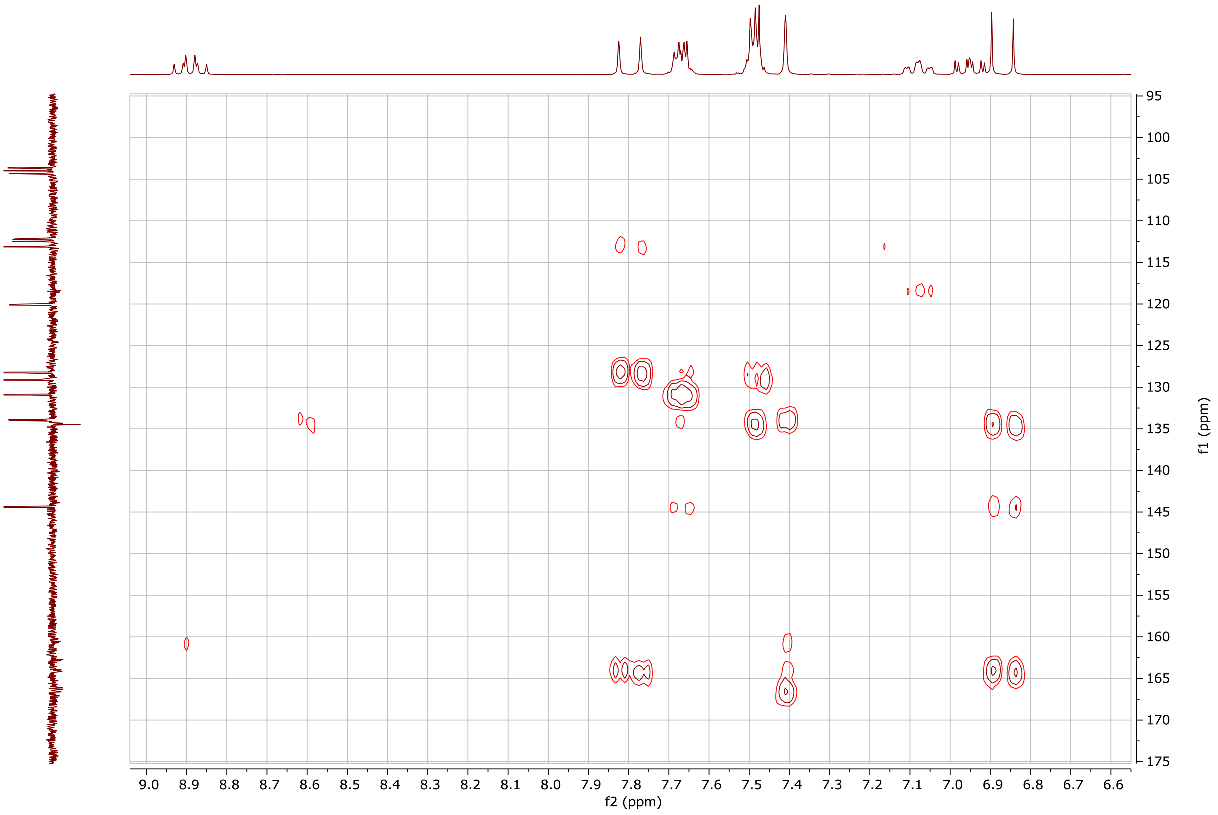

^{1}H-^{13}C HMBC correlation spectrum of **1b**

1.3. Ortho-palladated complex **2a**

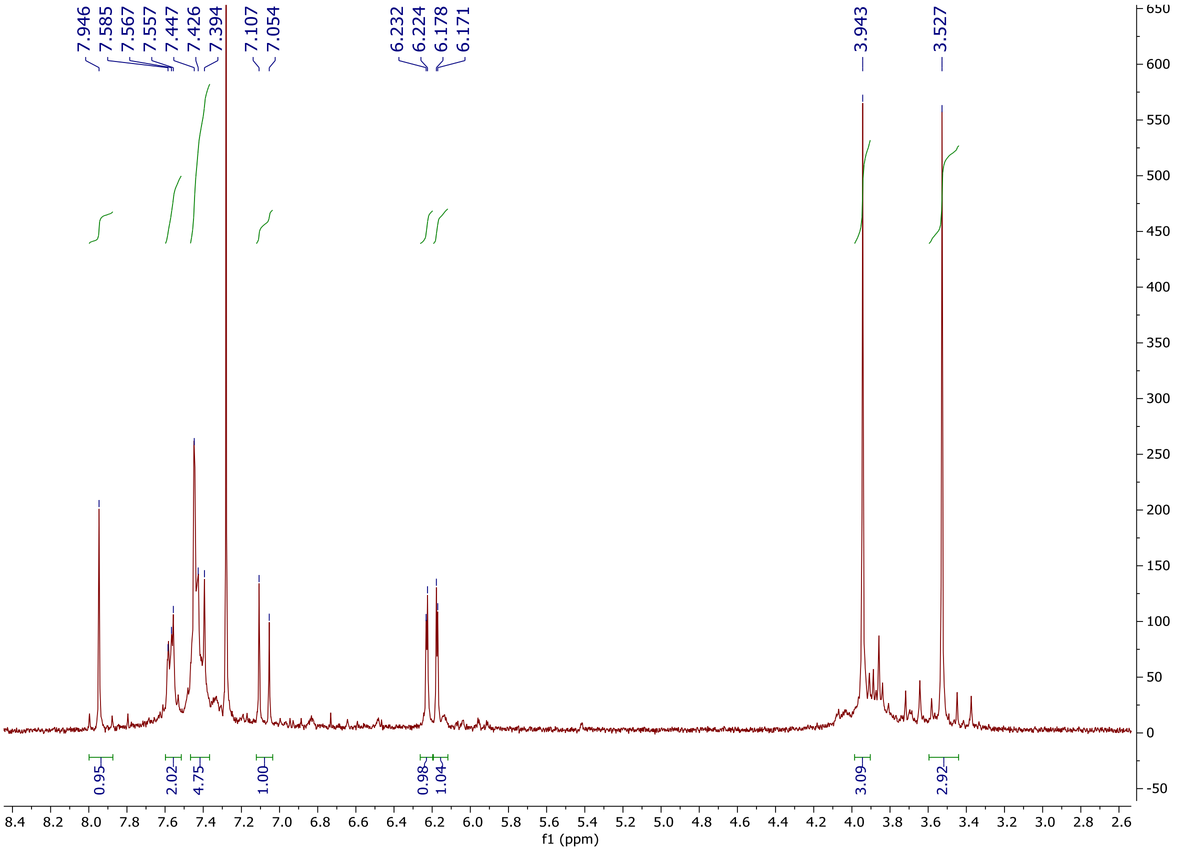

^{1}H-NMR spectrum ($CDCl_3$, 300.13 MHz) of **2a**

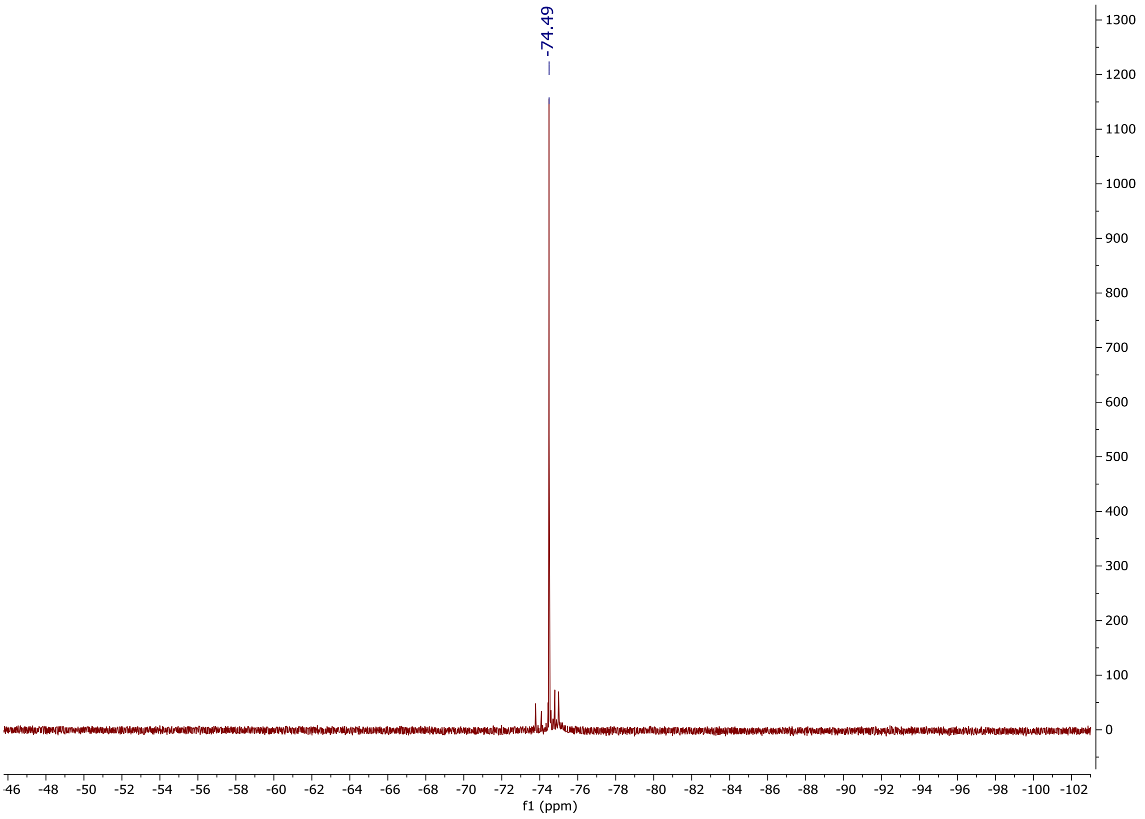

^{19}F-NMR spectrum ($CDCl_3$, 282.40 MHz) of **2a**

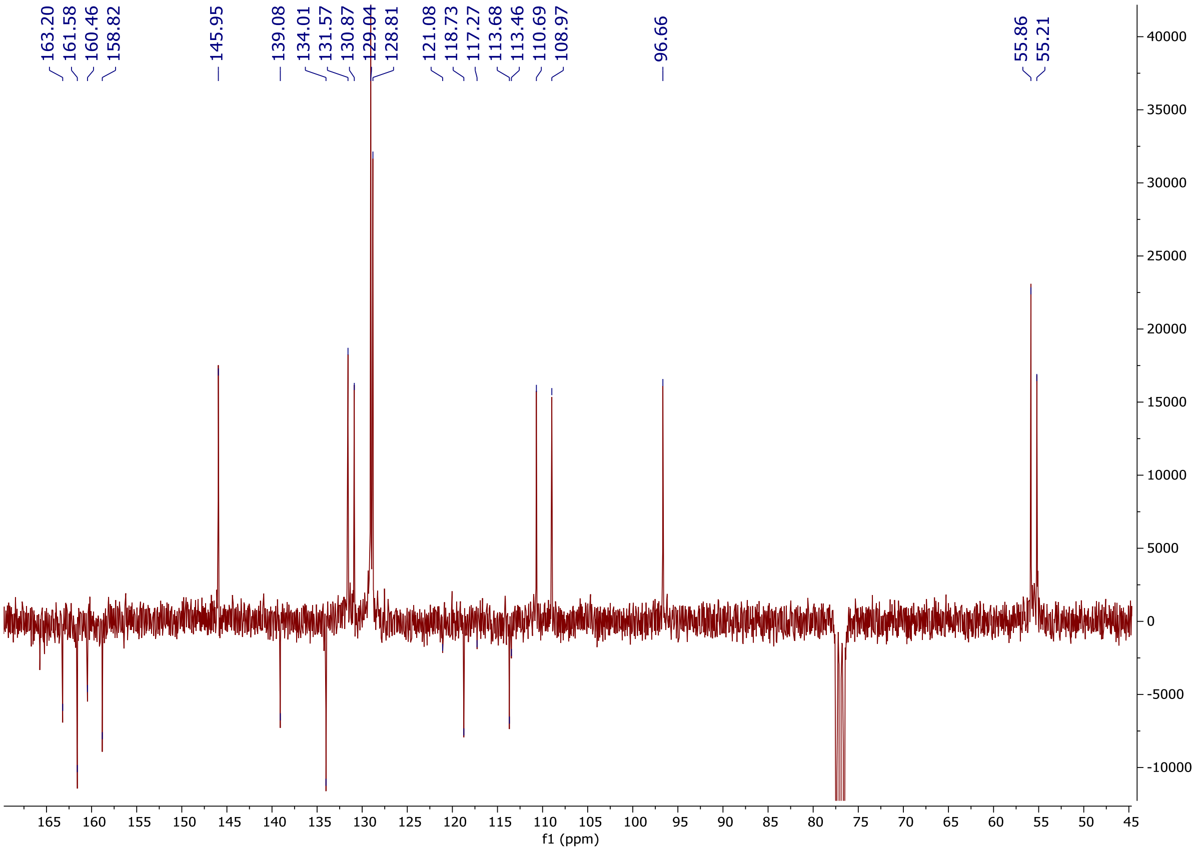

$^{13}C\{^{1}H\}$-(APT) NMR spectrum ($CDCl_3$, 75.47 MHz) of **2a**

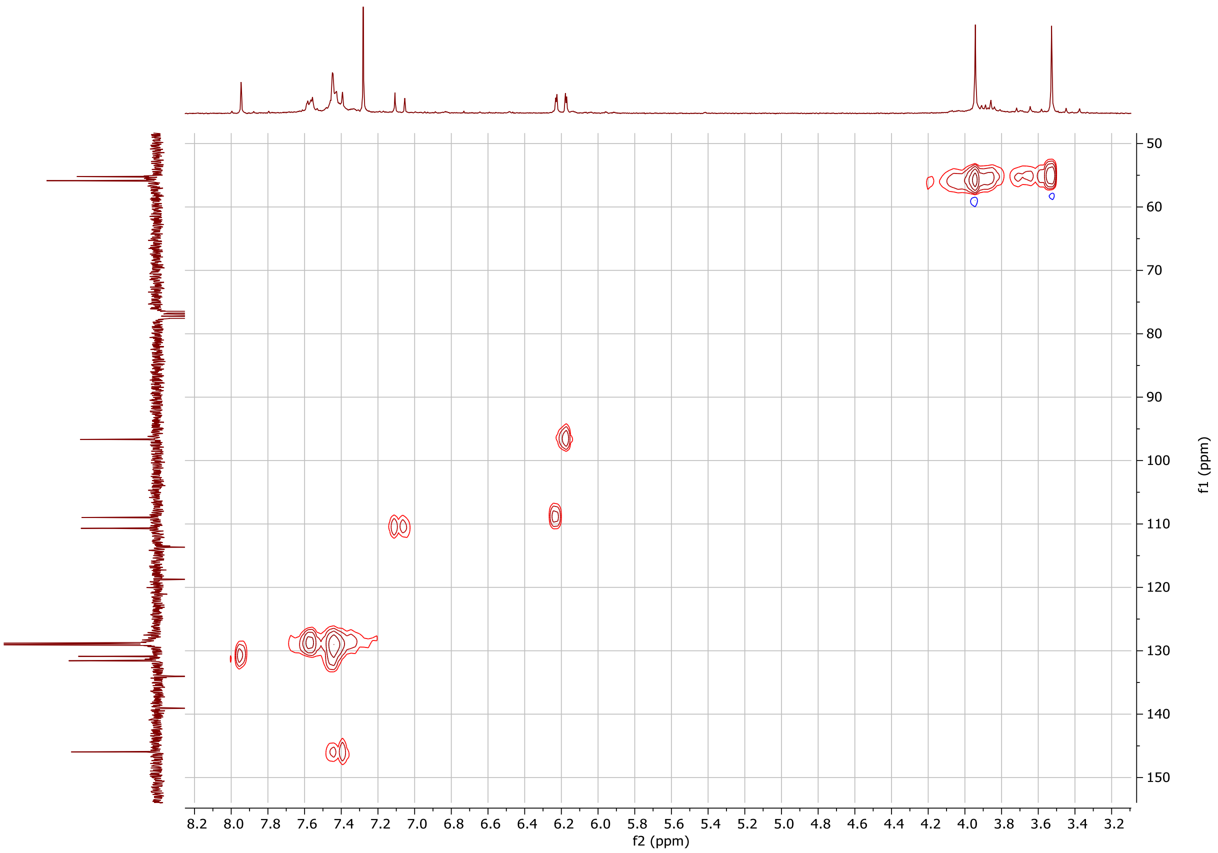

^{1}H-^{13}C HSQC correlation spectrum of **2a**

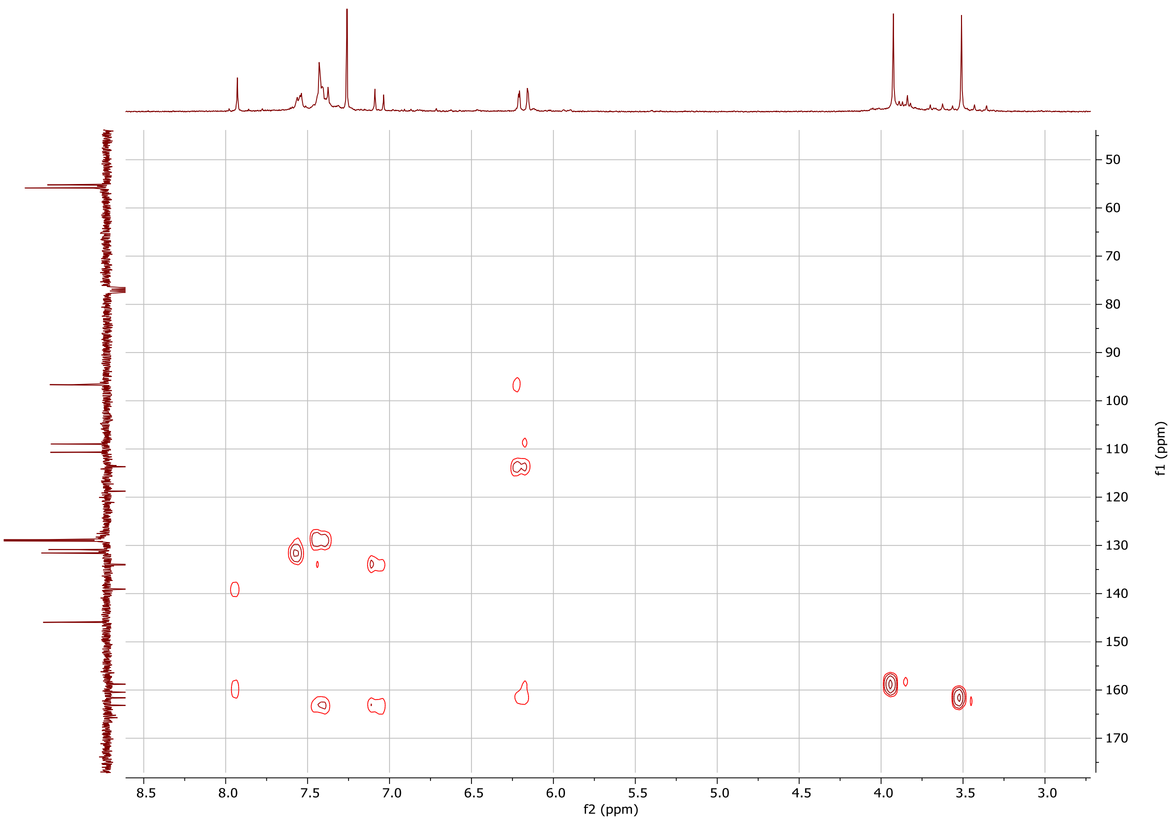

^{1}H-^{13}C HMBC correlation spectrum of **2a**

1.4. Mixture of ortho-palladated complexes **2b**

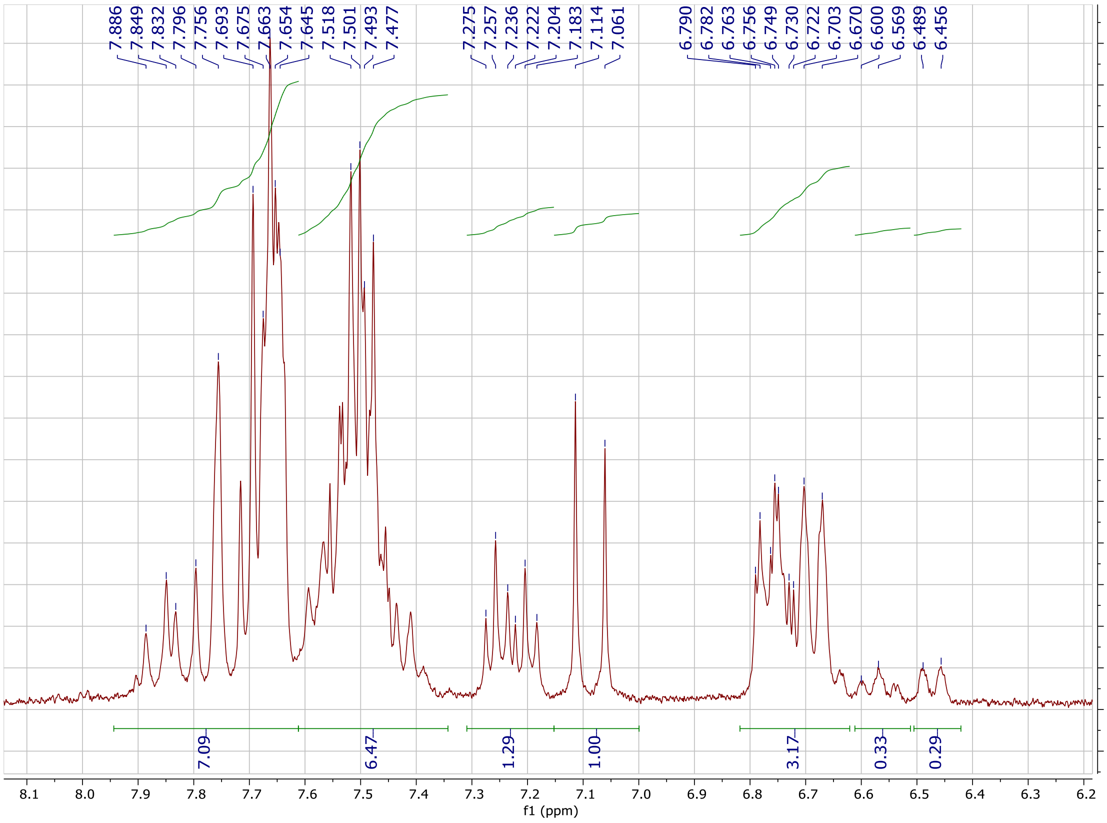

^{1}H-NMR spectrum (CD_2Cl_2, 300.13 MHz) of **2b**

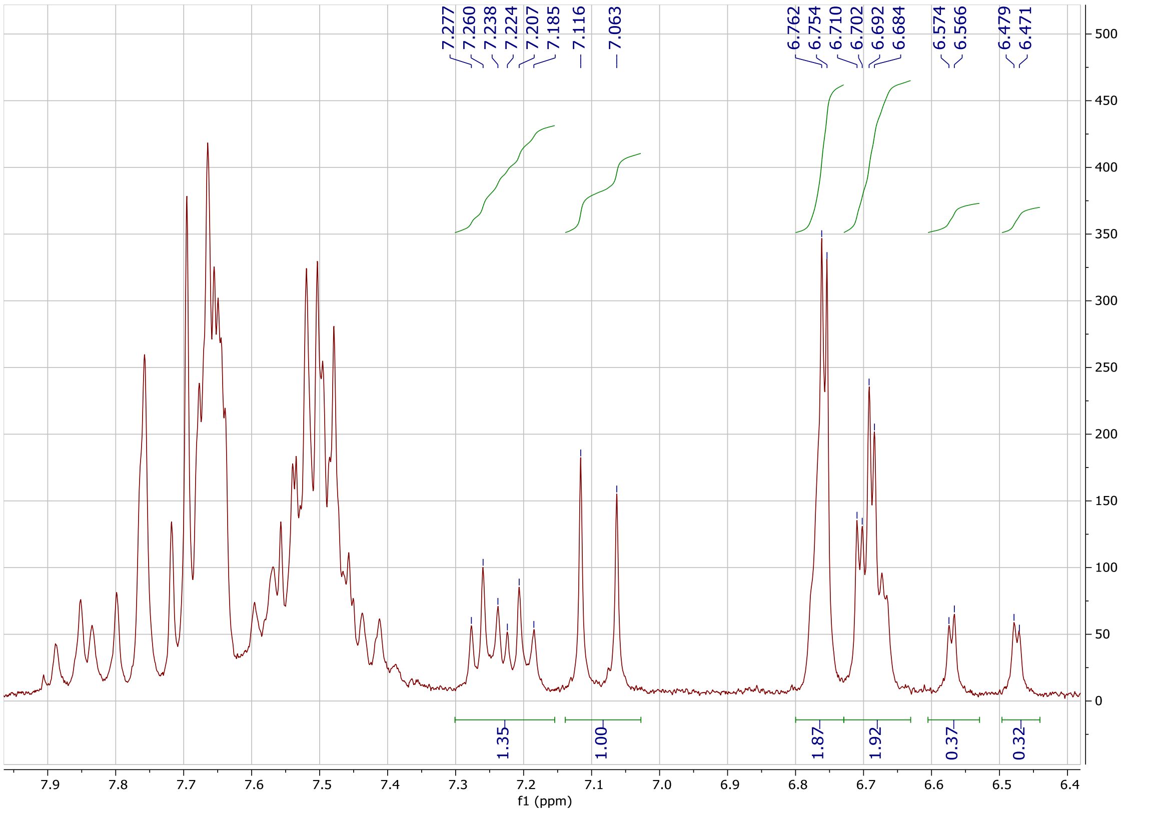

$^{1}H\{^{19}F\}$ NMR spectrum (CD_2Cl_2, 300.13 MHz) of **2b**

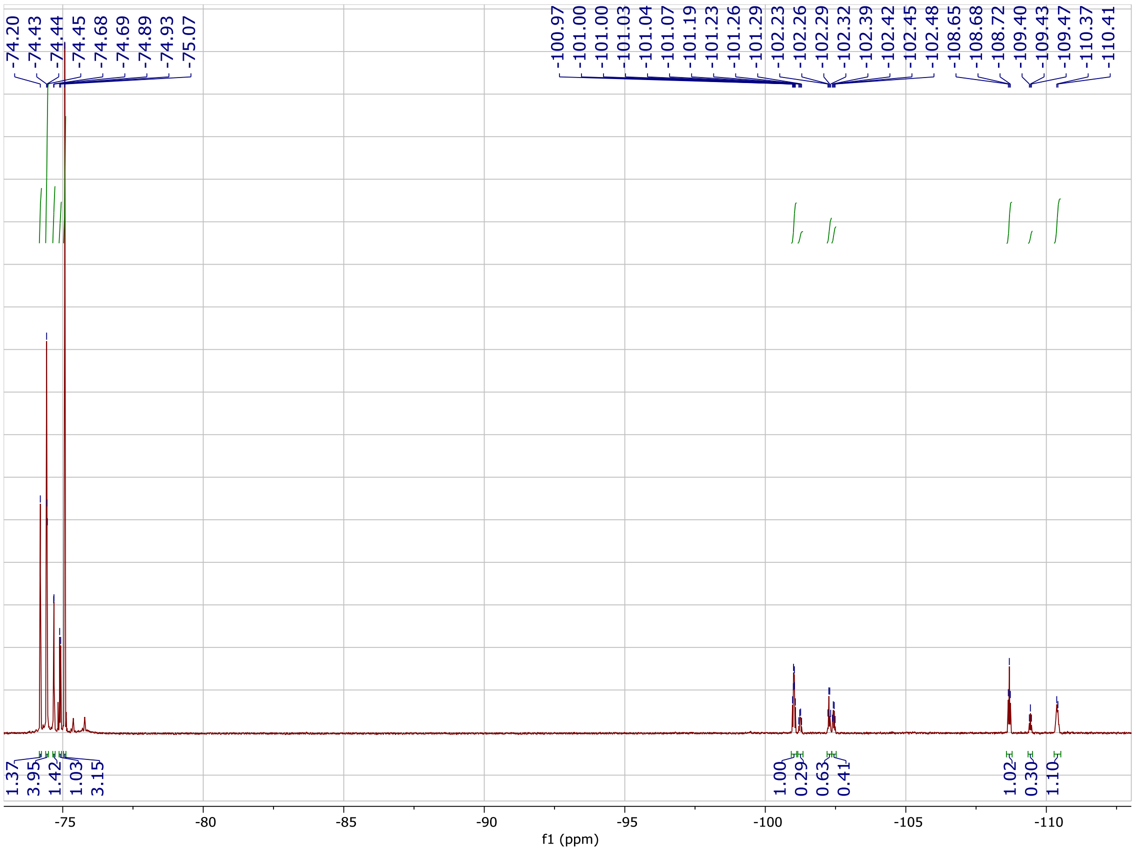

^{19}F-NMR spectrum (CD_2Cl_2, 282.40 MHz) of **2b**

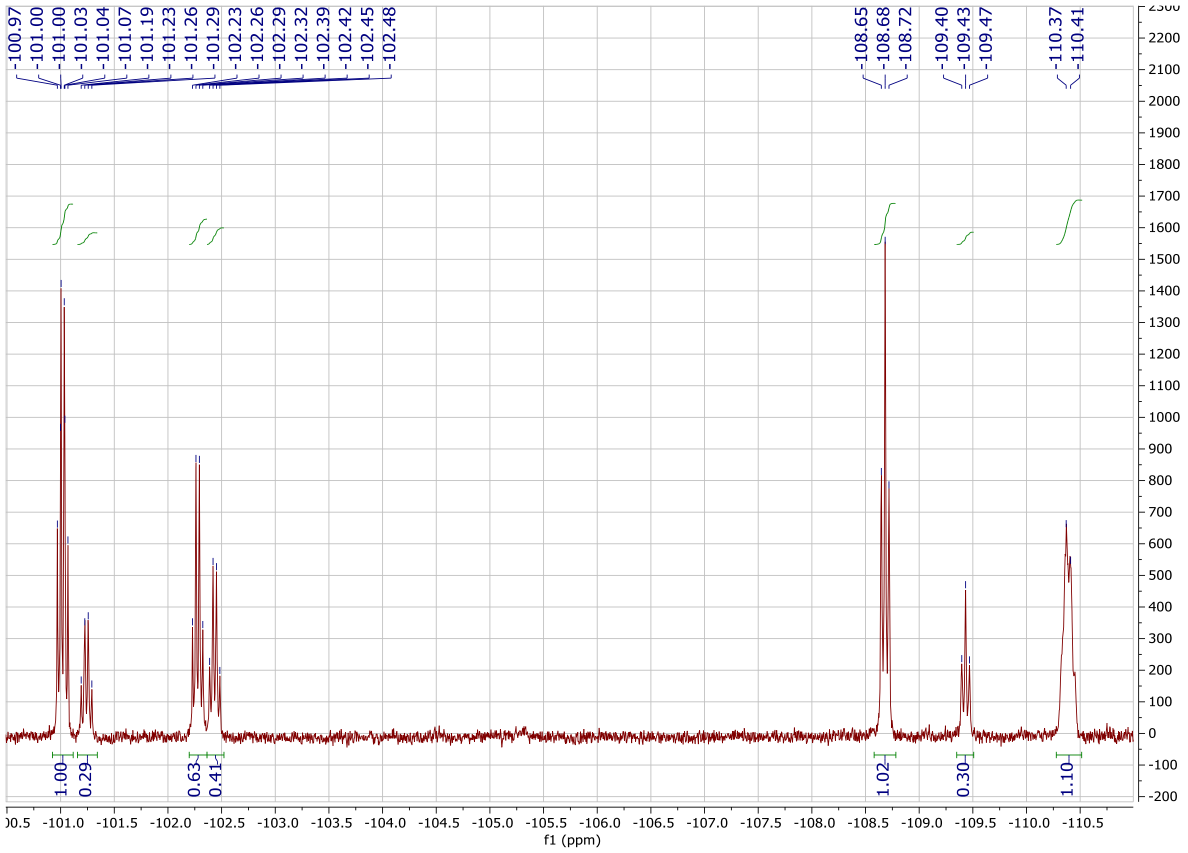

^{19}F-NMR spectrum (CD_2Cl_2, 282.40 MHz) of **2b** (region Ar-F)

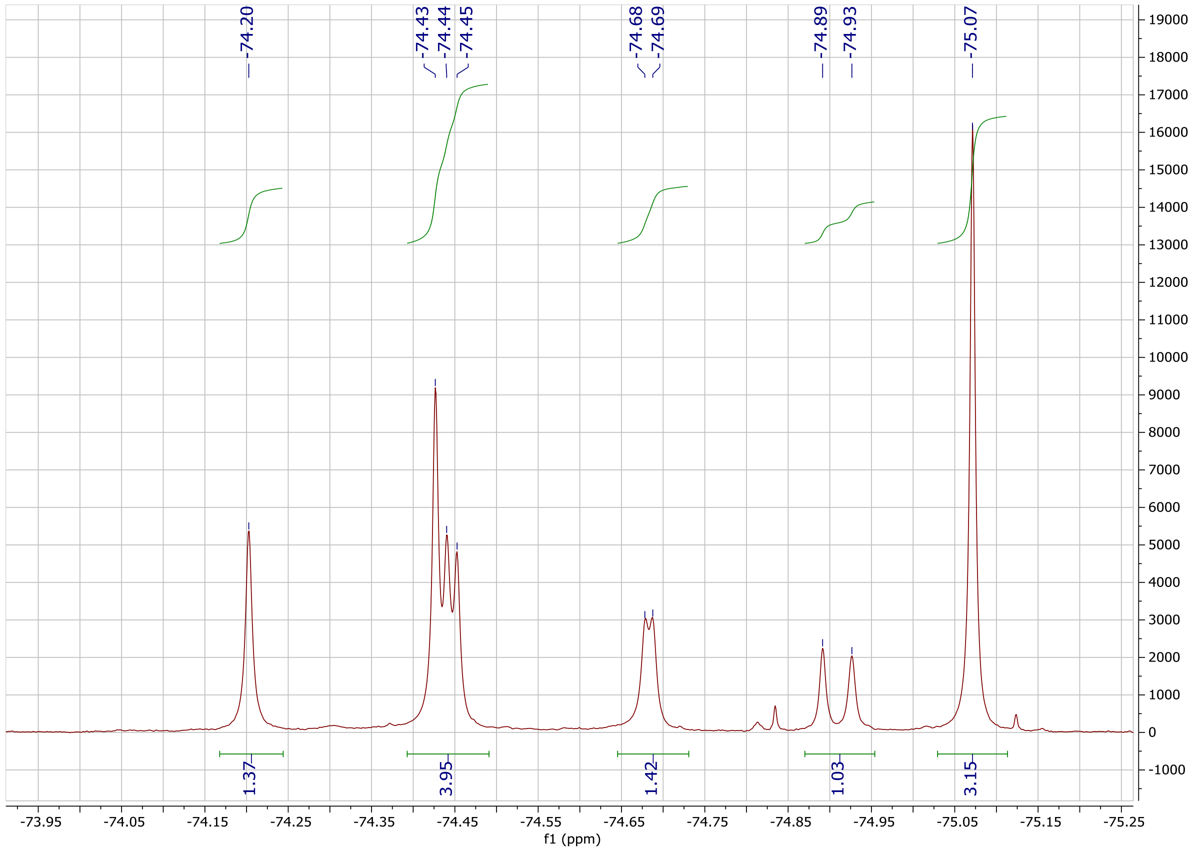

^{19}F-NMR spectrum (CD_2Cl_2, 282.40 MHz) of **2b** (region CF_3)

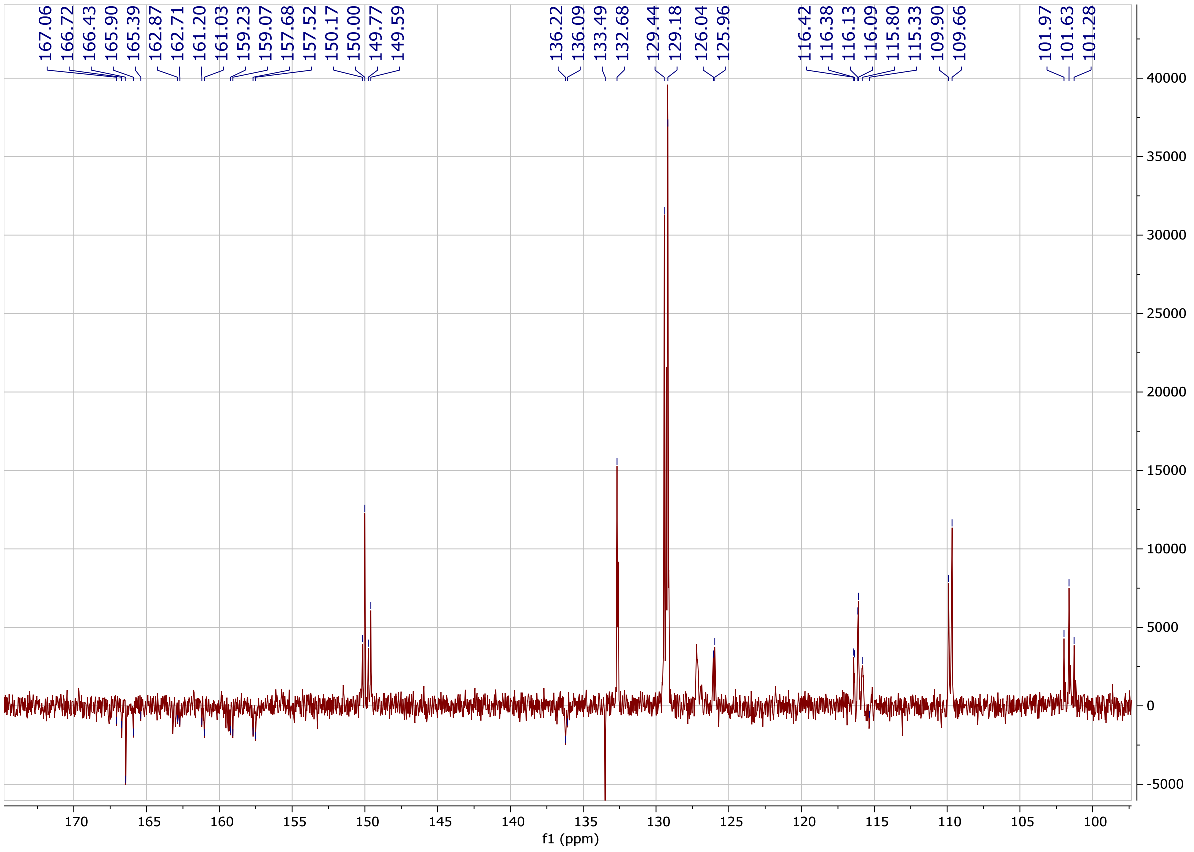

$^{13}C\{^{1}H\}$-(APT) NMR spectrum (CD_2Cl_2, 75.47 MHz) of **2b**

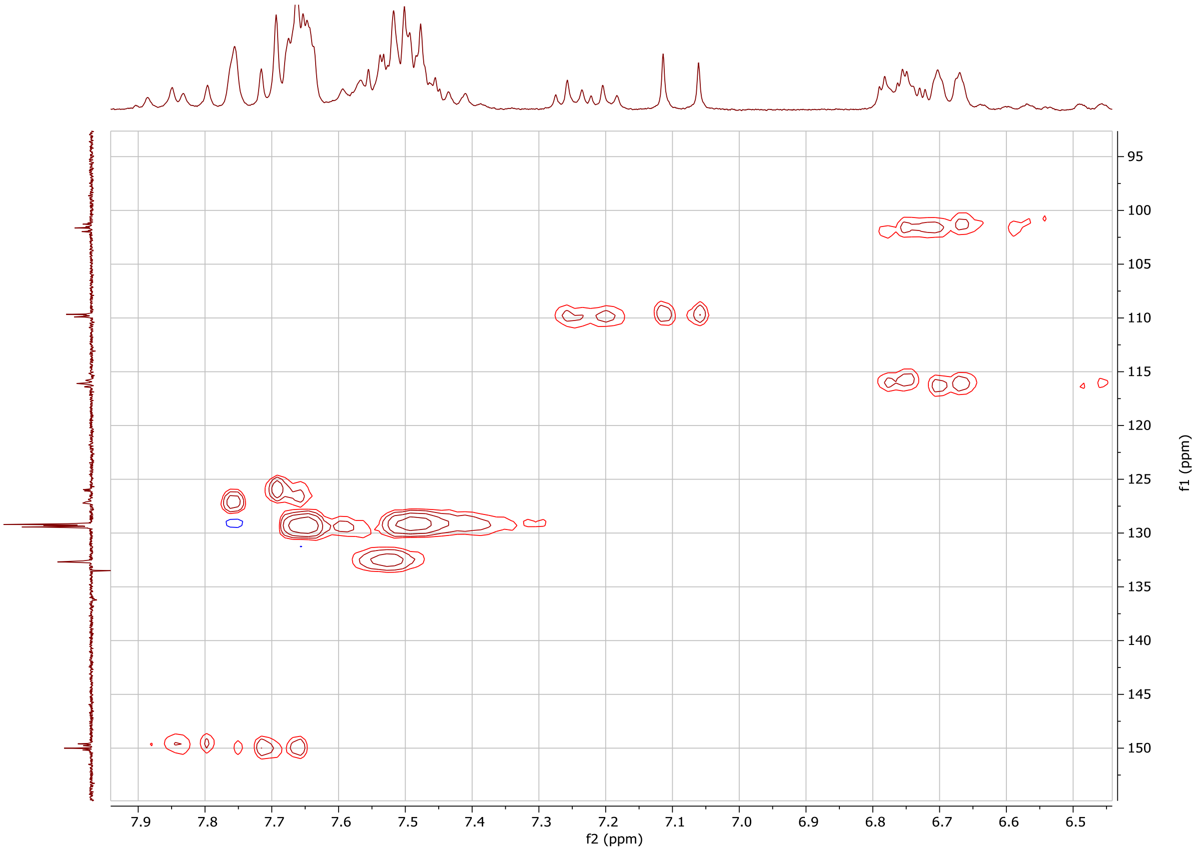

^{1}H-^{13}C HSQC correlation spectrum of **2b**

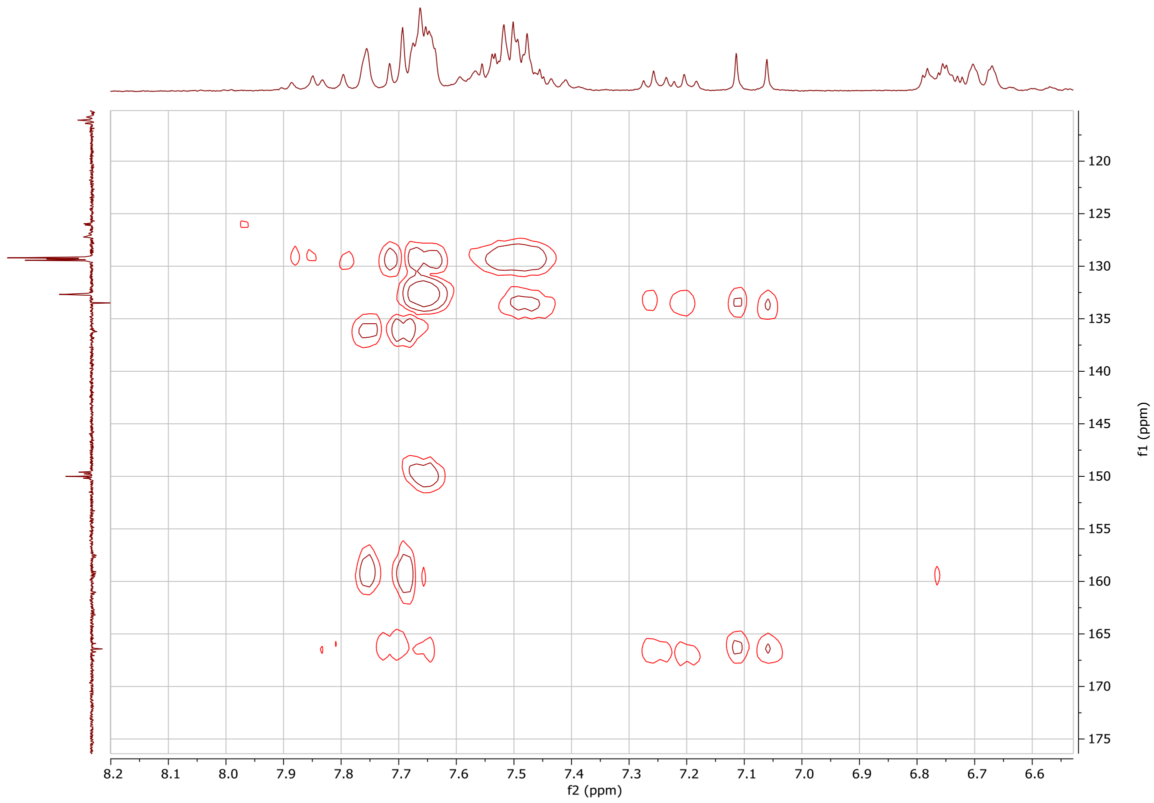

^{1}H-^{13}C HMBC correlation spectrum of **2b**

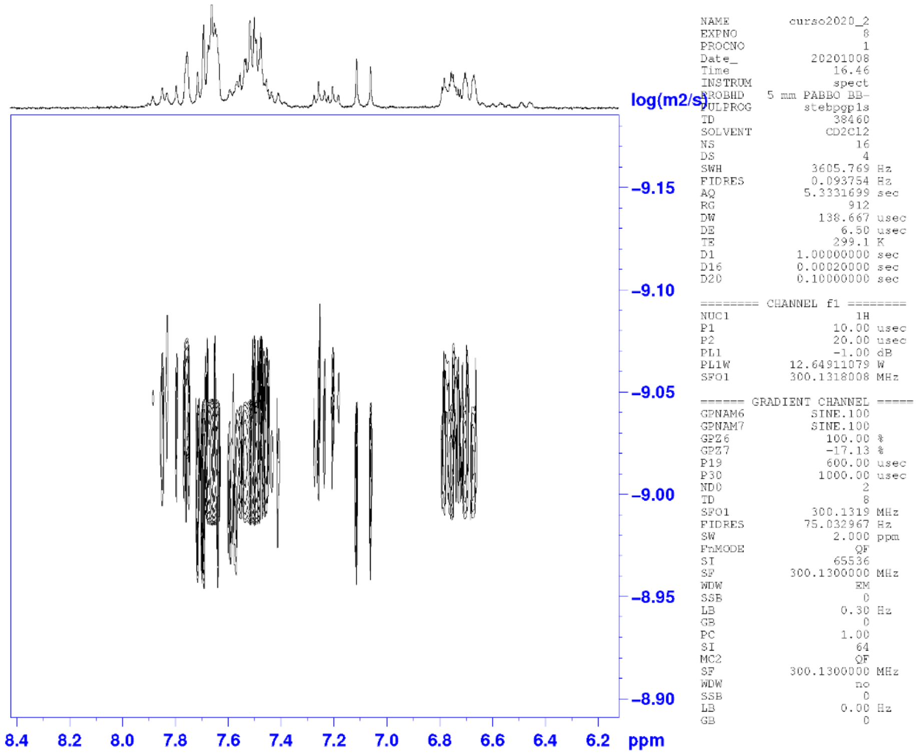

DOSY spectrum of **2b**

1.5. Orthopalladated complex **4a**

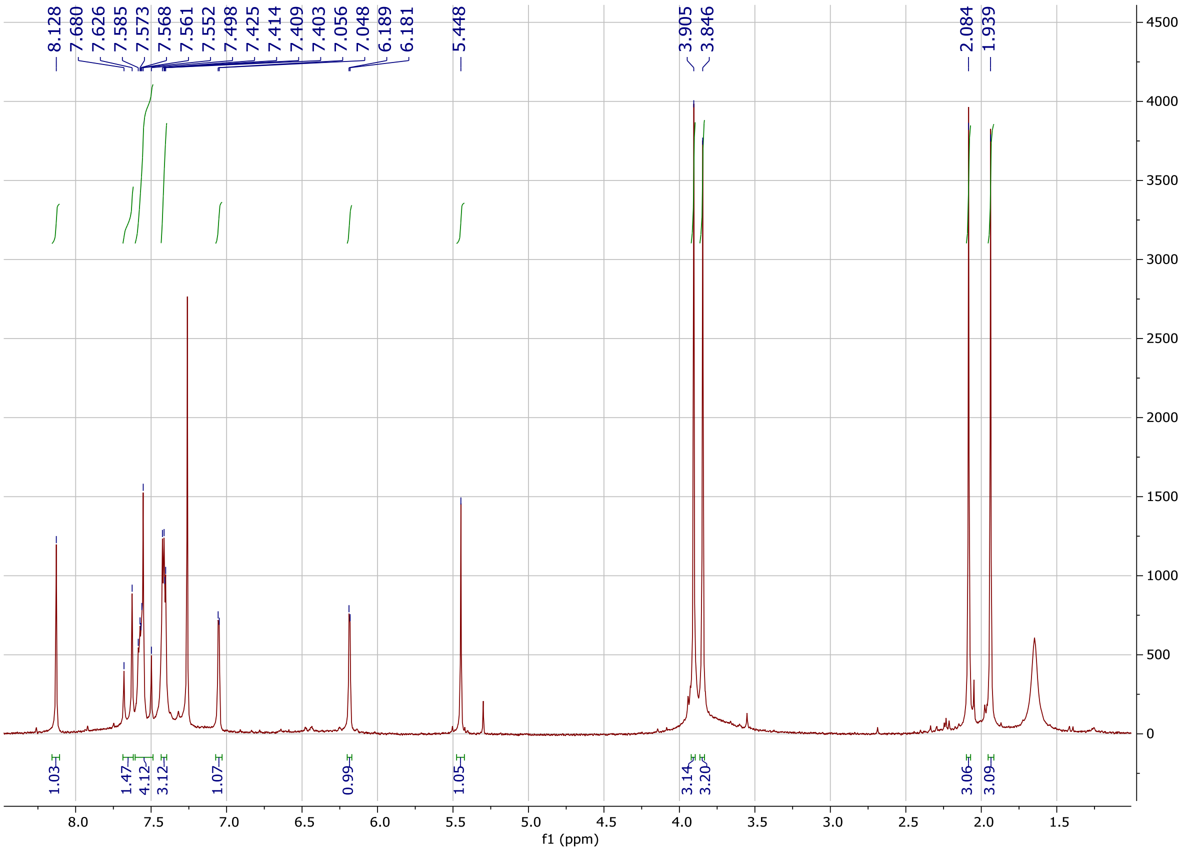

^{1}H-NMR spectrum ($CDCl_3$, 300.13 MHz) of **4a**

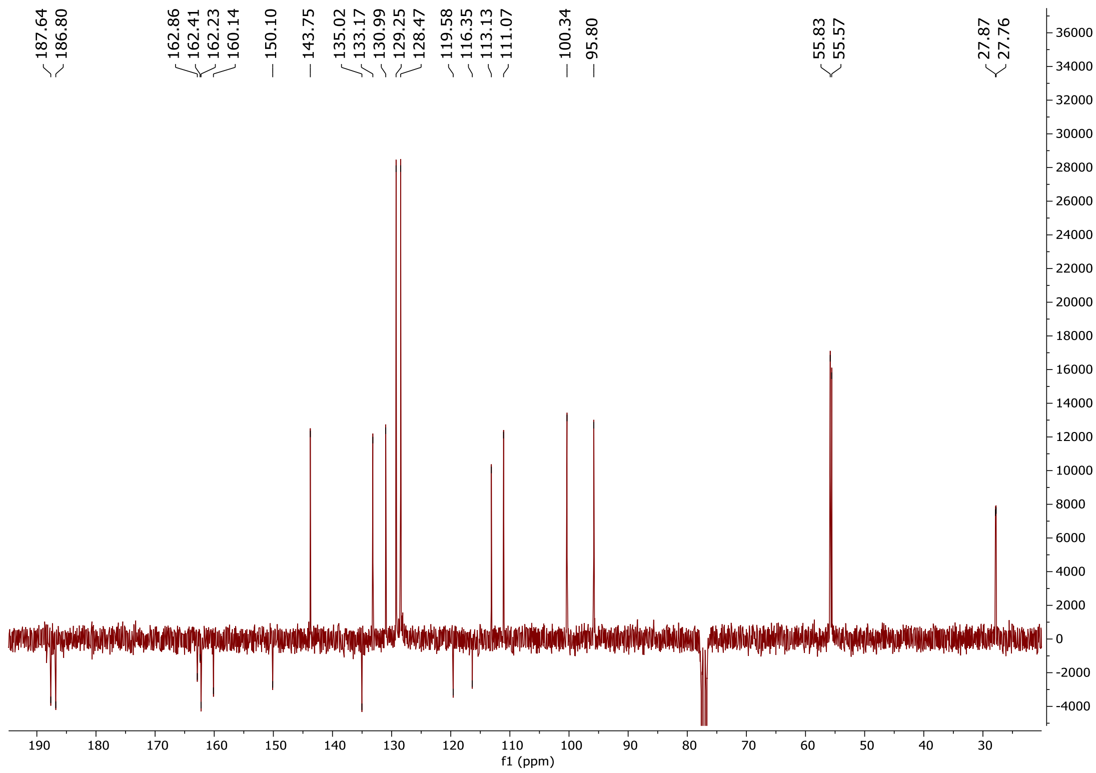

$^{13}C\{^{1}H\}$-(APT) NMR spectrum ($CDCl_3$, 75.47 MHz) of **4a**

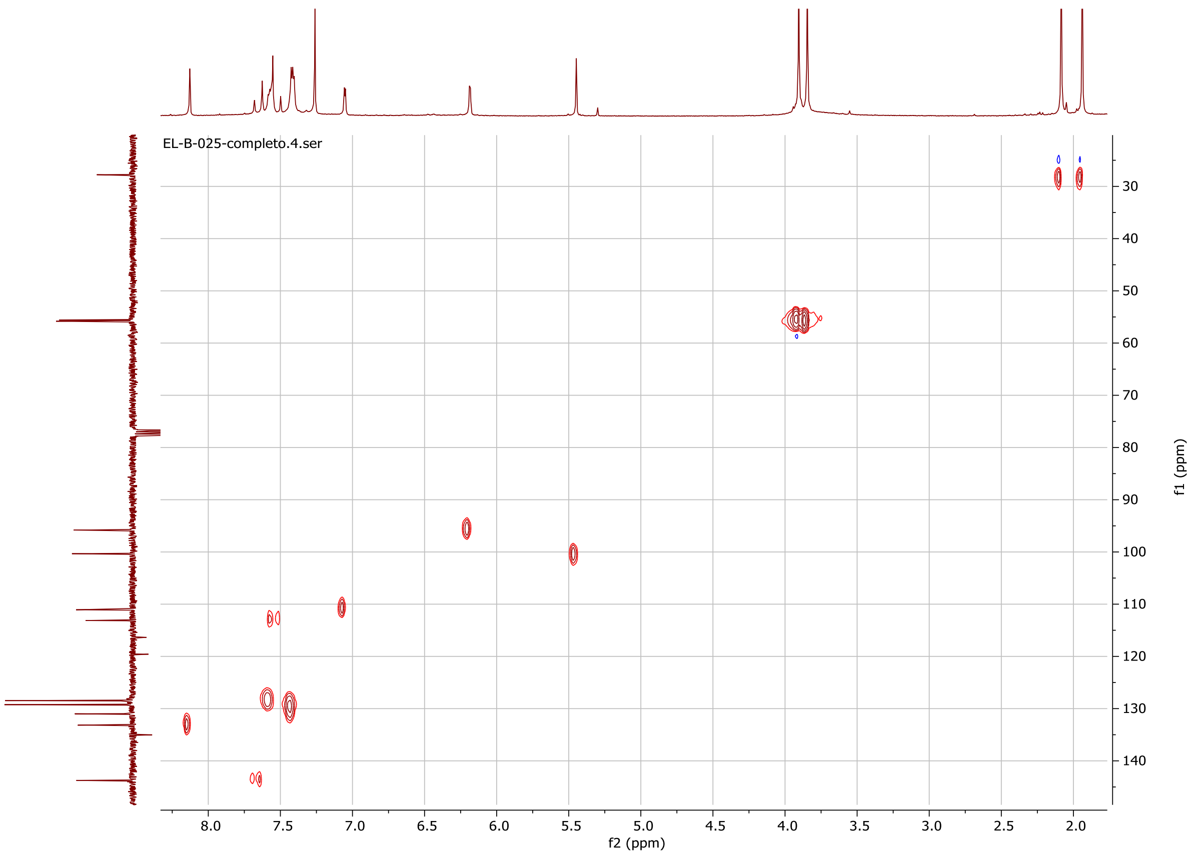

^{1}H-^{13}C HSQC correlation spectrum of **4a**

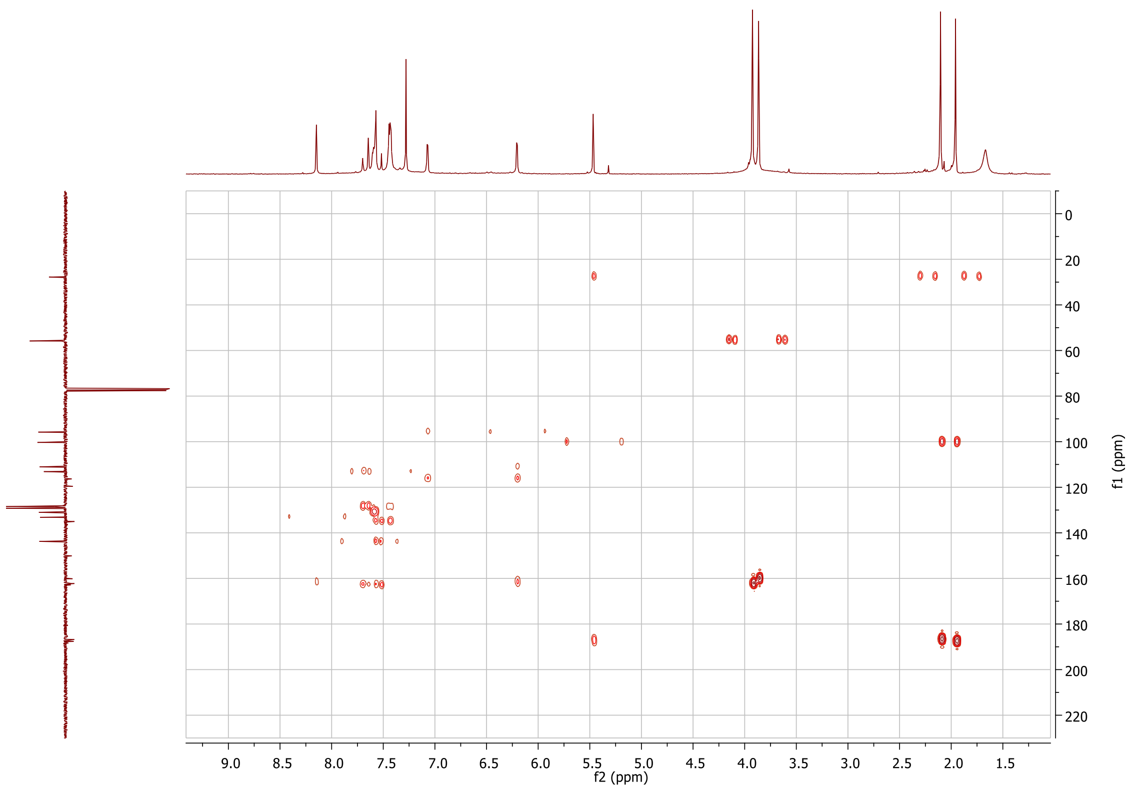

^{1}H-^{13}C HMBC correlation spectrum of **4a**

1.6 Orthopalladated complex **4b**

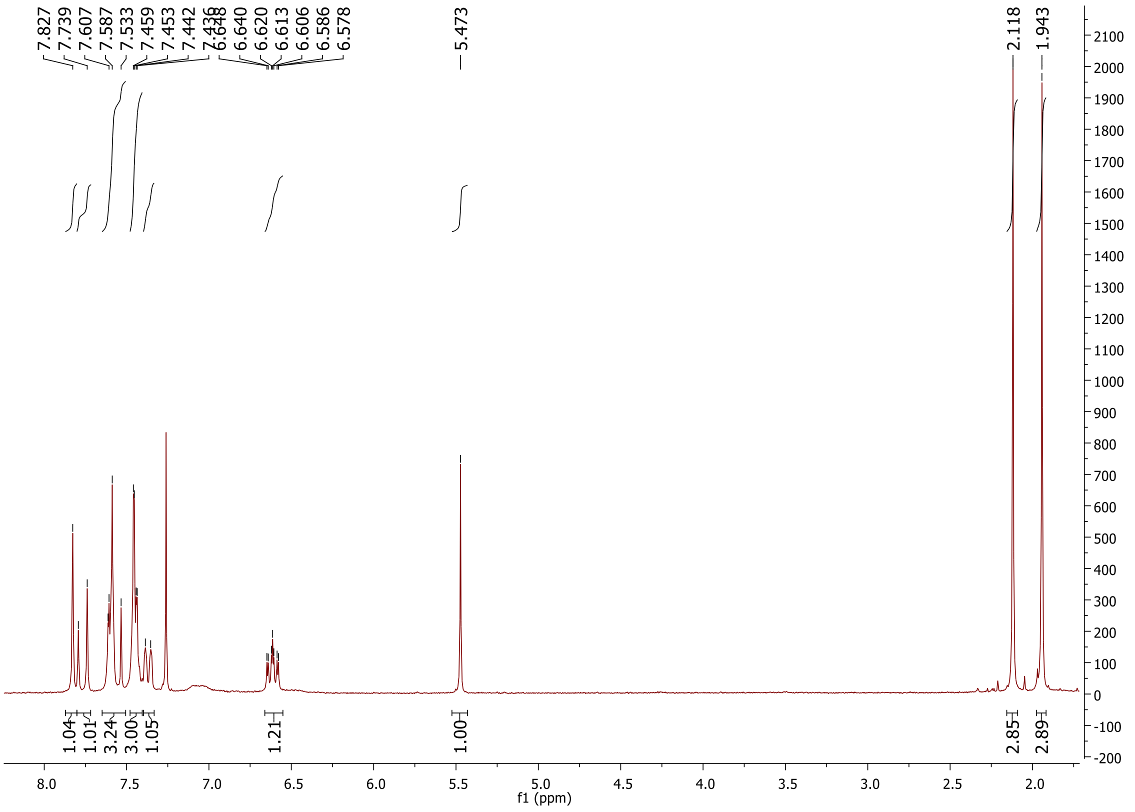

^{1}H-NMR spectrum ($CDCl_3$, 300.13 MHz) of **4b**

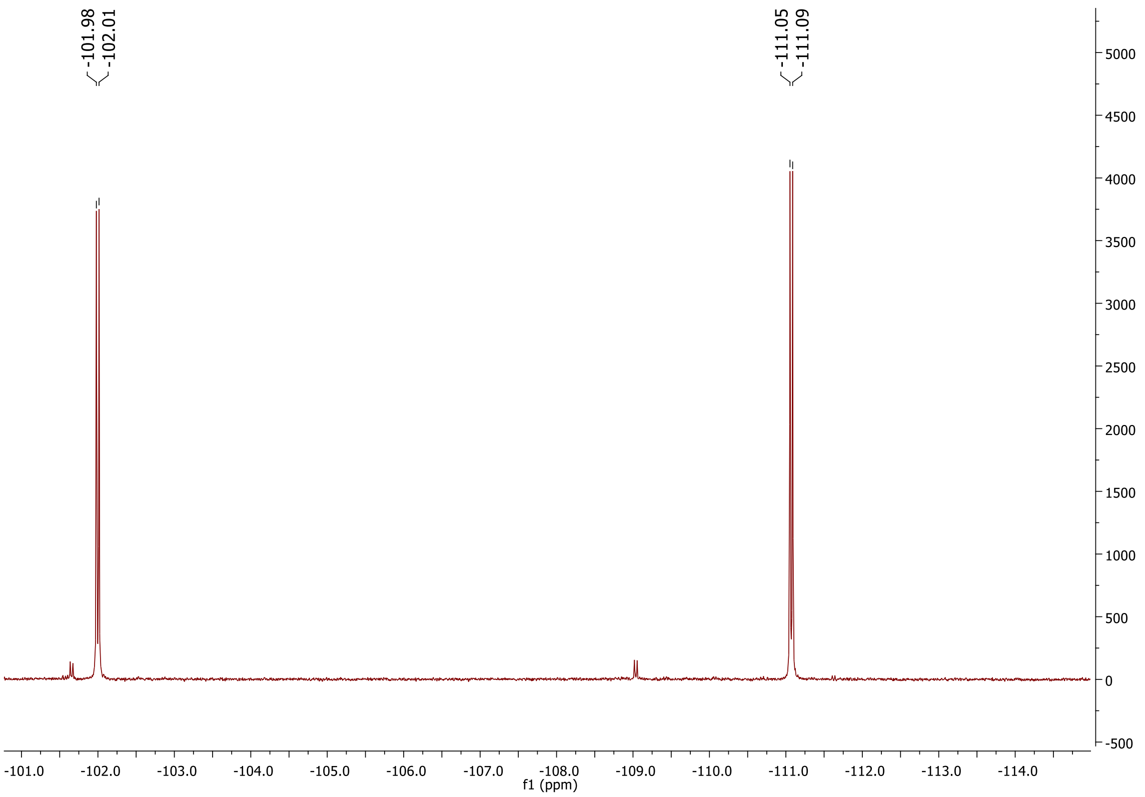

^{19}F{^{1}H}-NMR spectrum ($CDCl_3$, 282.40 MHz) of **4b**

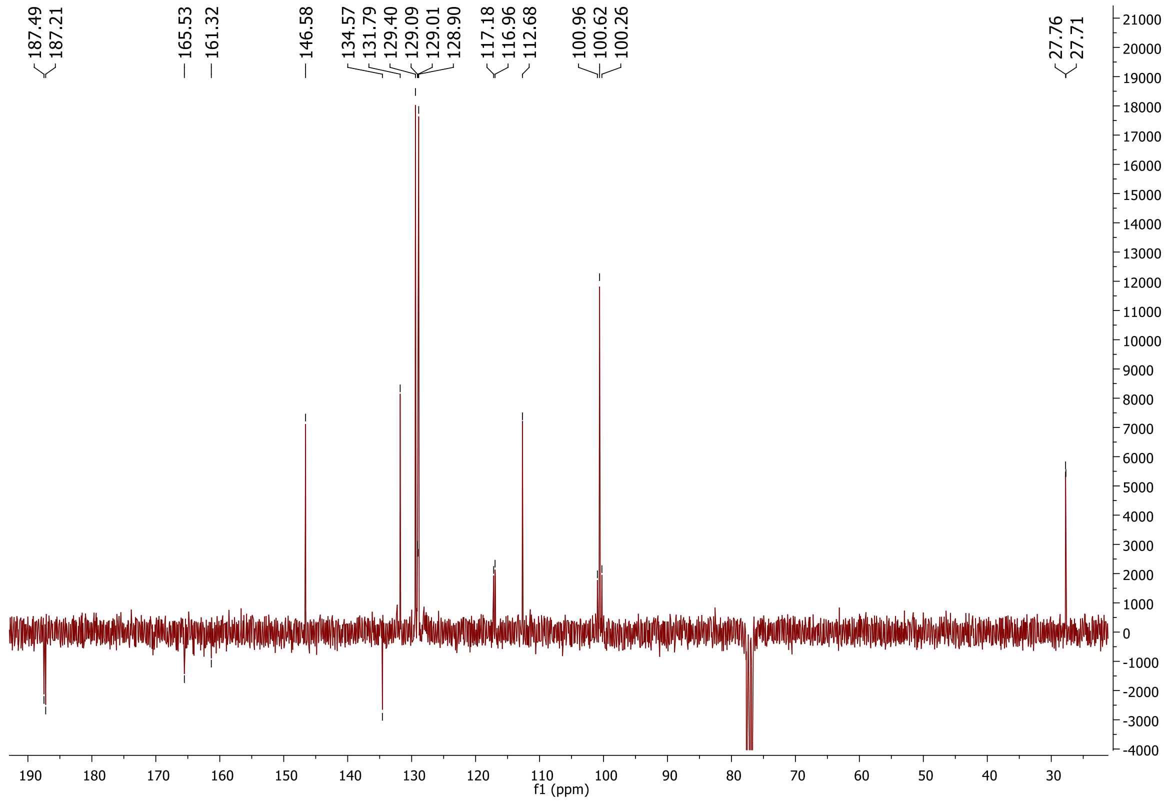

$^{13}C\{^1H\}$-(APT) NMR spectrum ($CDCl_3$, 75.47 MHz) of **4b**

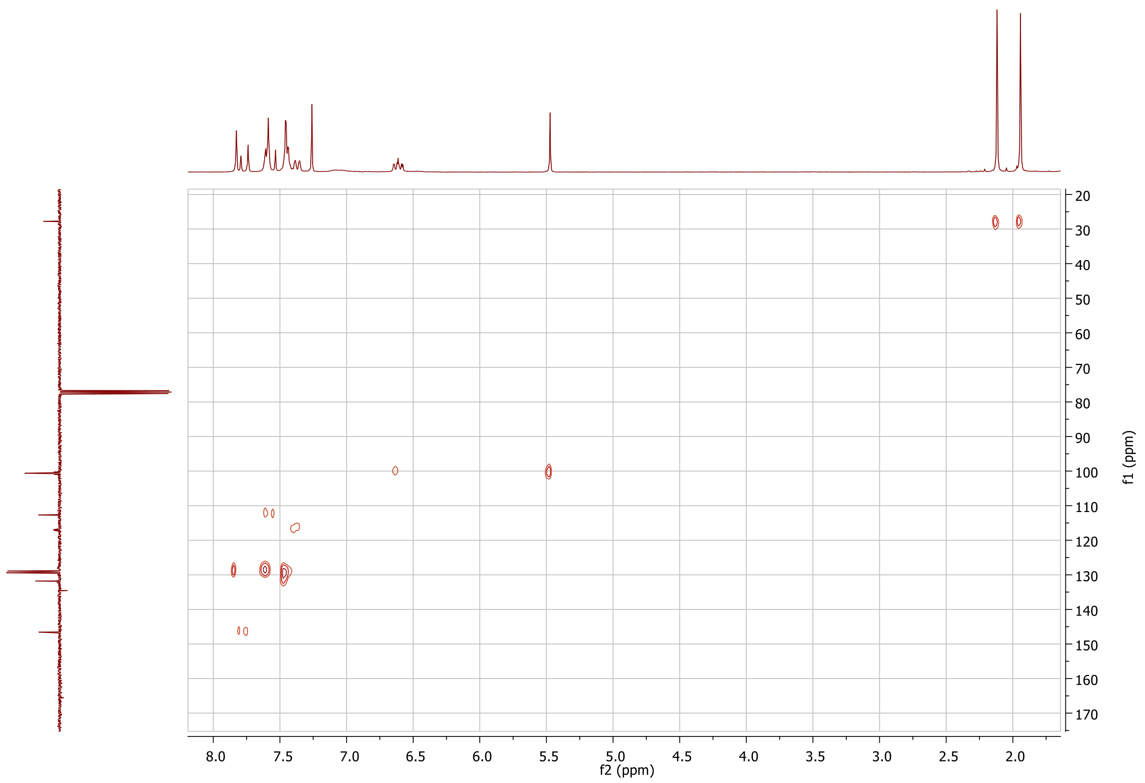

^1H-^{13}C HSQC correlation spectrum of **4b**

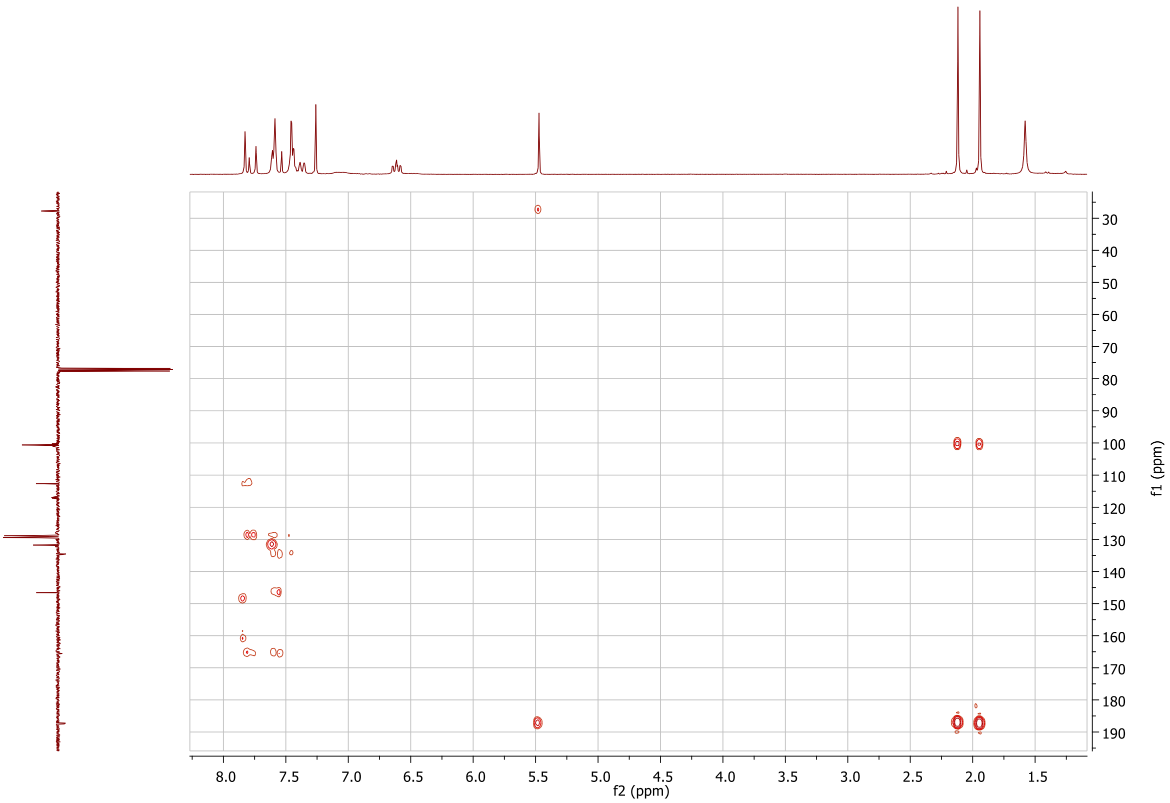

^{1}H-^{13}C HMBC correlation spectrum of **4b**

1.7. Orthopalladated complex **5a**

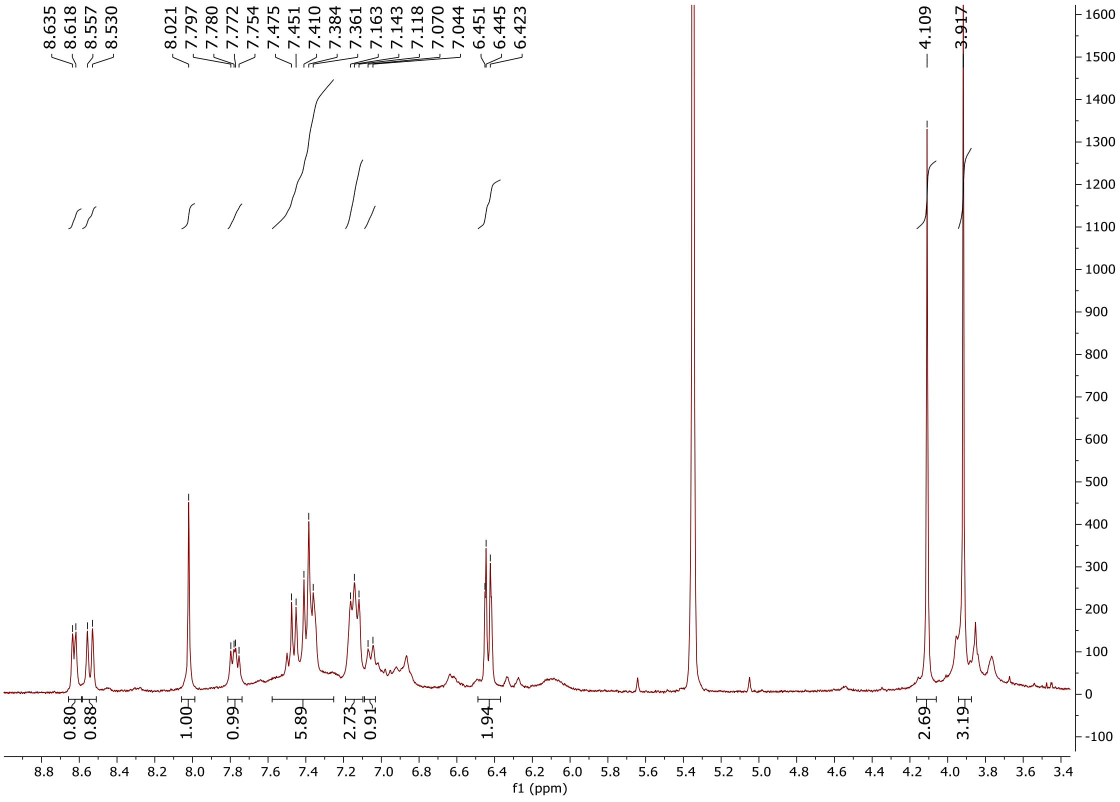

^{1}H-NMR spectrum (CD_2Cl_2, 300.13 MHz) of **5a**

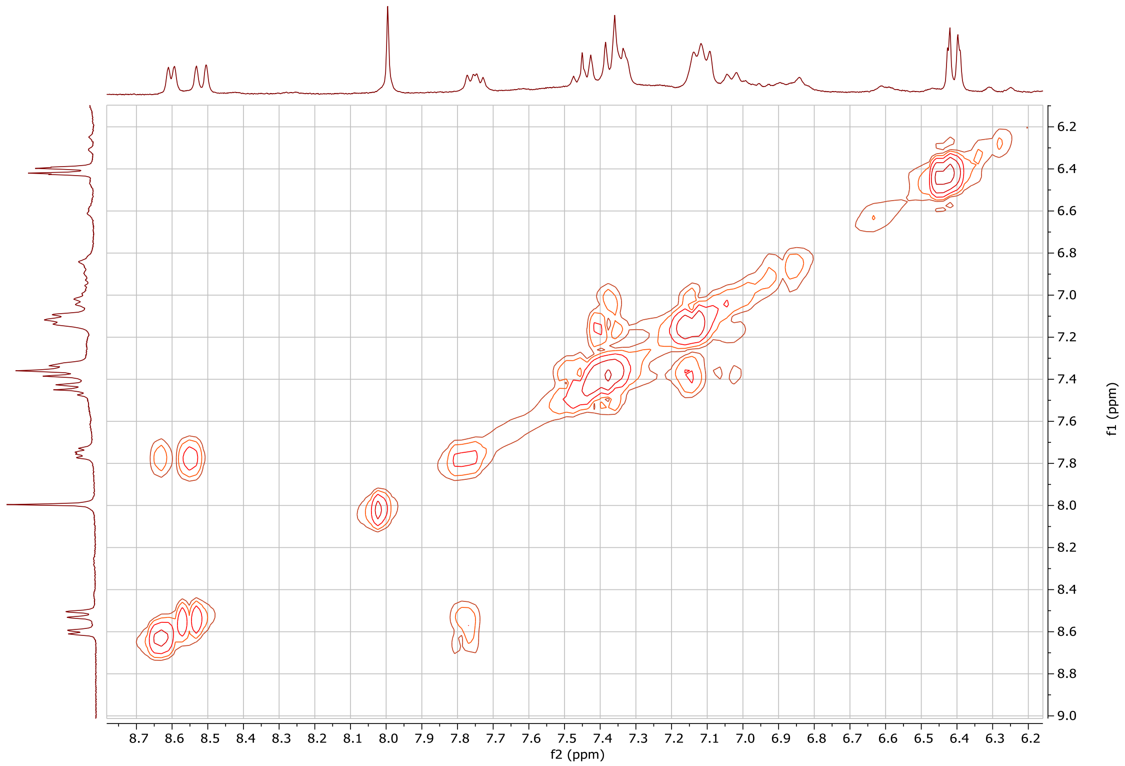

^{1}H-^{1}H COSY correlation spectrum of **5a**

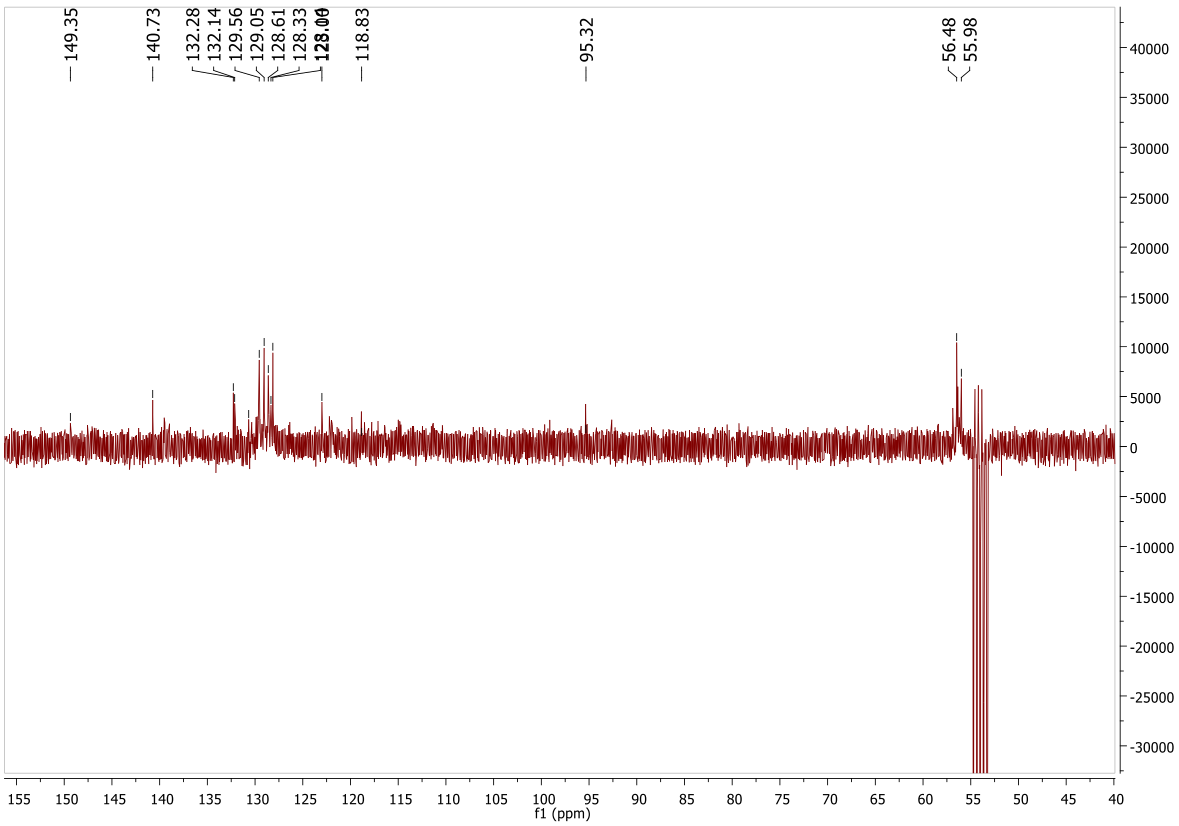

$^{13}C\{^{1}H\}$-(APT) NMR spectrum ($CDCl_3$, 75.47 MHz) of **5a**

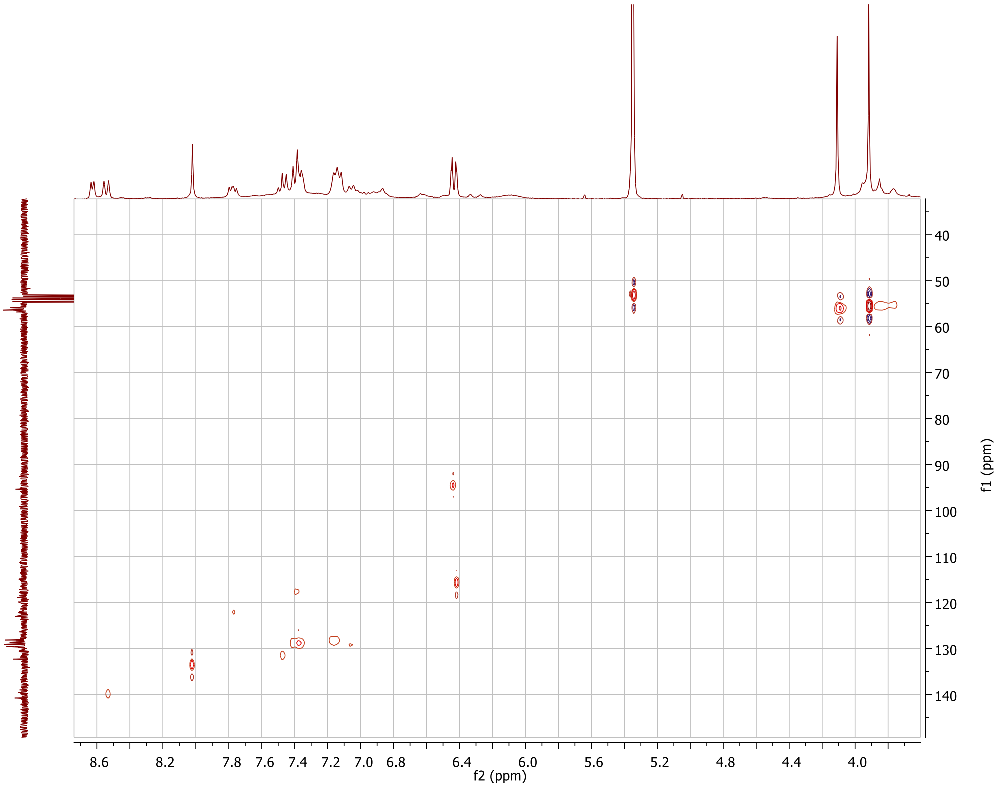

^1H-^{13}C HSQC correlation spectrum of **5a**

1.8. Orthopalladated complex **6a**

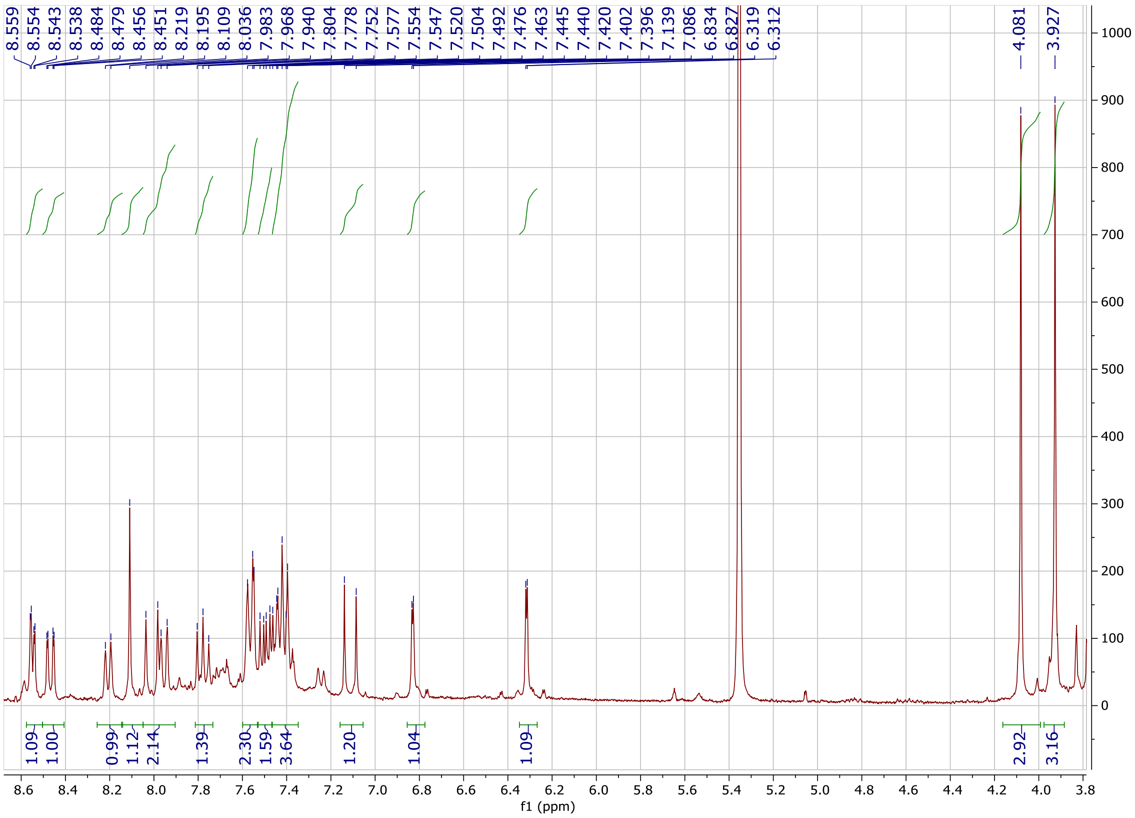

^1H-NMR spectrum (CD_2Cl_2, 300.13 MHz) of **6a**

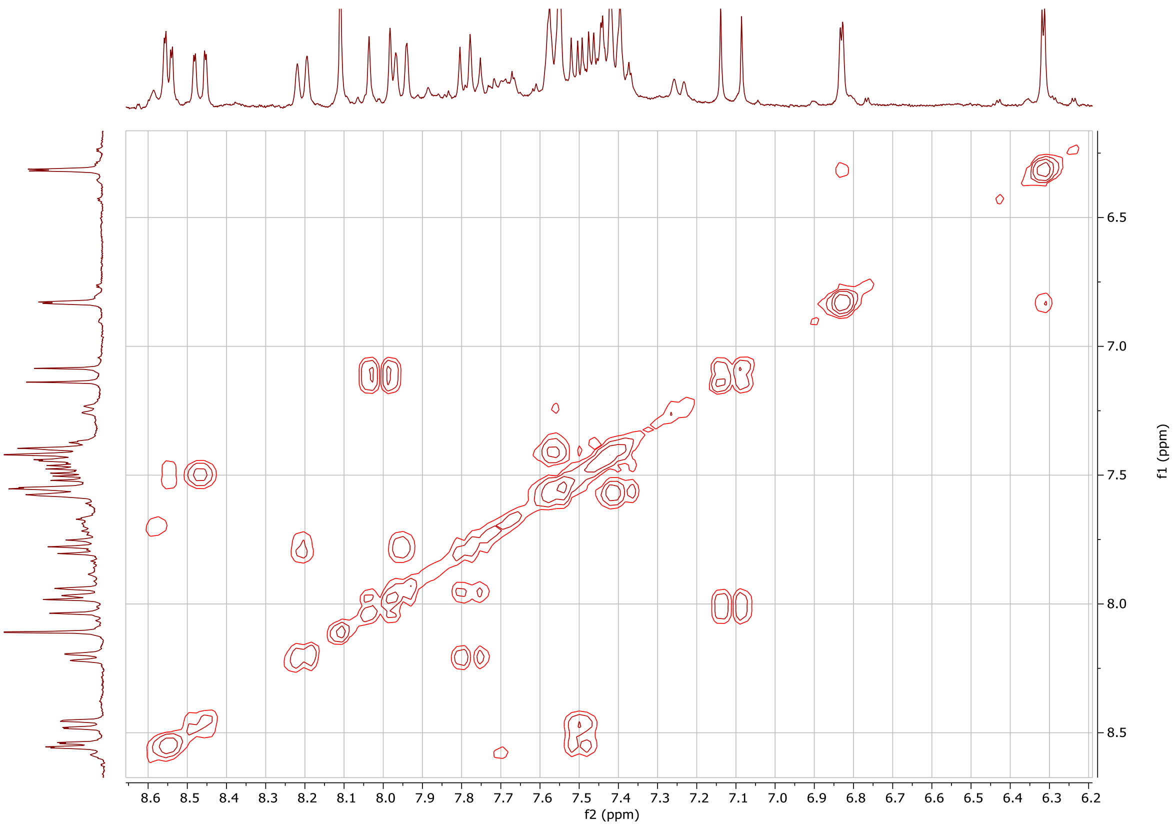

^{1}H-^{1}H COSY correlation spectrum of **6a**

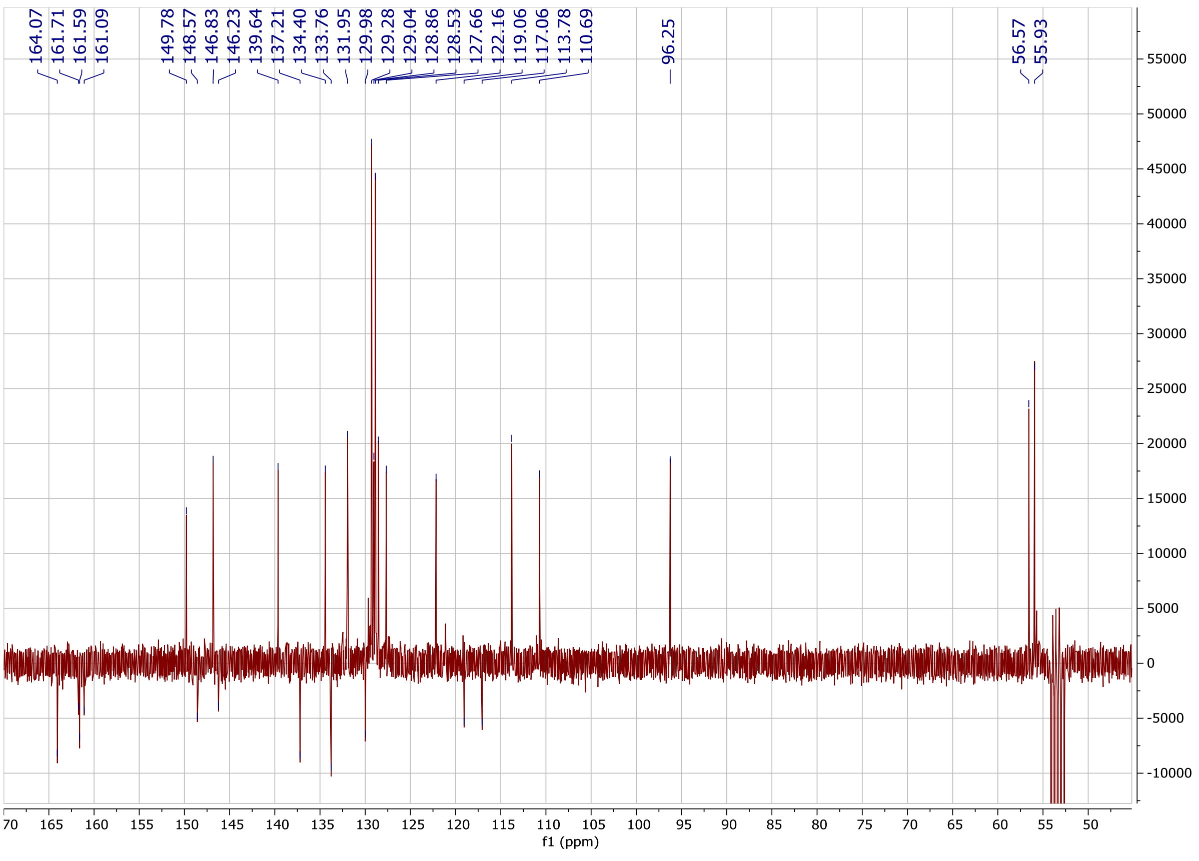

Espectro $^{13}C\{^{1}H\}$-(APT) NMR (CD_2Cl_2, 75.47 MHz) de **6a**

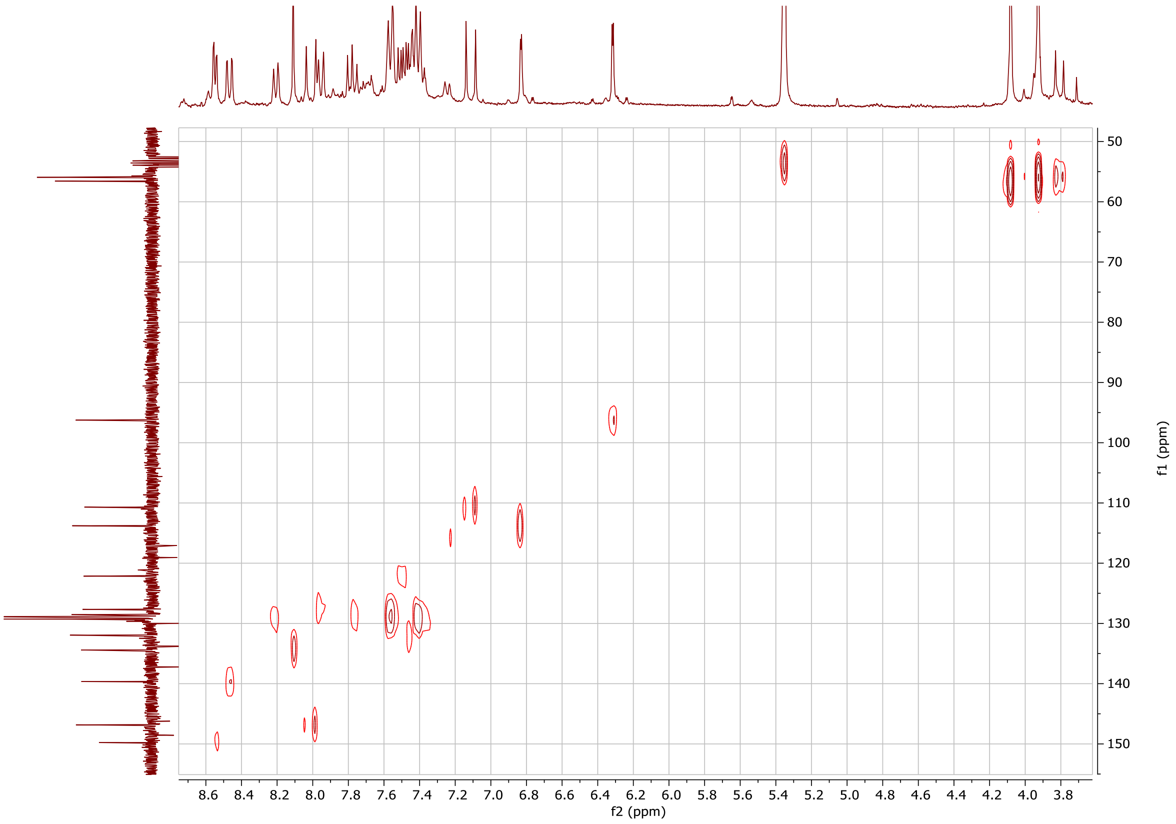

^{1}H-^{13}C HSQC correlation spectrum of **6a**

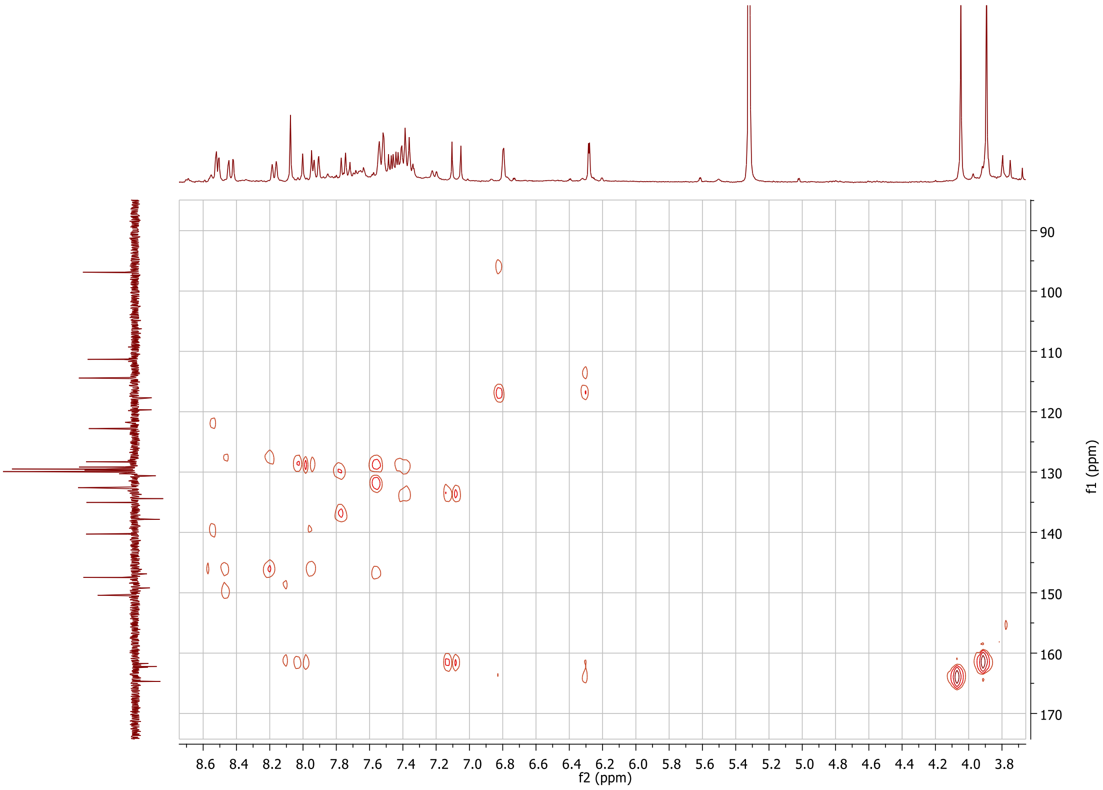

^{1}H-^{13}C HMBC correlation spectrum of **6a**

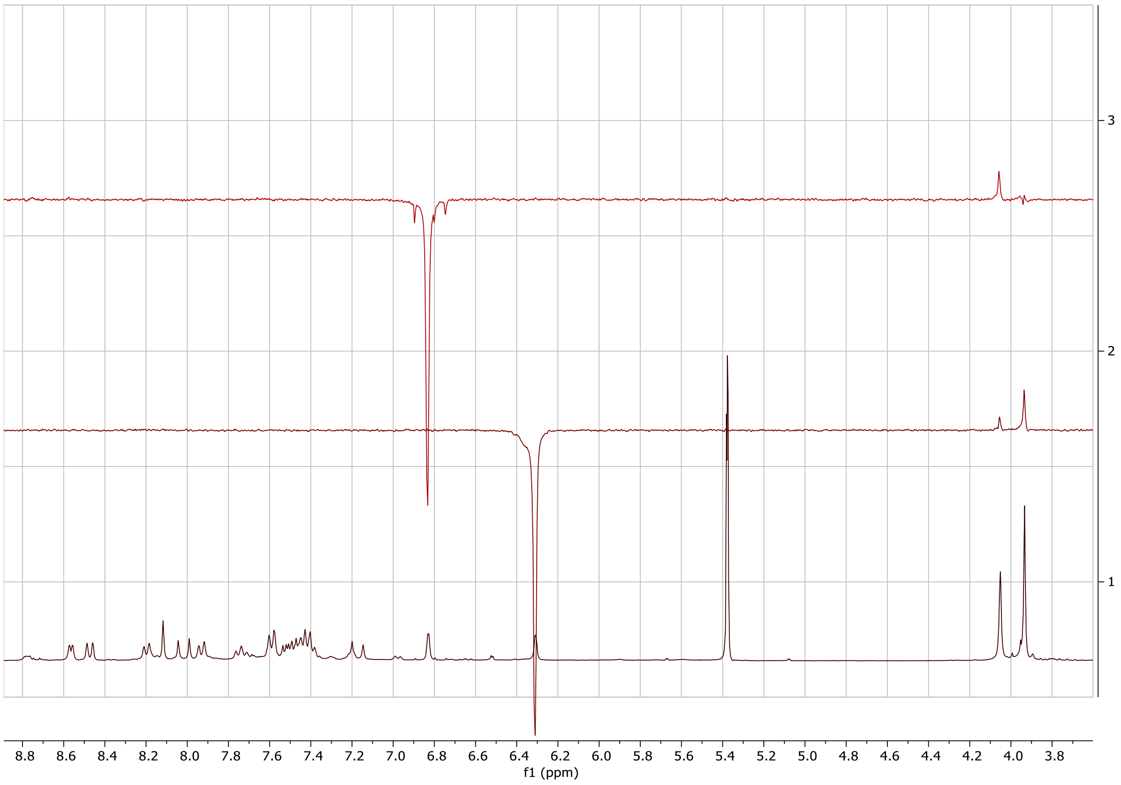

Selective ^{1}H-NOESY of **6a.** 1 (down): off-resonance spectrum; 2 (middle): inversion at 6.31 ppm (H_3); 3 (top): inversion at 6.83 ppm (H_5). NOE is observed only for the signals of the OMe groups.

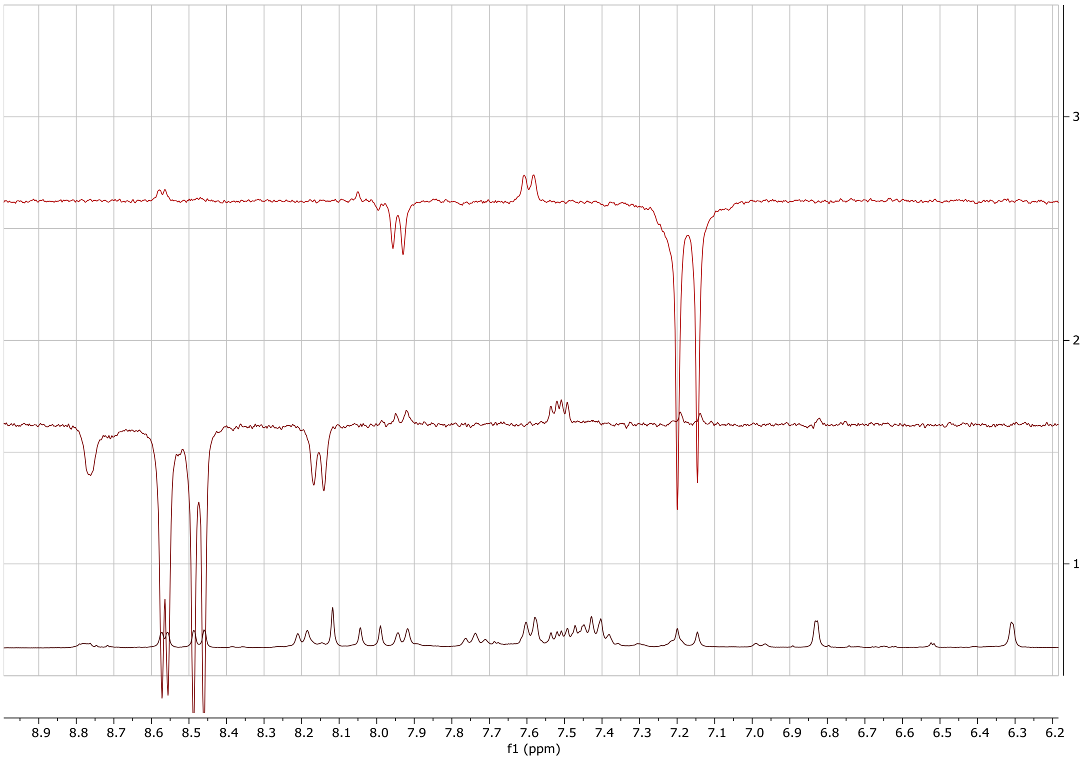

Selective ^{1}H-NOESY of **6a.** 1 (down): off-resonance spectrum; 2 (middle): inversion at 8.54 and 8.46 ppm ($H_{1'}$ and $H_{3'}$) promotes NOE at 7.95 ppm ($H_{5'}$), 7.48 ppm ($H_{2'}$) and 7.11 ppm (olefinic proton); 3 (top): inversion at 7.11 ppm (H_{olef}) promotes NOE at 8.54 ppm ($H_{1'}$) and 7.59 ppm (H_o, C_6H_5).

1.9. Orthopalladated complex **7a**

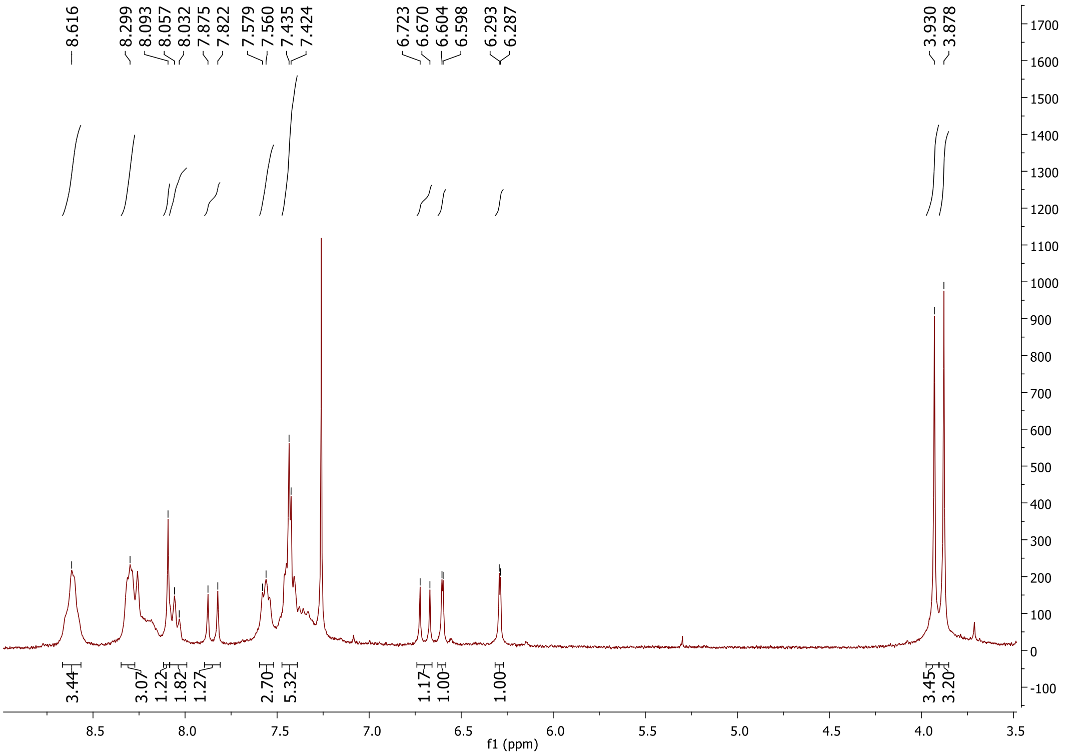

^{1}H-NMR spectrum ($CDCl_3$, 300.13 MHz) of **7a**

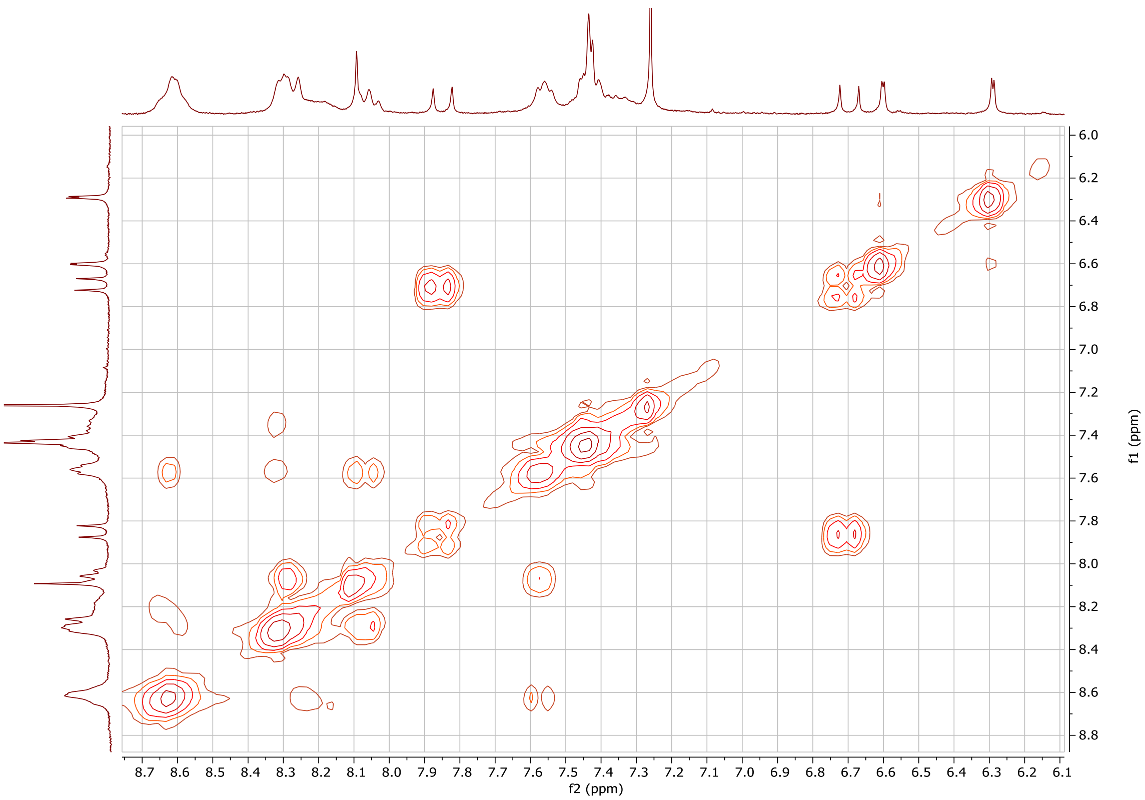

^{1}H-^{1}H COSY correlation spectrum of **7a**

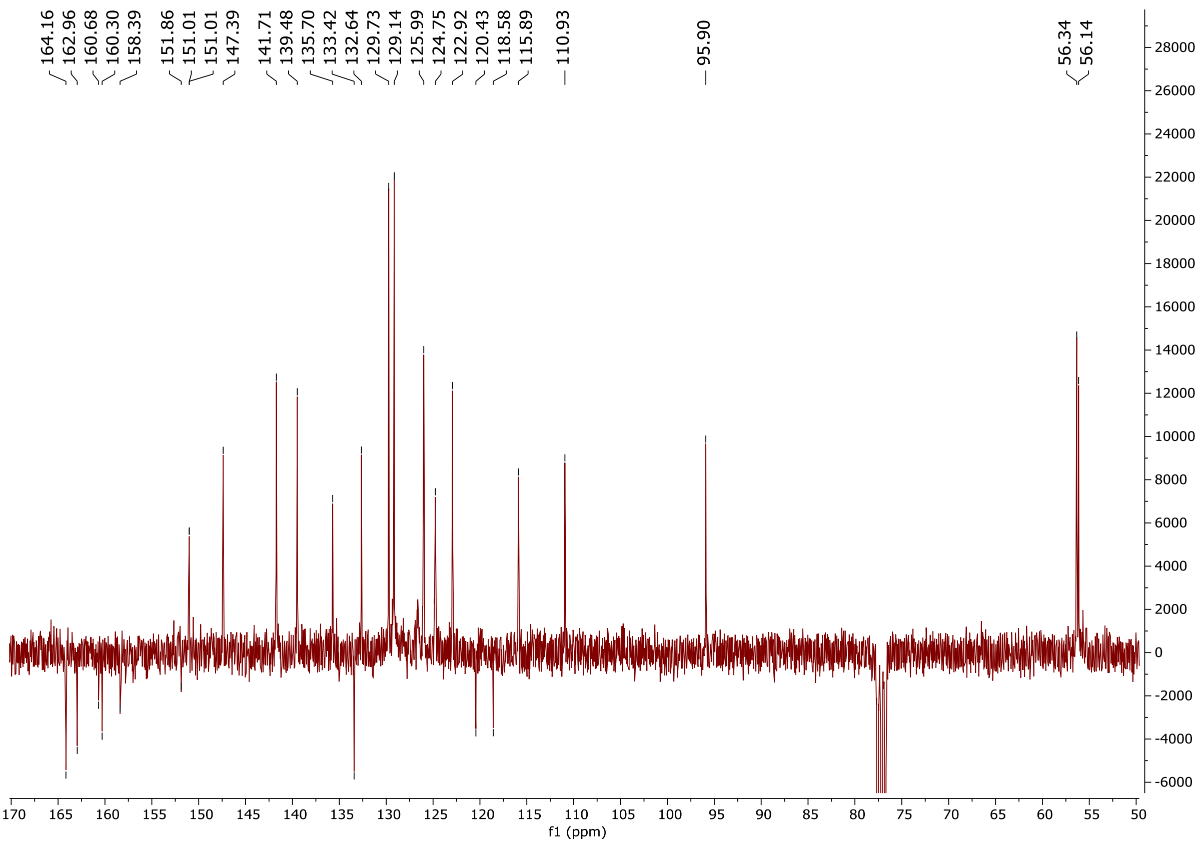

$^{13}C\{^{1}H\}$-(APT) NMR spectrum ($CDCl_3$, 75.47 MHz) of **7a**

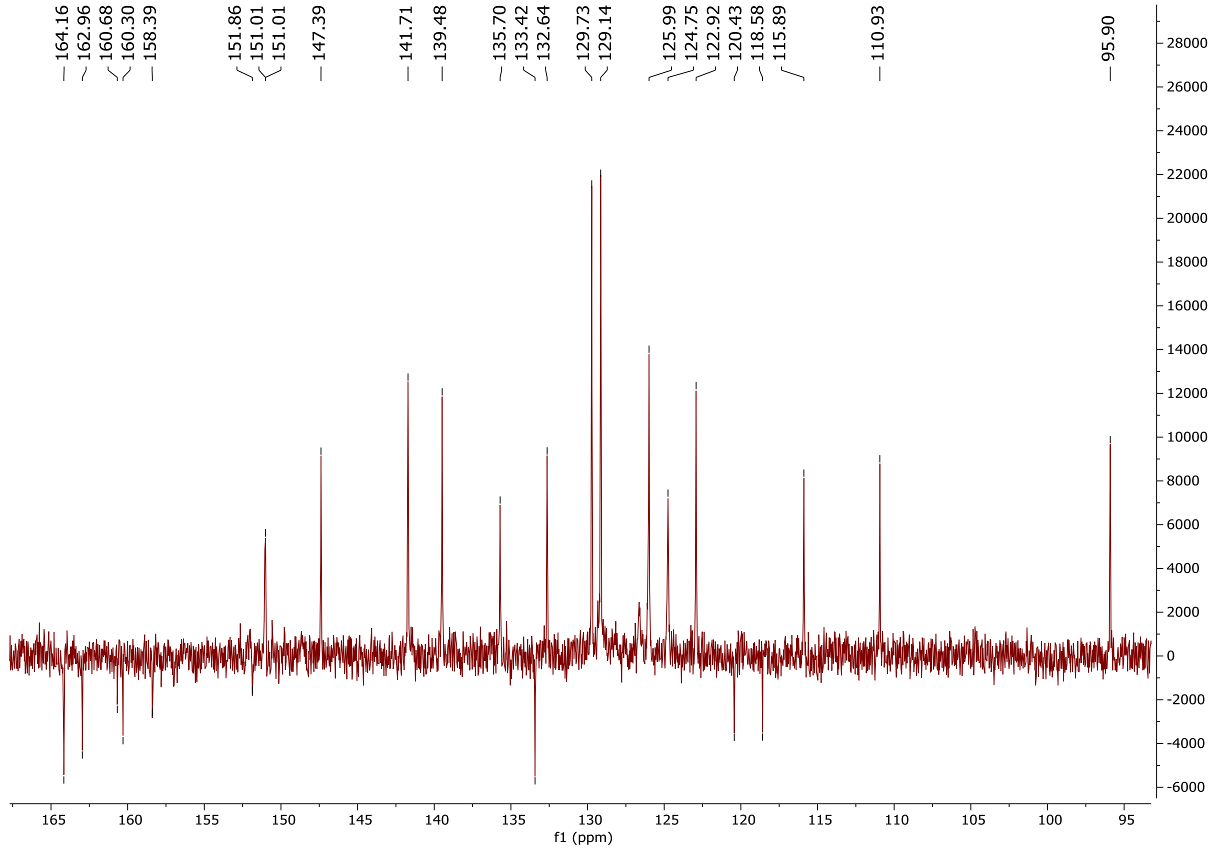

$^{13}C\{^{1}H\}$-(APT) NMR spectrum ($CDCl_3$, 75.47 MHz) of **7a; zoom aromatics**

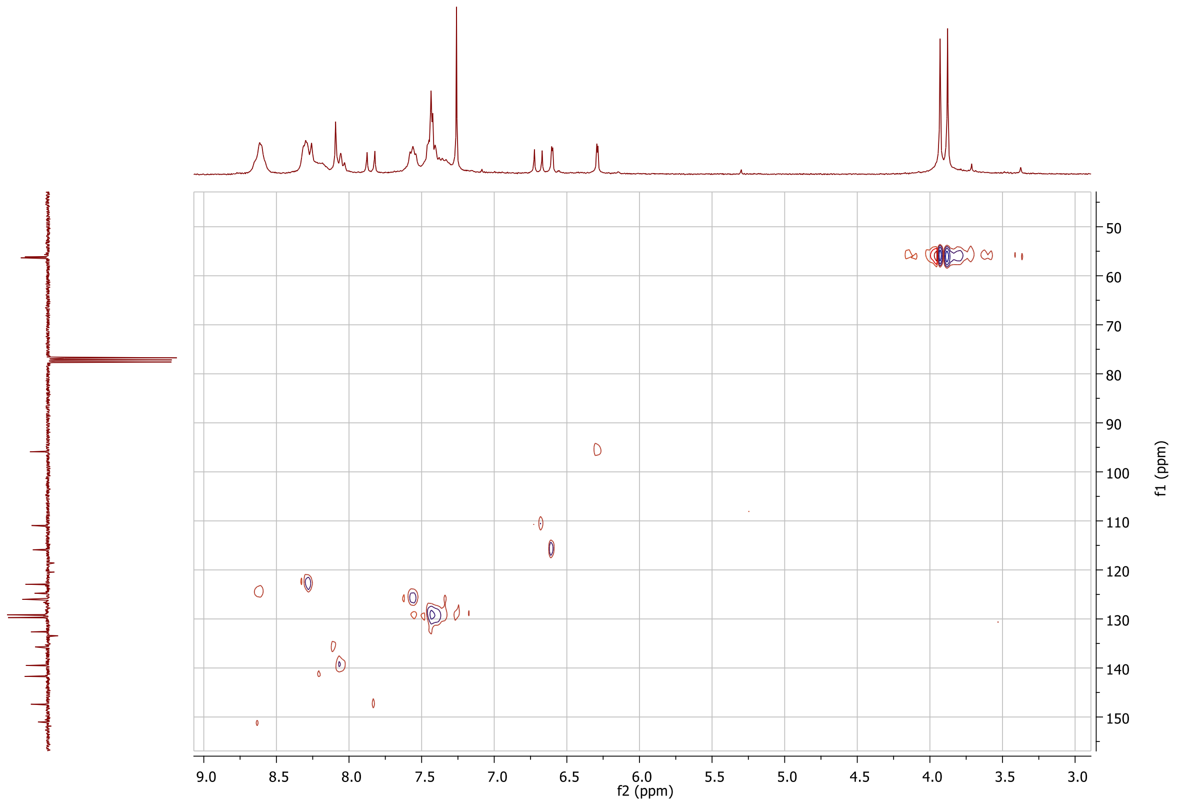

^{1}H-^{13}C HSQC correlation spectrum of **7a**

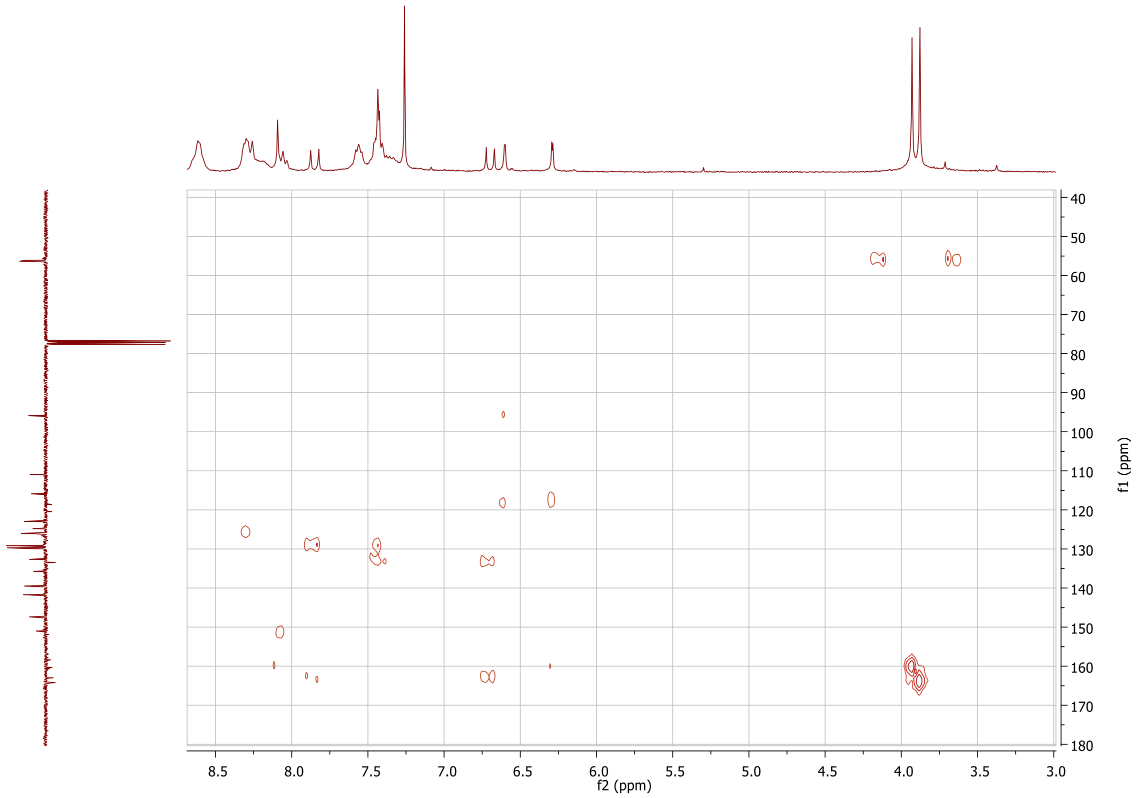

^{1}H-^{13}C HMBC correlation spectrum of **7a**

1.10. Orthopalladated complex **8a**

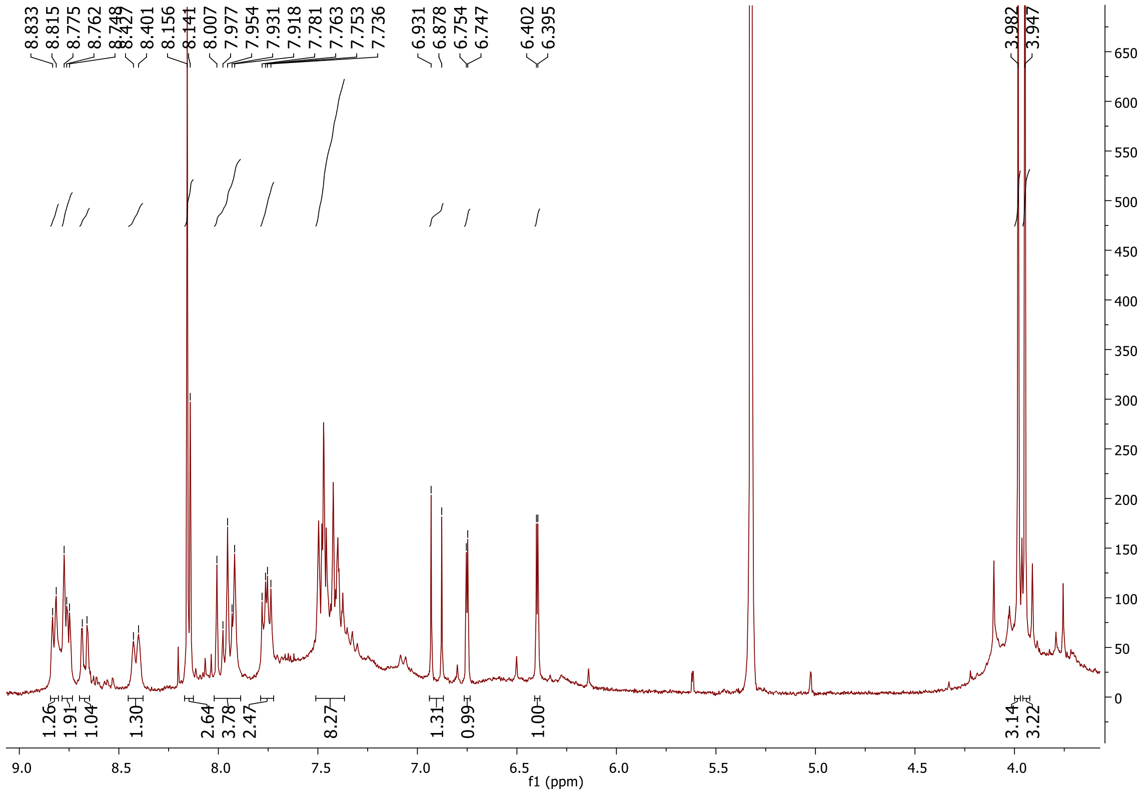

^{1}H-NMR spectrum (CD_2Cl_2, 300.13 MHz) of **8a**

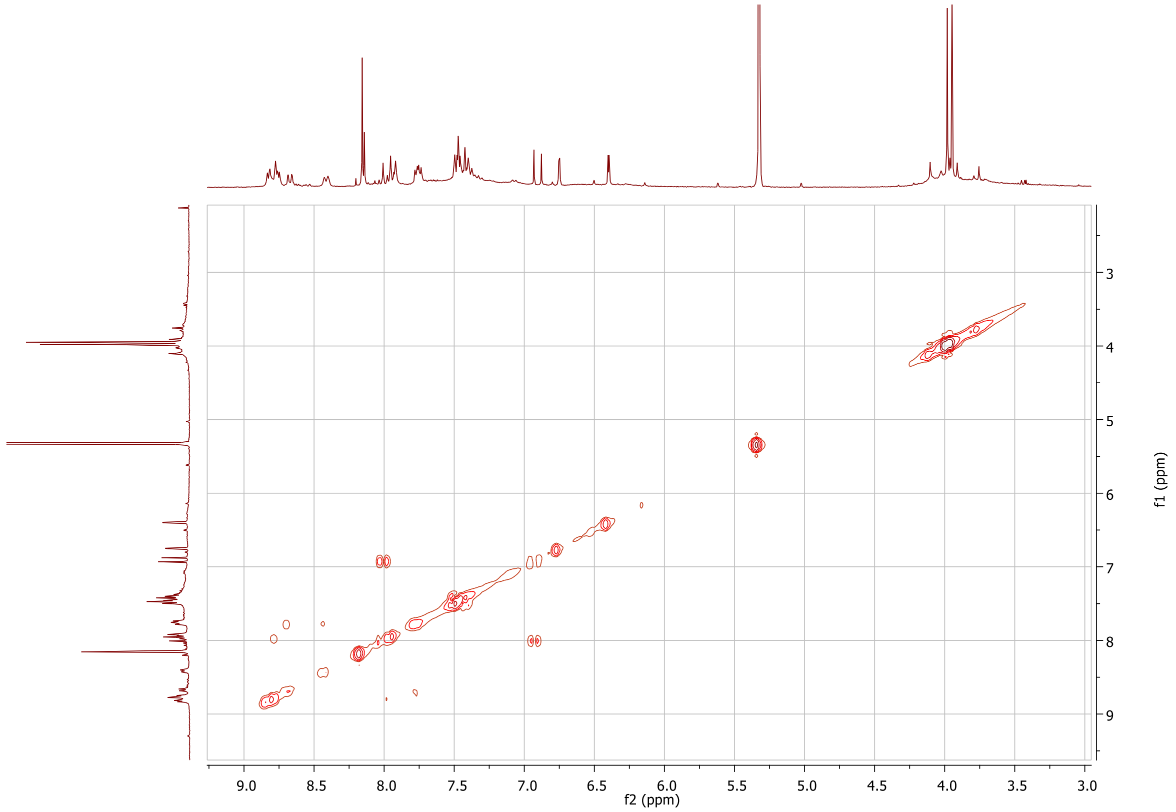

^{1}H-^{1}H COSY correlation spectrum of **8a**

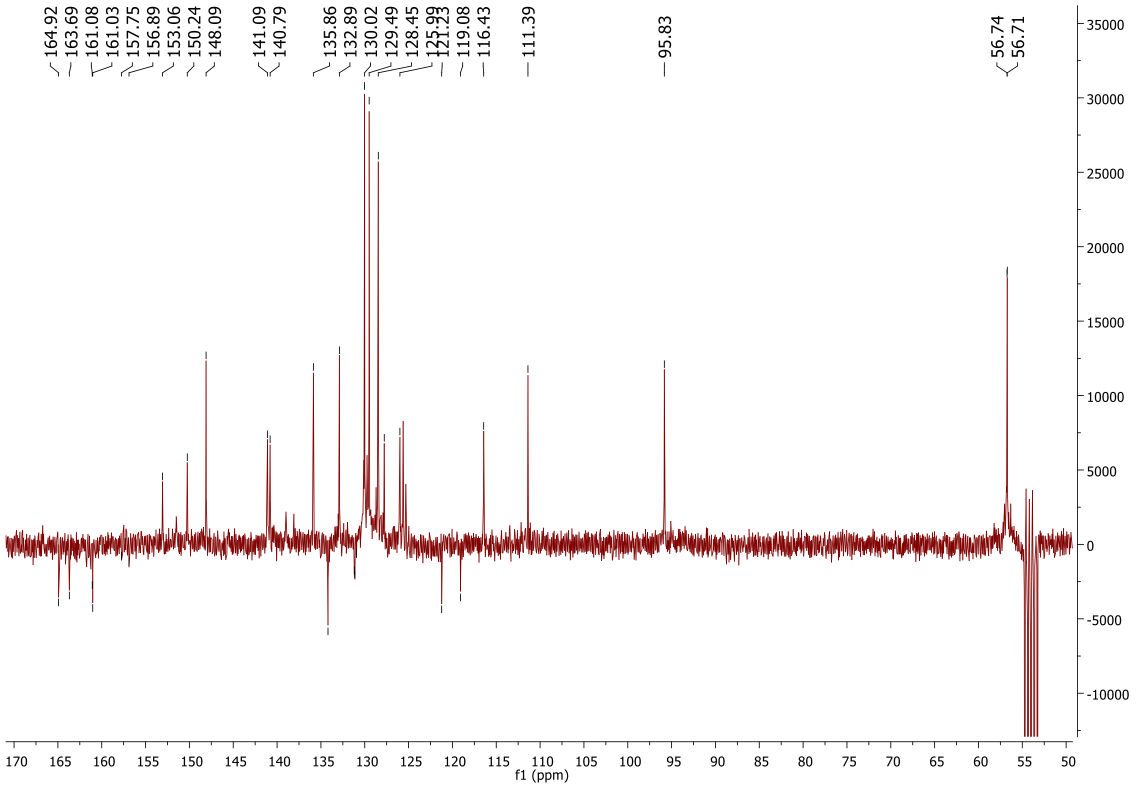

Espectro $^{13}C\{^{1}H\}$-(APT) NMR (CD_2Cl_2, 75.47 MHz) de **8a**

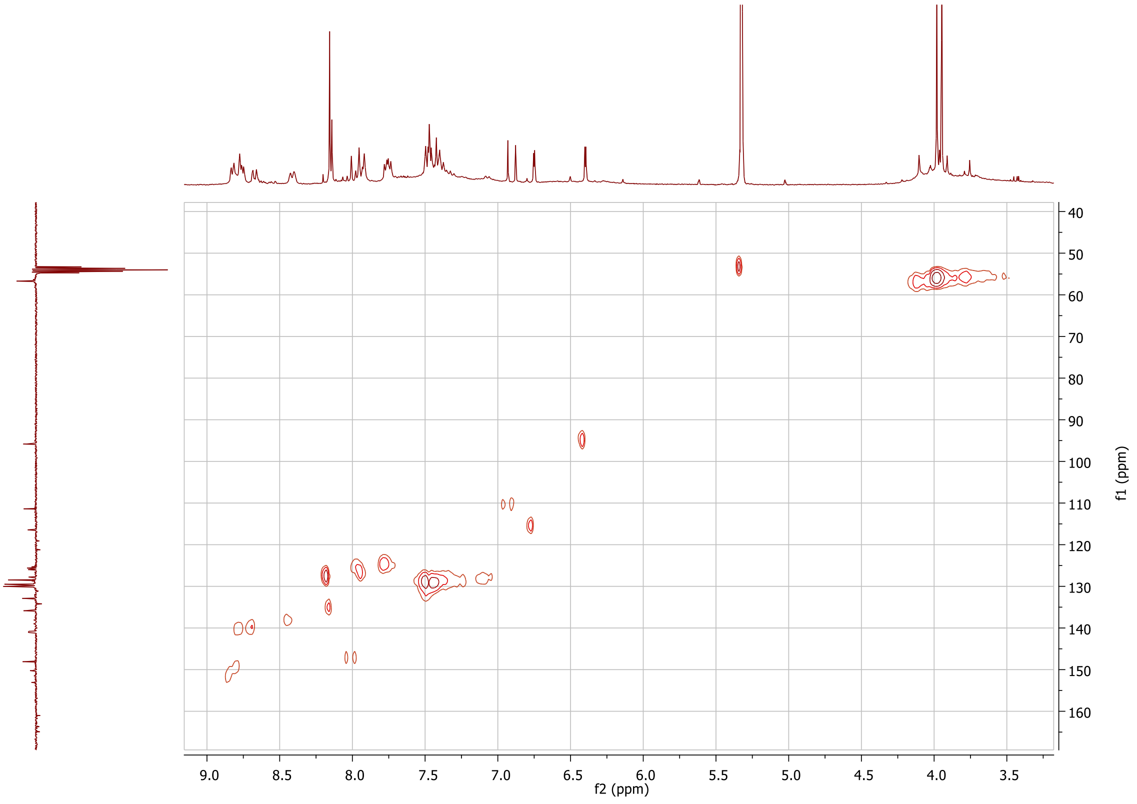

^{1}H-^{13}C HSQC correlation spectrum of **8a**

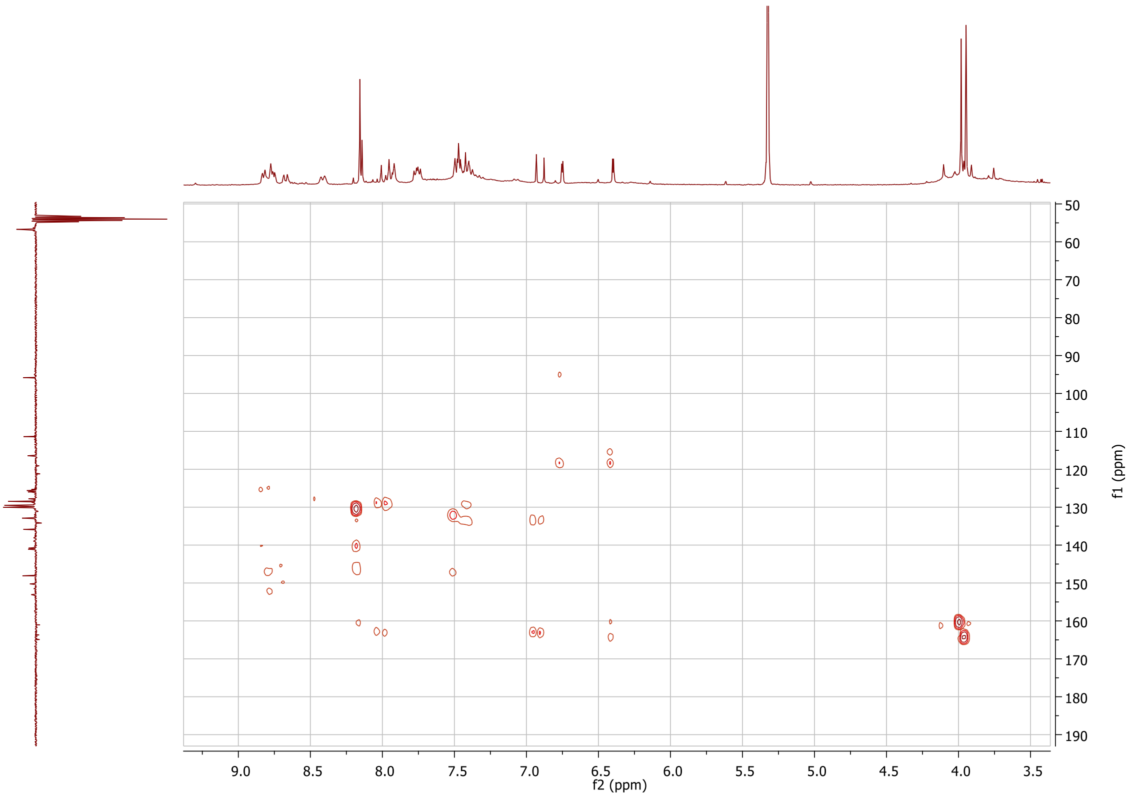

^1H-^{13}C HMBC correlation spectrum of **8a**

2. IR SPECTRA

2.1. <u>IR spectrum of **2a**</u>

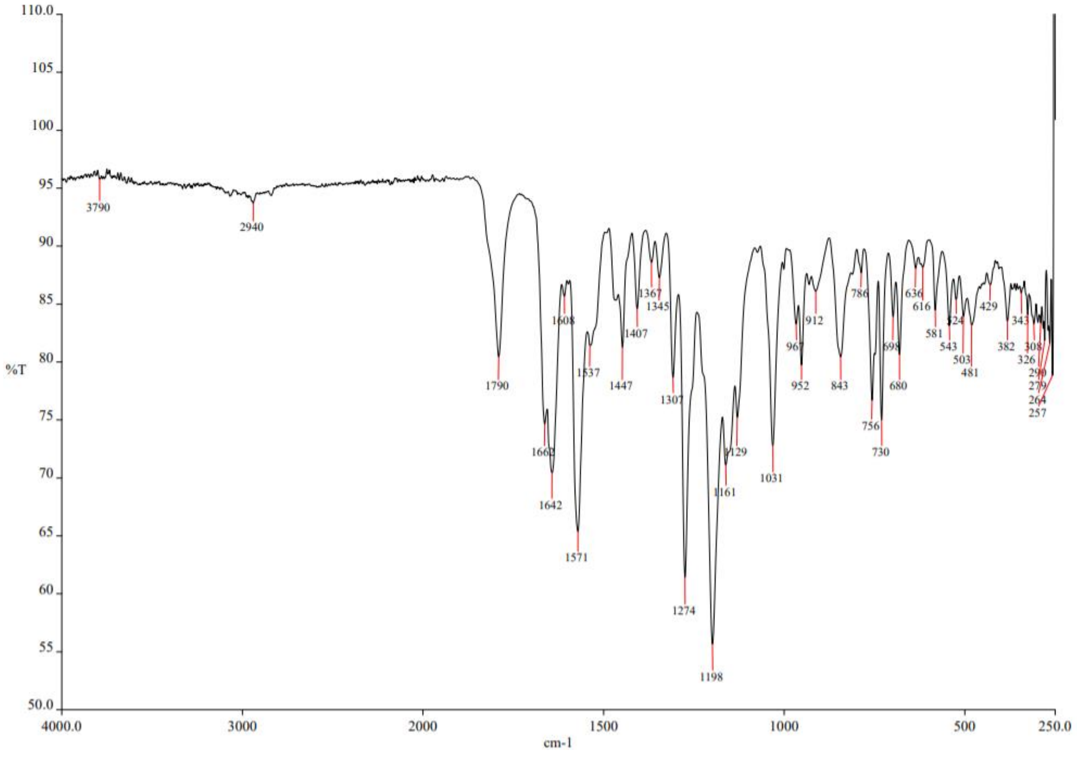

2.2. IR spectrum of **2b**

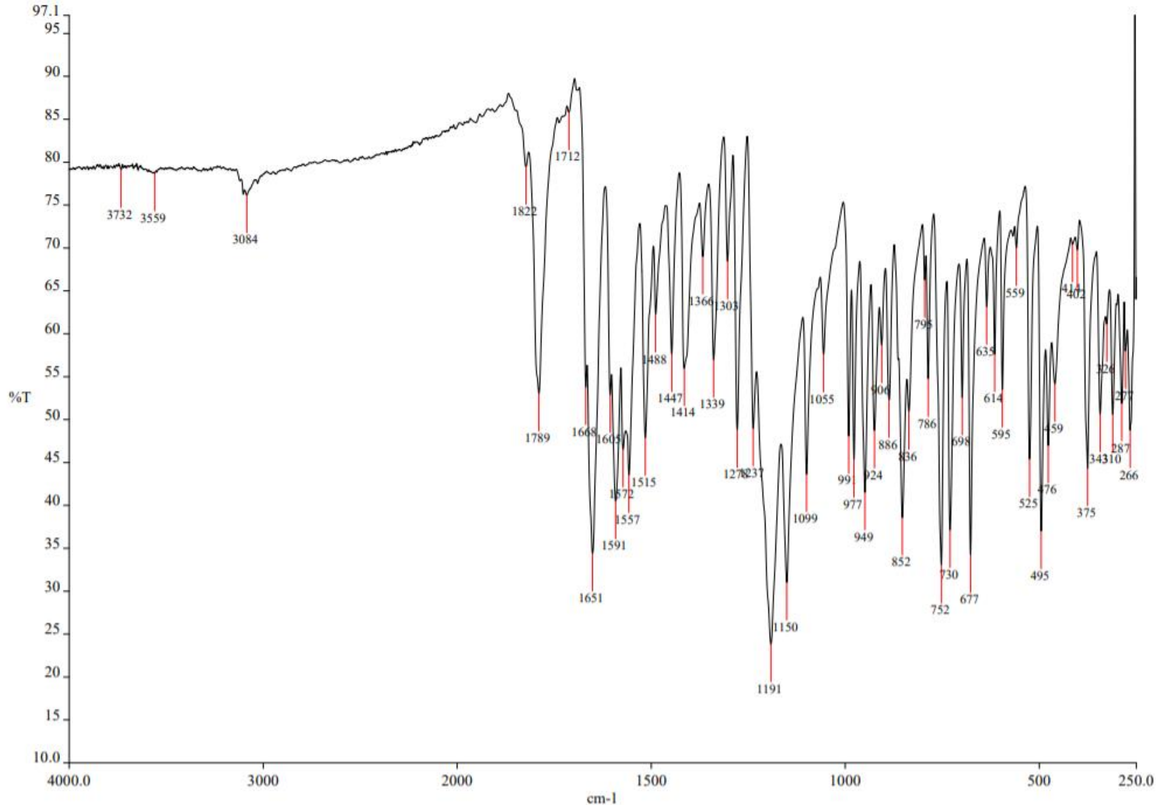

2.3. IR spectrum of **3a**

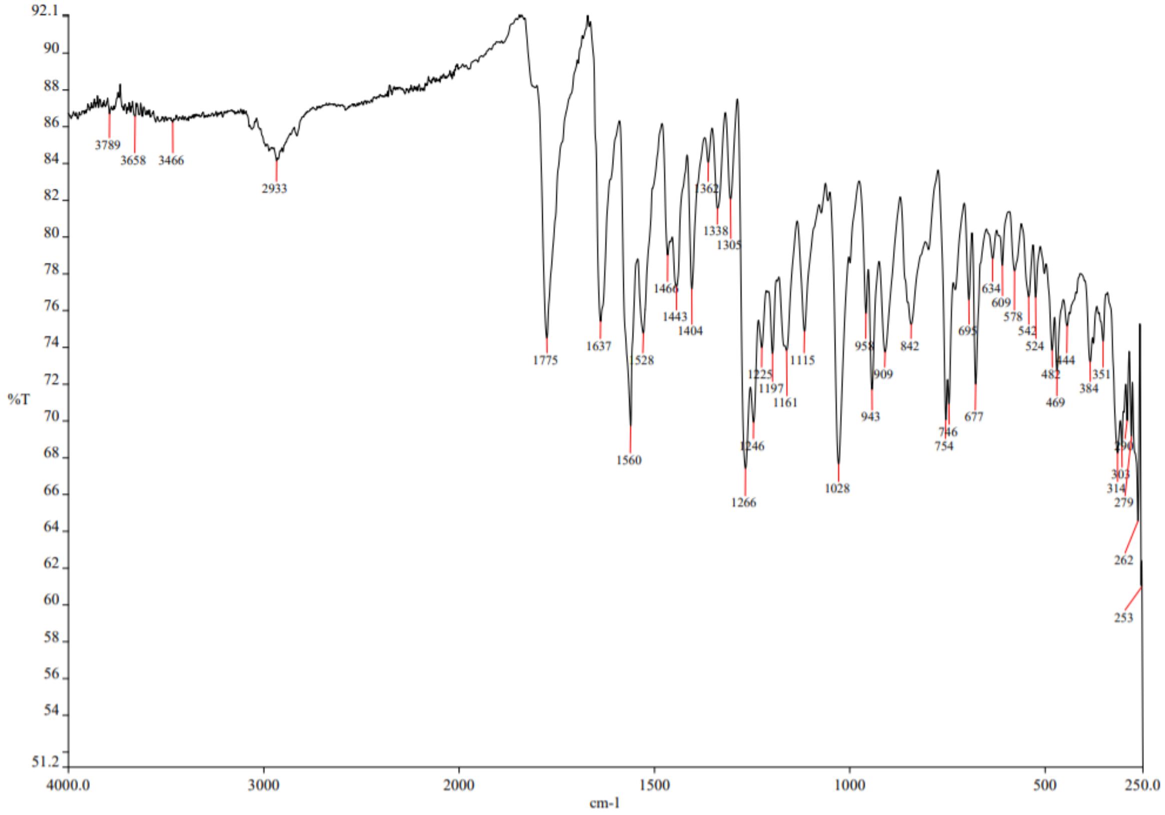

2.4. IR spectrum of **3b**

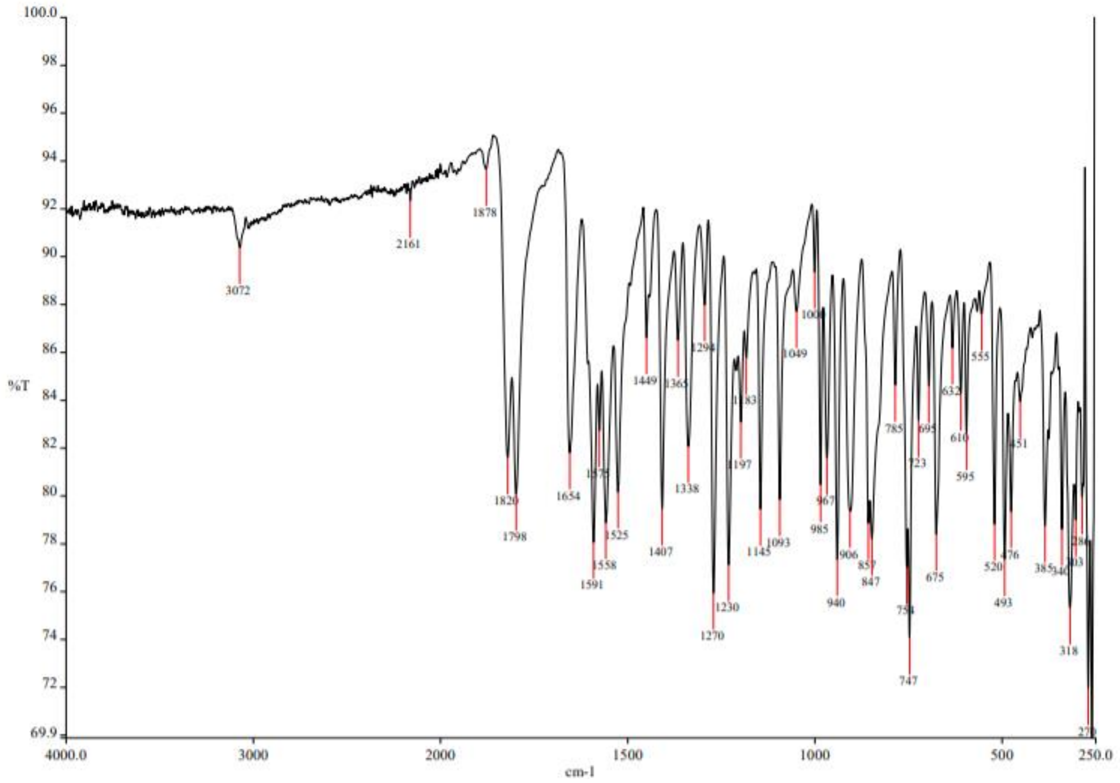

3. ABSORPTION UV-Vis SPECTRA

3.1. Comparison of the absorption spectra of 1a-4a-6a-7a

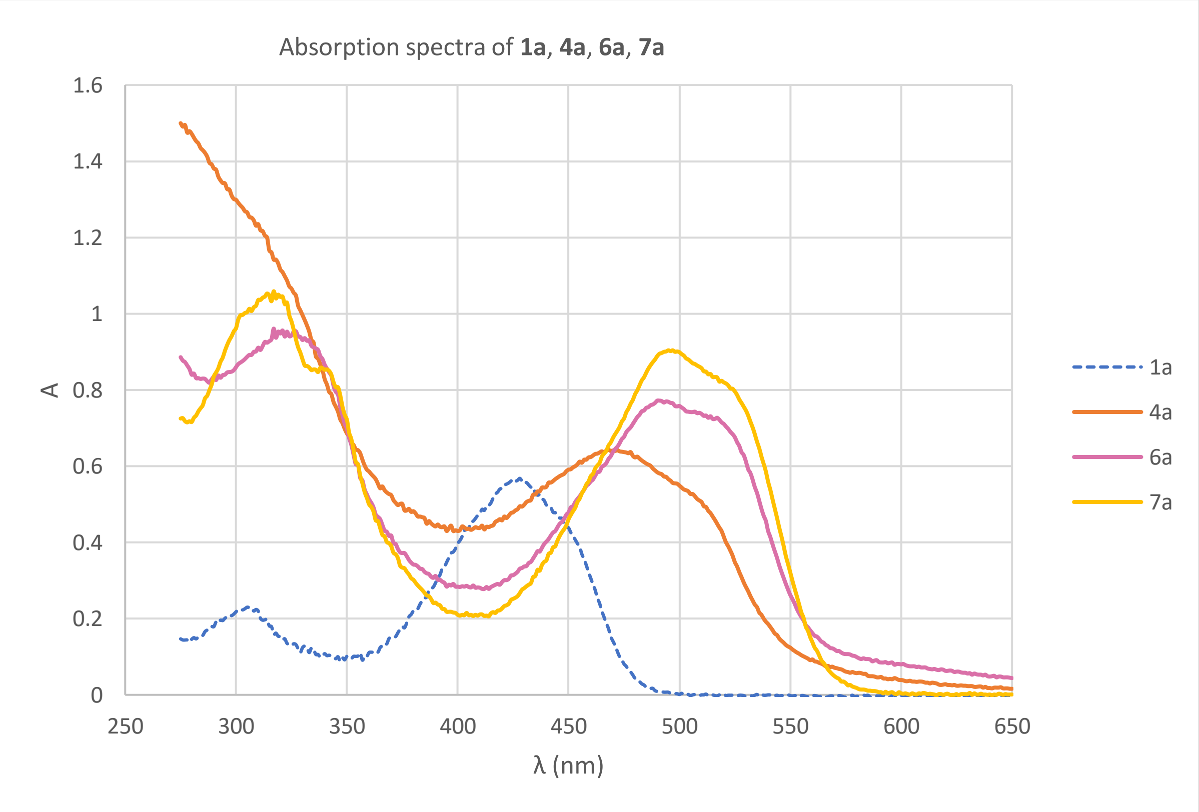

3.2. Comparison of the absorption spectra of **1b** and **4b**

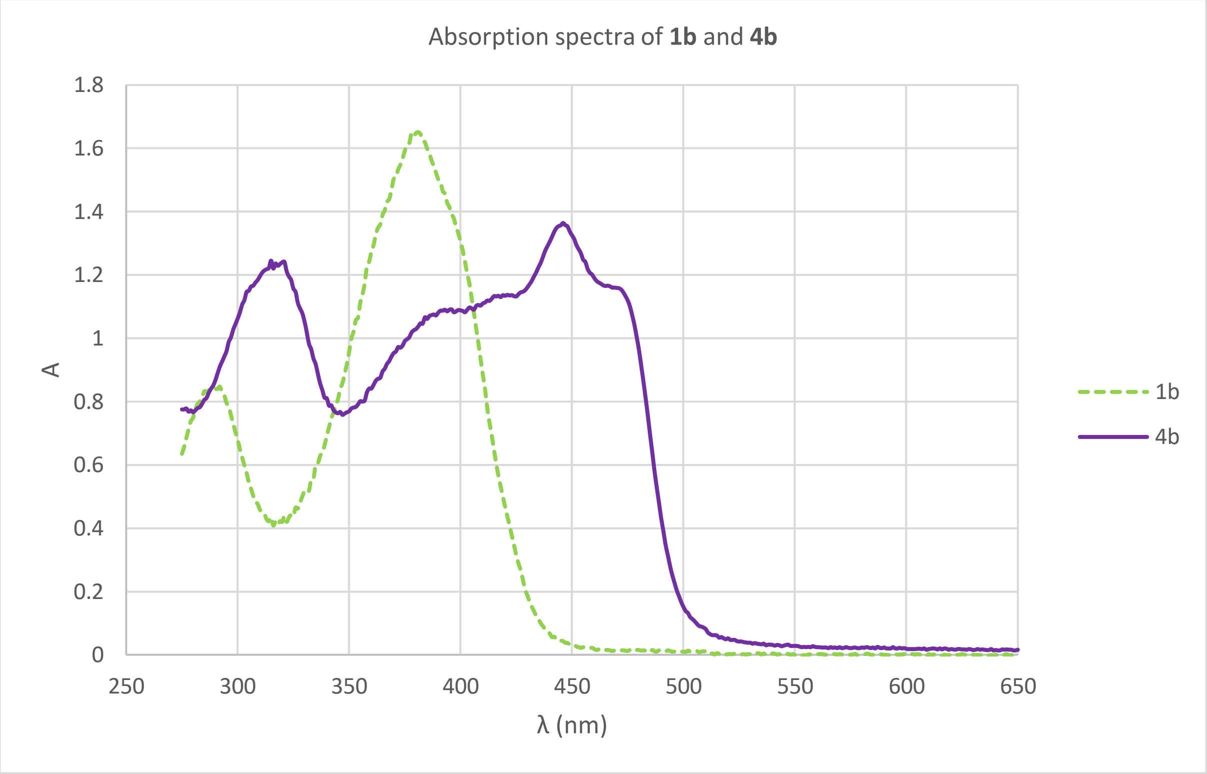

4. EXCITATION-EMISSION SPECTRA

4.1. Excitation and emission spectra of oxazolone **1a**

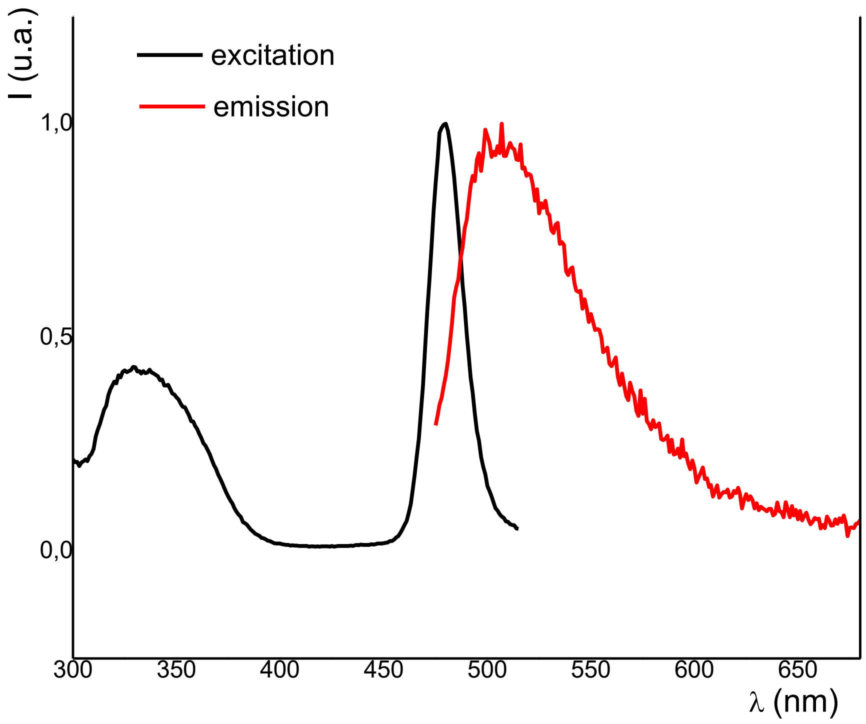

4.2. Excitation and emission spectra of complex **4a**

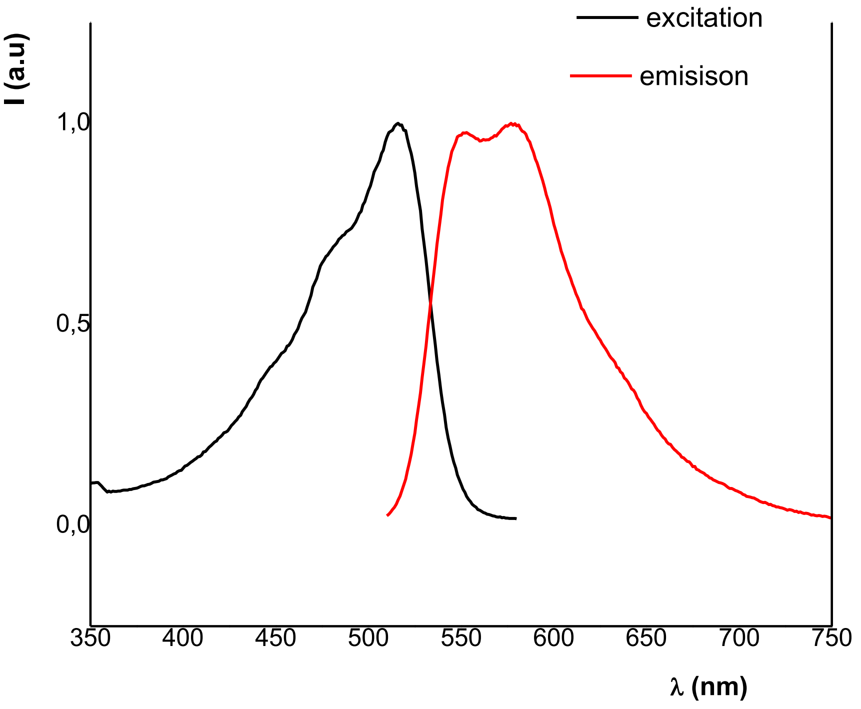

4.3. Excitation and emission spectra of complex **6a**

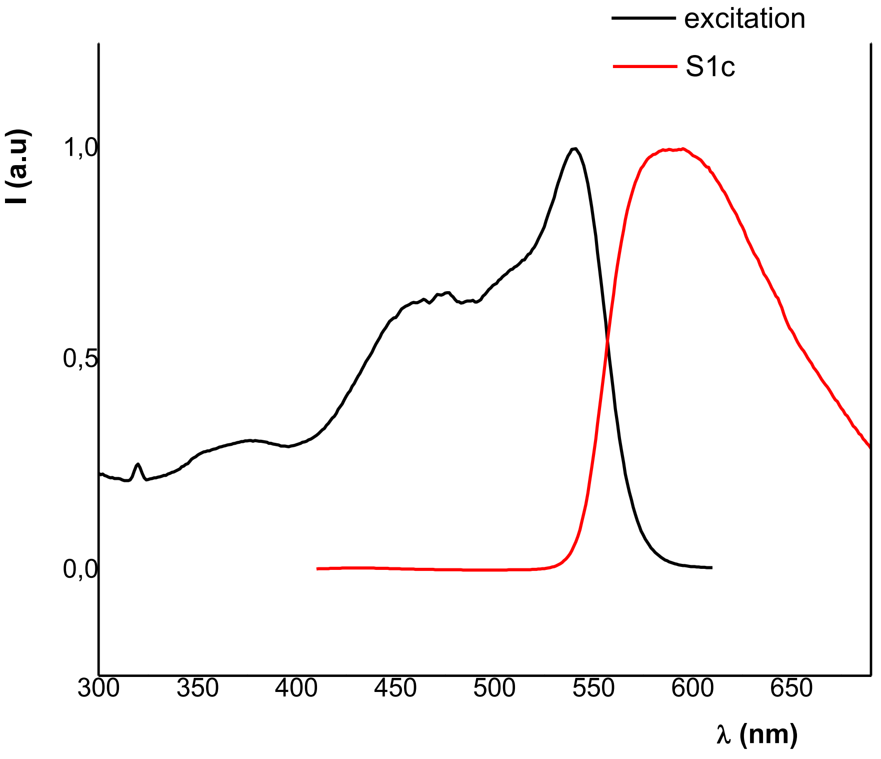

4.4. Excitation and emission spectra of complex **7a**

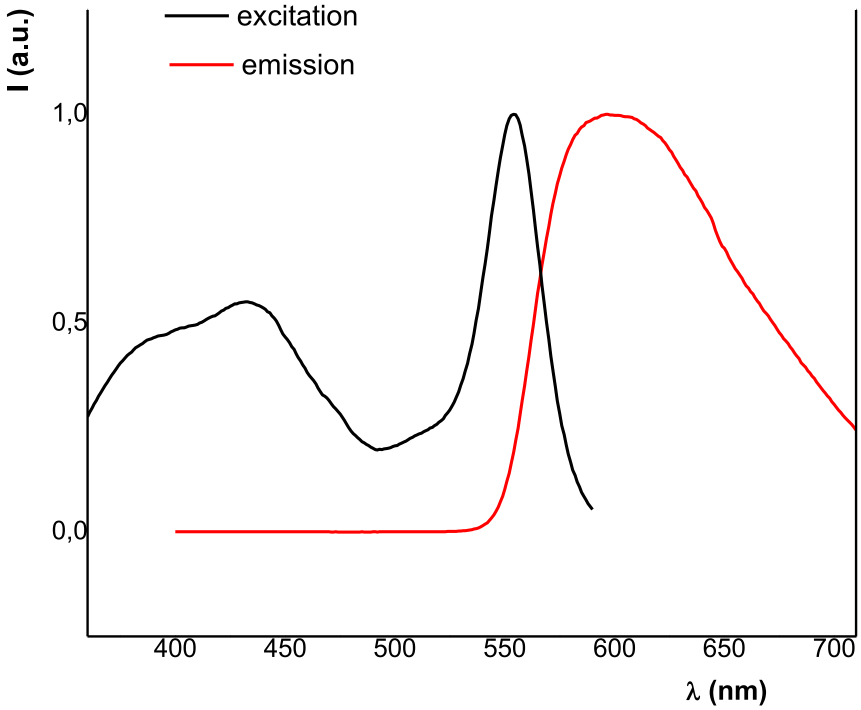

4.5. Excitation and emission spectra of complex **8a**

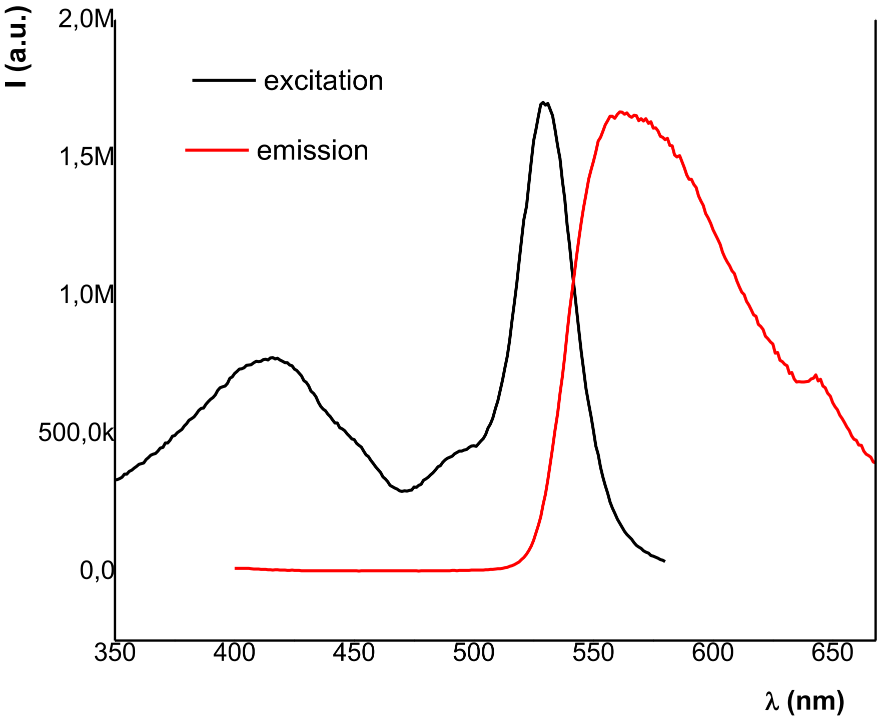

4.6. Excitation and emission spectra of oxazolone **1b**

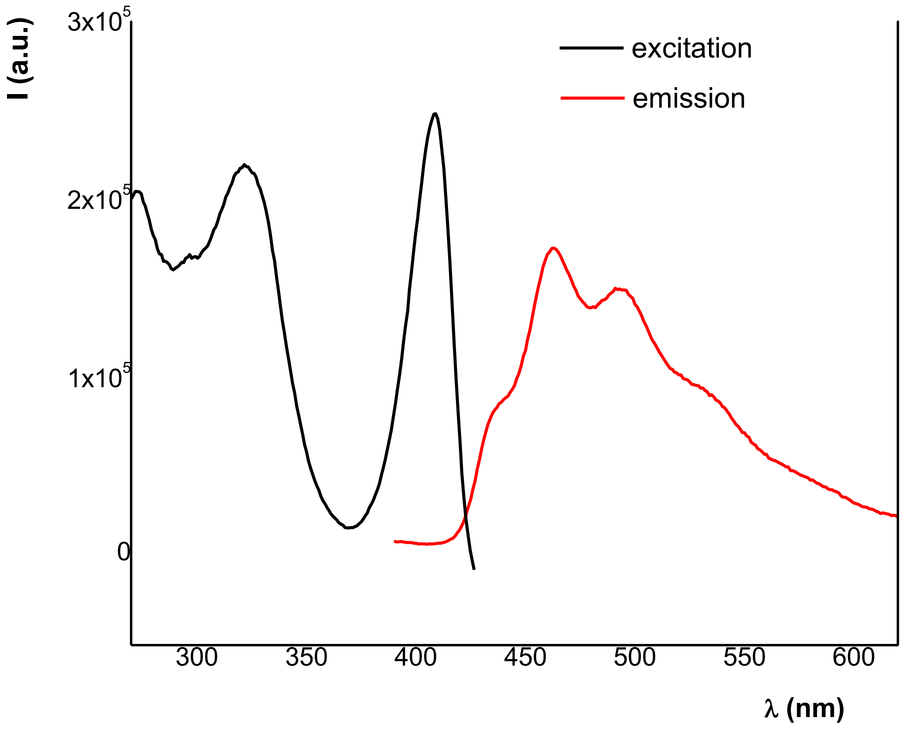

4.7. Excitation and emission spectra of complex **4b**

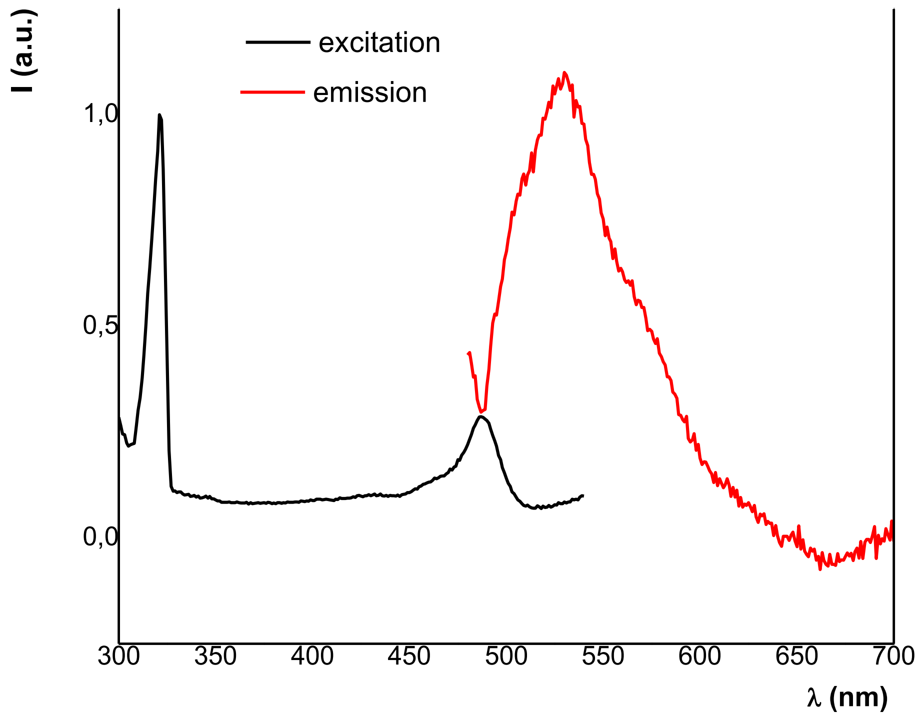

4.8. Excitation and emission of compound **6a** in CH_2Cl_2 10^{-6} M

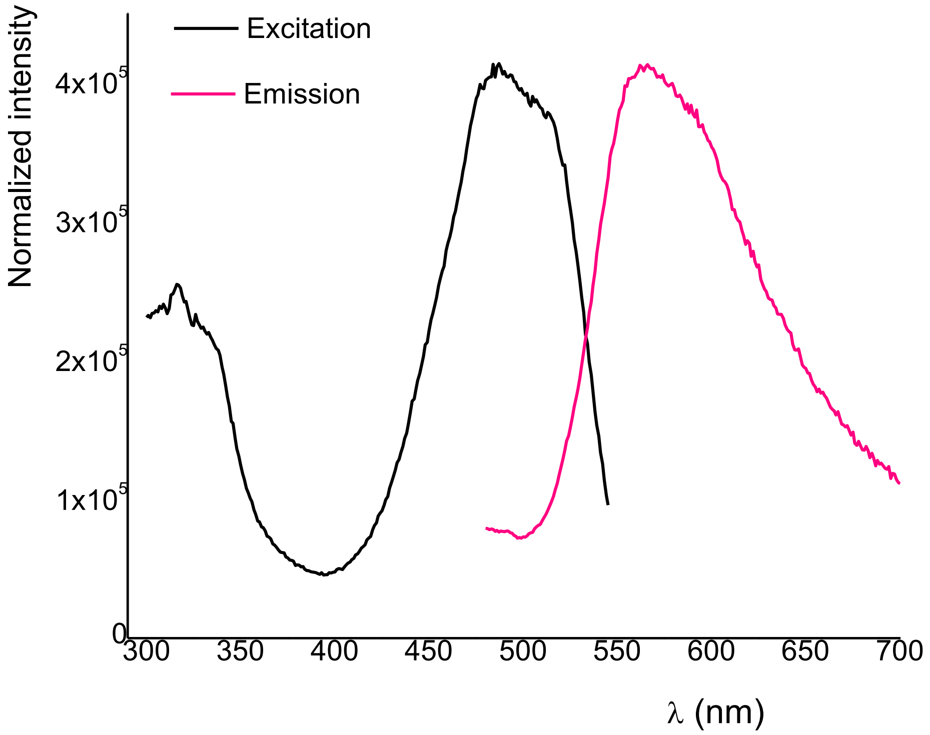

4.9. Excitation and emission of compound **6a** in CH_2Cl_2 10^{-5} M

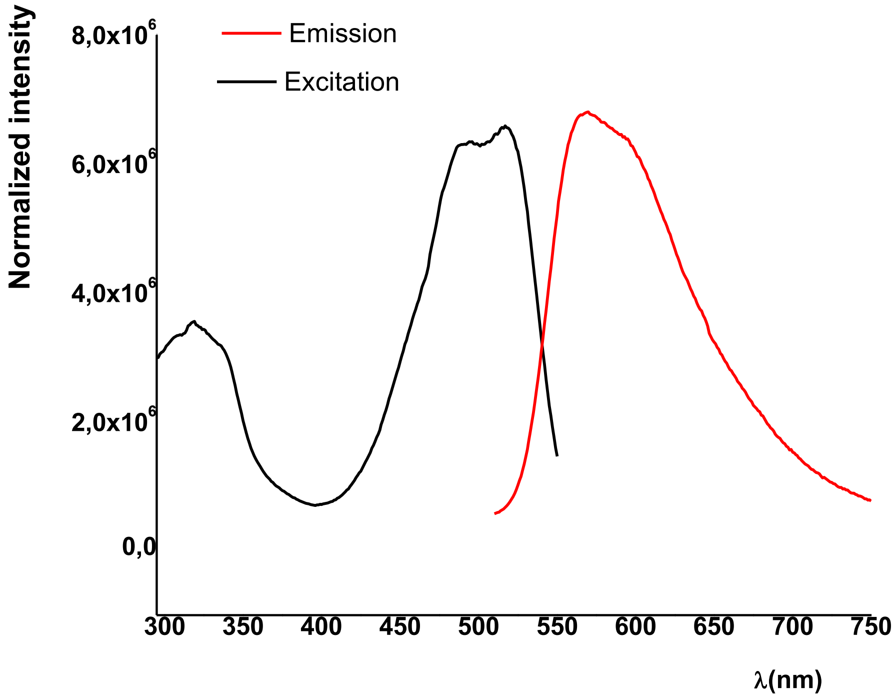

4.10. Absorption spectra of **6a** in CH_2Cl_2 10^{-5} M

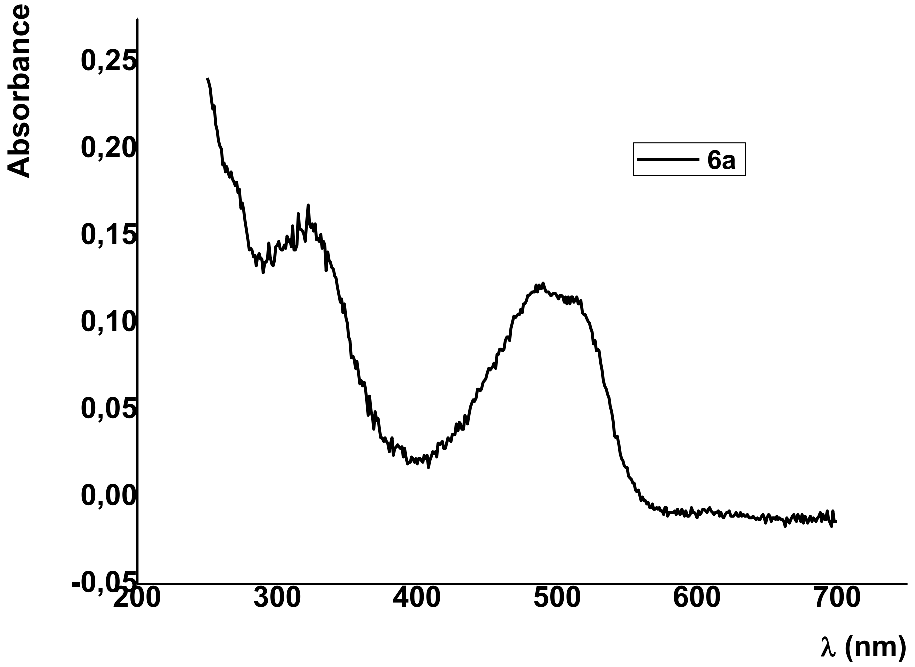

4.11. Absorption spectra of **6a** in CH_2Cl_2 10^{-6} M

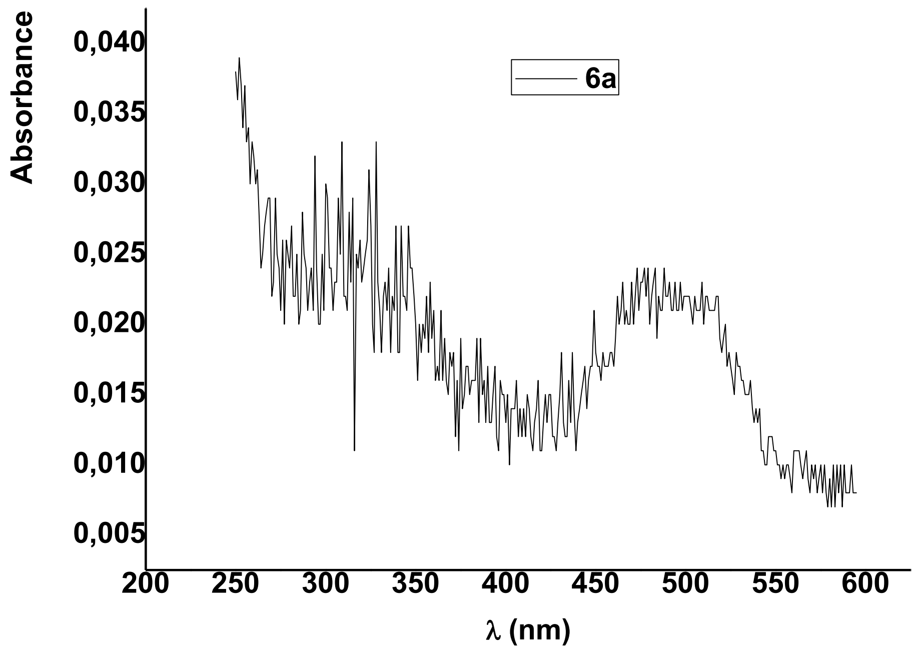

Table S1: Crystal data of 1a

Empirical formula	$C_{20}H_{17}NO_4$
Formula Weight	335.35
Temperature	100(2) K
Wavelength	0.71073 Å
Crystal system, space group	monoclinic, $P2_1/c$
Unit cell dimensions	a= 6.8670(19) Å<br>b= 12.547(3) Å β=97.365(5)°<br>c= 19.283(5) Å
Volume	1647.7(8) $Å^3$
Z	4
Absorption coefficient	0.095 mm^{-1}
F(000)	704
Crystal Size	0.075 x 0.110 x 0.180 mm
Absorption correction	Multi-scan
T_{min} , T_{max}	0.8418, 0.9585
θ_{min},θ_{max}	1.941, 26.881
Reflections collected / unique	17872 / 4765 [R(int) = 0.0870]
Completeness to θ_{max}	97.8% (98.6 % up to θ =25.24º)
Refinement method	Full-matrix least-squares on F^2
Data / restraints / parameters	4765 /0 / 294
Goodness-of-fit on F^2	1.092
Final R indices [I>2sigma(I)]	R1=0.0824; wR2=0.2338 [3180 refl.]
R indices (all data)	R1=0.1384; wR2=0.3049
Largest diff. peak and hole	0.344 / -0.298
Twin fraction	0.636/0.364

Table S2: Crystal data for complex 7a

Empirical formula	$C_{30}H_{24}N_3O_4Pd\cdot ClO_4\cdot 4(CH_2Cl_2)$
Formula Weight	1036.07
Temperature	100(2) K
Wavelength	0.71073 Å
Crystal system, space group	triclinic, $P\bar{1}$
Unit cell dimensions	a= 11.6874(6) Å α = 116.8470(10)°<br>b= 13.7722(7) Å β = 101.0960(10)°<br>c= 14.7559(7) Å γ = 95.0570(10) °
Volume	2037.52(18) $Å^3$
Z	2
Absorption coefficient	1.099 mm^{-1}
F(000)	1040
Crystal Size	0.070 x 0.130 x 0.135 mm
Absorption correction	Multi-scan
T_{min} , T_{max}	0.8403, 0.9144
θ_{min},θ_{max}	1.812, 28.981
Limiting indices	-15≤h≤15, -18≤k≤17, -20≤l≤19
Reflections collected / unique	25302 / 9851 [R(int) = 0.0310]
Completeness to θ_{max}	91.1% (99.8 % up to θ =25.24º)
Refinement method	Full-matrix least-squares on F^2
Data / restraints / parameters	9851 /4 / 497
Goodness-of-fit on F^2	1.033
Final R indices [I>2sigma(I)]	R1=0.0530; wR2=0.1110 [8353 refl.]
R indices (all data)	R1=0.0650; wR2=0.1204
Largest diff. peak and hole	2.491 / -2.487
